# Antivenom preclinical efficacy testing against Asian snakes and their availability in Asia: A systematic review

**DOI:** 10.1371/journal.pone.0288723

**Published:** 2023-07-19

**Authors:** Sutinee Soopairin, Chanthawat Patikorn, Suthira Taychakhoonavudh

**Affiliations:** Department of Social and Administrative Pharmacy, Faculty of Pharmaceutical Sciences, Chulalongkorn University, Bangkok, Thailand; Instituto Butantan, BRAZIL

## Abstract

**Background:**

Cross-neutralizing strategy has been applied to improve access to antivenoms, a key to reducing mortality and disability of snakebite envenoming. However, preclinical studies have been conducted to identify antivenoms’ cross-neutralizing ability when clinical studies may not be considered ethical. Therefore, this study aimed to identify and summarize scattered evidence regarding the preclinical efficacy of antivenoms against Asian snakes.

**Methodology/Principle findings:**

In this systematic review, we searched for articles published until May 30, 2022, in PubMed, Scopus, Web of Science, and Embase. Preclinical studies that reported the available antivenoms’ neutralizing ability against Asian snake lethality were included. Quality assessment was performed using the Systematic Review Centre for Laboratory animal Experimentation’s risk of bias tool and the adapted the Animal Research Reporting *In Vivo* Experiments guidelines. The availability of effective antivenoms against Asian snakes was analyzed by comparing data from included studies with snakebite-information and data platforms developed by the World Health Organization. Fifty-two studies were included. Most studies assessed the antivenom efficacy against snakes from Southeast Asia (58%), followed by South Asia (35%) and East Asia (19%). Twenty-two (49%) medically important snakes had antivenom(s) with confirmed neutralizing ability. Situation analyses of the availability of effective antivenoms in Asia demonstrated that locally produced antivenoms did not cover all medically important snakes in each country. Among countries without local antivenom production, preclinical studies were conducted only in Bangladesh, Sri Lanka, and Malaysia. Risk of bias assessment was limited in some domains because of unreported data.

**Conclusions/Significance:**

Cross-neutralizing of antivenoms against some medically important snakes in Asia was confirmed. This strategy may improve access to geographically effective antivenoms and bypass investment in novel antivenom development, especially in countries without local antivenom production. A database should be developed to aid the development of a snakebite-information system.

## Introduction

Snakebite envenoming is a neglected public health issue with high morbidity, disability, and mortality rates. Up to 1.8 million cases of envenoming are reported each year, and these cause up to 92,000 deaths annually. Mostly, this neglected issue shows with effect in the rural areas of low to middle-income countries that have insufficient financial support for patients suffering from snakebite envenoming. South Asia offers the highest rate of snakebite envenoming incidents, followed by Southeast Asia [1]. Despite its acute life-threatening symptoms, snakebite envenoming may also cause long-term complications leading to productivity loss. Moreover, snakebite envenoming is associated with higher disability-adjusted life years (DALYs) than those associated with other neglected tropical diseases such as dengue. Despite its higher burdens, snakebite envenoming receives fewer funds per DALY [2]. For example, the estimated economic burden of antivenoms is up to 13.8 million United States dollars in Sri Lanka and 2.5 billion in seven South East Asia countries [3–5]. Snakebite envenoming is a neglected tropical disease, although the World Health Organization (WHO) aims to halve snakebite-related deaths and disability by 2030 [6].

Antivenoms are the only effective treatment for snakebite envenoming that can reduce morbidity, disability, and mortality rates from this public health problem [7]. However, access to antivenoms has become an issue owing to their cost. This leads to unaffordability, and a shift to traditional treatment, which can result in reduced antivenom production and budget attenuation, increased antivenom prices, or halted antivenom production [8, 9]. To solve the problems of this neglected tropical disease, antivenom accessibility enhancement is a critical factor that can improve patient outcomes. Local or imported antivenoms with proven cross-neutralizing ability—the ability to neutralize against the toxic effects from the venom of different snake species, have not been included in the immunizing venom mixture, mainly those closely related species—are used as an alternative treatment if the specific antivenoms are unavailable [10–12]. Therefore, the use of antivenoms with proven cross-neutralization between antivenoms and snake venoms is a strategy for improving antivenom accessibility.

Most antivenoms available in the market had been registered without prior clinical studies in humans, while only a few were conducted. According to the WHO guidelines, neutralization of a lethal activity of antivenoms against snake venoms is an essential preclinical assay required in antivenom efficacy assessment, especially before use in humans and new geographical regions [13]. Hence, many preclinical studies have assessed the efficacy of antivenoms. However, the preclinical evidence of antivenom efficacy against each snake species in Asia—which accounts for a high incidence and death rates from snakebite envenoming—remains scattered [1].

Therefore, this study aimed to identify, review, and summarize the information about cross-neutralization and neutralization between available antivenoms and snake venoms in Asia, as reported in preclinical studies. Our results can be applied in the regulatory guidance for antivenom, where complete clinical studies may not be ethically applicable. This may serve as an initial step towards ensuring equal access to antivenoms across Asia.

## Methods

This study consisted of two parts. Part A was a systematic review summarizing cross-neutralization and neutralization data of antivenoms against Asian snakes and entailing a database search for a list of available antivenoms in Asia. Part B was an analysis of the availability of effective antivenoms in Asia.

### Part A: Systematic review conducted to retrieve cross-neutralization and neutralization data of antivenoms against Asian snakes

The systematic review methods were conducted following the Methodological Expectations of Cochrane Intervention Reviews and reported according to the Preferred Reporting Items for Systematic Reviews and Meta-Analyses (PRISMA) 2020 statement [14, 15]. The PRISMA checklist is provided in the **S1 Table**. The study protocol was registered at PROSPERO (CRD42022284543) [16].

#### Search strategy and eligibility criteria

Electronic bibliographic databases, including PubMed, Scopus, Web of Science, and Embase, were used to search for published articles related to cross-neutralization and neutralization between antivenoms and snake venoms. The search terms used in this review were ((Antivenom OR Antivenin OR Antivenene OR Anti-venom) AND Snake* AND Neutrali*), which were adjusted to match each database’s search strategy. All search terms were developed by SS under the supervision of CP and ST. An entire search strategy with results was provided in the **S2 Table**. The authors initially searched for published articles from inception until May 30, 2022. References searching was conducted to obtain some other related articles that were not included in the search. Grey literature was not searched in this review.

The inclusion criteria were preclinical studies conducted following the WHO guidelines using murine subjects, demonstrating *in vivo* cross-neutralizing activity and/or neutralizing ability of available antivenoms against the lethal activity of snake venoms originating from Asia. Case studies, cross-over studies, studies in other species apart from murine, *in vitro*, *ex vivo*, and in sillico studies were excluded. Studies using antivenoms not commercially available, such as experimental antivenoms and human IgG antibodies, were also excluded. Moreover, studies reporting only parameters indicating neutralization of toxic effects of snake venoms other than that of lethality were also excluded since they were supplementary preclinical assays [13]. No restrictions were placed on language.

#### Study selection and data extraction

The titles and abstracts of the studies were identified and independently screened by two reviewers (SS and CP). The full texts of all relevant studies were retrieved and independently assessed for eligibility by the two reviewers. Any discrepancies between both reviewers were resolved through discussion with a third reviewer (ST).

A standardized and pre-piloted data extraction form was used to independently extract data from the included studies using a Microsoft Excel spreadsheet for Mac (Microsoft, Redmond, WA, USA) by two reviewers (SS and CP). Discrepancies between both reviewers were resolved through discussion with the third reviewer (ST). The extracted information included study details, snake information, antivenom information, parameters indicating neutralization of lethality between antivenoms and snake venoms, and information for assessing the risk of bias.

#### Quality assessment

The two reviewers (SS and CP) independently conducted a risk of bias assessment of the included studies using the Systematic Review Centre for Laboratory animal Experimentation’s (SYRCLE) risk of bias tool for animal studies [17]. The SYRCLE’s risk of bias tool for animal studies contains 10 domains related to selection, performance, detection, attrition, reporting, and other biases. Moreover, an adapted Animal Research: Reporting of *In Vivo* Experiments (ARRIVE) guidelines, a guideline for reporting an *in vivo* experiment, was applied in the reporting quality assessment of the included studies in four domains: experiment set up, animals, procedure, and reported results [18].

#### Data synthesis

Extracted data were qualitatively synthesized using content analysis to summarize the included studies’ methodological characteristics. The summary revealed how well the preclinical studies assessing available antivenom-neutralizing efficacy against the lethality of Asian snake venoms had been conducted.

Extracted parameters indicating neutralization of lethal activity between available antivenoms and Asian snake venoms, such as median lethal dose (LD_50_), the amount of snake venoms that were intravenously or intraperitoneally injected, causing the deaths of 50% of mice in a group after 24–48 hours, were summarized. Median effective dose (ED_50_), the volume of antivenom that could protect 50% of mice intravenously or intraperitoneally injected with a challenge dose of snake venom (multiples of LD_50_ of venom), and potency of neutralization capacity (amount of snake venom in the mass unit that was neutralized per unit volume of antivenom) from each study, were summarized and presented. The cross-neutralizing and neutralizing abilities against medically important venomous snakes were demonstrated in a heat map to depict the efficacy of available antivenoms with the capability to neutralize the lethality of snake venoms in Asia [19]. Moreover, we reported the neutralizing ability against sea snakes and sea kraits, which are recognized by the WHO guidelines as snakes with potent venoms causing morbidity, disability, or death [19].

#### Part B: Analysis of effective antivenom availability in Asia

To gain insights into antivenom availability in Asia, data from the first part were combined with a list of available antivenoms from snakebite-information and data platforms, a new snakebite database developed by the WHO [20]. The authors identified Asian medically important venomous snakes based on the WHO guidelines [13]. Medically important venomous snakes were categorized into two categories. First, category one medically important venomous snakes (highest medical importance) were defined as those highly venomous snakes which were widespread in areas with large human populations and caused numerous snake bites, resulting in high morbidity, disability, or mortality. Second, category two medically important venomous snakes were defined as highly venomous snakes that can cause morbidity, disability, or death. Still, they were poorly known species or not a common cause of bites [13]. Next, we compared a list of available antivenoms with a list of medically important venomous snakes to analyze the situation of the availability of effective antivenoms against medically important venomous snakes in Asia. We summarized several medically venomous snakes in Asia from studies that reported antivenom cross-neutralizing and neutralizing abilities.

Then, we sorted the results by country of origin of the venom samples used in the experiments, as reported in the included studies, because antivenoms were applied to snakes originating in a different country. Those countries were sorted into different regions, Central Asia, East Asia, South Asia, and Southeast Asia, as listed in the WHO guidelines [19]. According to the WHO guidelines, countries listed in Central Asia consisted of Armenia, Azerbaijan, Georgia, Kazakhstan, Kyrgyzstan, Tajikistan, Uzbekistan, Turkmenistan, and Mongolia. In contrast, East Asia consists of China, Hong Kong, Japan, Taiwan, The Democratic People’s Republic of Korea (North Korea), and The Republic of Korea (South Korea). Afghanistan, Bangladesh, Bhutan, India, Nepal, Pakistan, and Sri Lanka were listed in the South Asia region. Lastly, Brunei Darussalam, Cambodia, Indonesia, The Lao People’s Democratic Republic (PDR), Malaysia, Myanmar, The Philippines, Singapore, Thailand, Timor-Leste, and Vietnam were listed in the Southeast Asia region [19].

## Results

### Study selection

**Fig 1:**
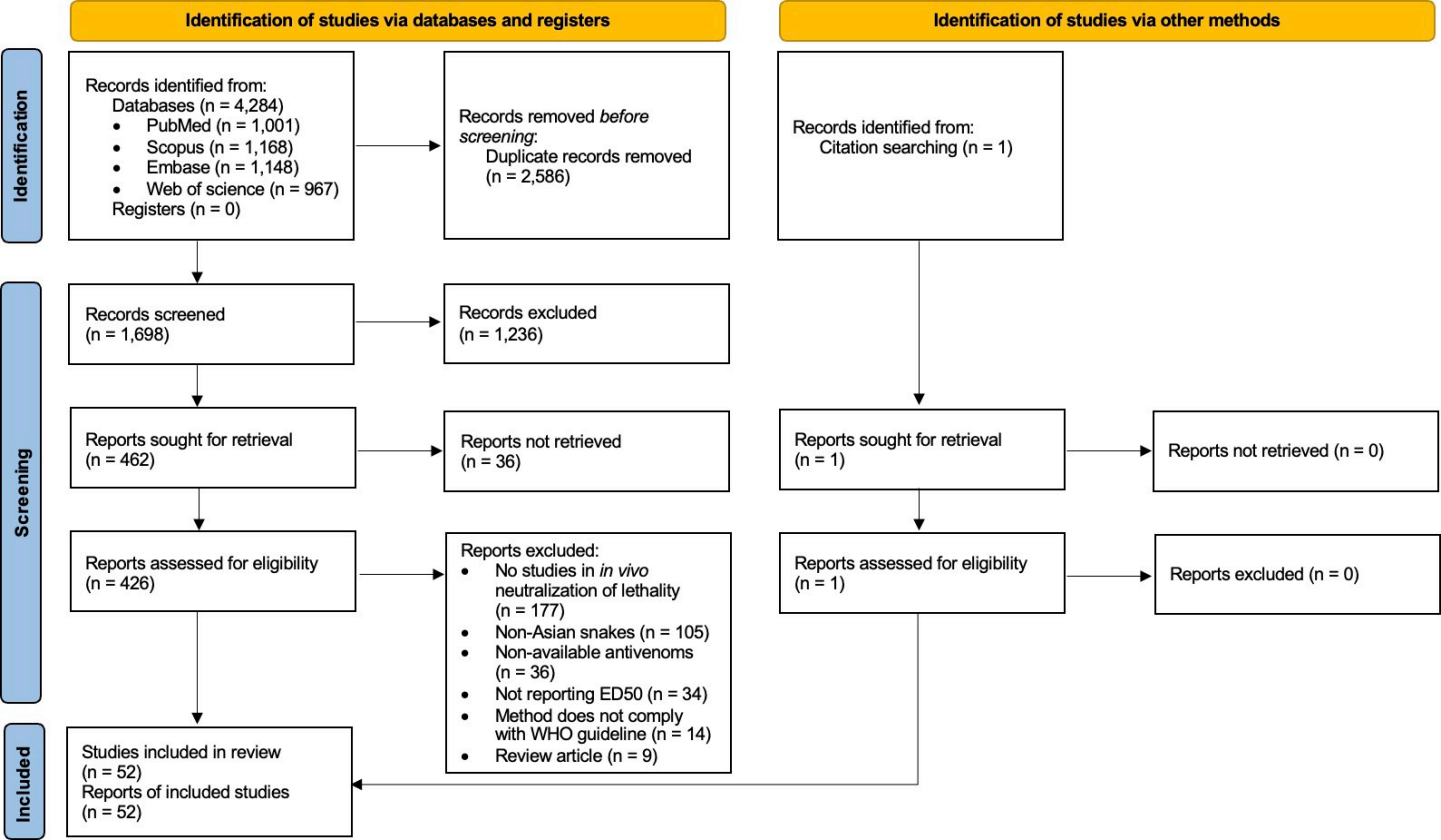
Study selection flow diagram.

A total of 4,284 articles were identified from four electronic databases using a discreet search strategy. A total of 2,586 duplicated articles were removed. The titles and abstracts of 1,698 articles were screened, and 426 full-text articles were assessed for eligibility. One study was identified from citation searching. Eventually, 52 eligible articles were included, as shown in **Fig 1**. No studies were retrieved from reference searching.

### Study characteristics

Snake species found in Asia were assessed to study antivenoms’ cross-neutralizing and neutralizing abilities against them, as summarized in the **S3 Table**. According to 52 included studies, snakes found in Southeast Asia, South Asia, and East Asia were tested in 30 studies (58%) [21–50], 18 (35%) [40–42, 51–65], and ten (19%) [24, 27, 30, 44, 66–71], respectively. However, no studies were found to assess snakes found in Central Asia. Thirty studies related to snakes found in Southeast Asia were mainly conducted on venomous snakes from Thailand [21, 24, 25, 27, 29, 30, 32, 34, 36–41, 43–47], and Malaysia [24–28, 34–37, 40–42, 48, 49]. Concurrently, all studies related to snakes from South Asia were conducted on venomous snakes found in India (10 studies) [40, 41, 52–56, 59, 60, 62], and Sri Lanka (nine studies) [40–42, 51, 52, 57, 62, 64, 65].

Regarding the snake family, 32 studies (62%) included antivenom cross-neutralizing and neutralizing abilities against snakes in the Elapidae family and 24 (46%) in the Viperidae family. Four (8%) studies were conducted on sea snakes in Asia [35, 44, 48, 49]. While one study (2%) was conducted on sea krait in Asia [50]. The most frequently tested snake venom was *Naja kaouthia* (10 from 52 studies [19%]) [32, 34, 36, 40, 41, 44, 46, 47, 59, 60]. Among polyvalent antivenoms from Asia assessed in the included articles, the neuro-polyvalent snake antivenom from Queen Saovabha Memorial Institute (QSMI), Thailand, was the most frequently tested polyvalent antivenoms, which were reported in seven (13%) studies [33–36, 41, 70, 72]. In contrast, a cobra antivenom from QSMI, Thailand, was the most frequently tested monovalent antivenom, which was reported in ten (19%) studies [32, 34, 35, 38, 41, 44, 46, 47, 53, 63].

### Neutralizing and cross-neutralizing abilities of available antivenoms against the lethality of medically important venomous snakes in Asia from preclinical studies

All neutralizing and cross-neutralizing abilities of antivenoms against the lethality of medically important venomous snakes in Asia from the included preclinical studies are summarized in **Table 1**. The neutralizing and cross-neutralizing abilities differed among antivenoms and varied between snake venoms. More details of the strength of these abilities, such as ED_50_, are reported in the **S4 Table**. ED_50_ is a median effective dose of antivenoms that reflects the preclinical efficacy of antivenoms. Units of ED_50_ were differently applied across the included studies. Microliter (μL) was used as a unit of ED_50_ in 40 studies (77%). Moreover, the LD_50_ value used for deriving ED_50_ differs in each experiment, even for the same snake species. Thereby, a meta-analysis of ED_50_ cannot be performed in our study.

**Table 1:** Neutralizing and cross-neutralizing abilities of available antivenoms against the lethality of medically important venomous snakes in Asia according to preclinical evidence.

Snake species / Antivenom	Agkistrodon acutus antivenin; SSBT	Bungarus multicinctus antivenin; SSBT	Neurobivalent antivenom; NIPM	Doboia siamensis monovalent antivenom; NIPM	Monovalent Antivenin Snorkel Viper; NIPM	Snake antivenin I.P. (Asia): Haffkine	Snake Venom Antiserum (Polyvalent); Bharat		Snake venom antiserum I.P.; Premium		Snake Venom Antiserum I.P. (Asia); VINS	Polyvalent Antisnake Venom Serum; NIH	Serum Anti Bisa Ular Polivalen (SABU); Persero	Anti-Viper (Russell’s viper); MPF	Naja philippinensis Antivenin; RITM	Banded krait antivenin; QSMI	Cobra Antivenin; QSMI	Green Pit Viper Antivenin; QSMI	Hemato-polyvalent snake antivenom; QSMI	Malayan pit viper antivenin; QSMI	Neuro-polyvalent snake antivenom; QSMI	Russell’s viper antivenin; QSMI	Refined earth tiger snake antivenom; IVAC
**Elapidae**																							
**East Asia**																							
*B*. *multicinctus*		**#**	**#**																				
*N*. *atra*			**#**																				
**South Asia**																							
*B*. *caeruleus*						**#**	**#**		**#**		**#**	**#**											
*B*. *sindanus*																							
*N*. *kaouthia*																							
*N*. *naja*						**#**	**#**	**#**	**#**	**#**	**#**	**#**											
**Southeast Asia**																							
*B*. *candidus*																					**#**		
*N*. *kaouthia*																	**#**				**#**		**#**
*N*. *philippinensis*															**#**								
*N*. *samarensis*																							
*N*. *siamensis*																							
*N*. *sputatrix*													**#**										
*N*. *sumatrana*																							
**Viperidae**																							
**East Asia**																							
*D*. *acutus*	**#**				**#**																		
**South Asia**																							
*D*. *russelii*						**#**	**#**		**#**		**#**	**#**											
*E*. *carinatus*						**#**	**#**		**#**		**#**	**#**											
*H*. *hypnale*																							
**Southeast Asia**																							
*C*. *rhodostoma*													**#**						**#**	**#**			
*T*. *albolabris*																		**#**	**#**				
*T*. *erythrurus*																							
*T*. *insularis*																							
*D*. *siamensis*														***#***					**#**			**#**	

Hashtag (#) shows that those snake venoms have been added to the immunizing mixture for antivenom development.

The green color shows that medically important venomous snakes in Asia have been tested and confirmed on neutralizing and cross-neutralizing abilities against antivenoms with various degrees of effectiveness.

The red color shows that medically important venomous snakes in Asia have been tested and confirmed with no neutralizing and cross-neutralizing abilities of antivenoms against those snakes.

The gray color shows that no studies have been found on those medically important venomous snakes and antivenoms in Asia.

**Abbreviations**: Bharat–Bharat Serums and Vaccines Limited; Haffkine–Haffkine Biopharmaceutical Corporation Ltd; IVAC–Institute of Vaccines and Biological Substances; MPF–Myanmar Pharmaceutical Factory; NIH–National Institute of Health; NIPM–National Institute of Preventive Medicine; Persero–PT Bio Farma; Premium–Premium Serums and Vaccines Pvt. Ltd.; QSMI–Queen Saovabha Memorial Institute; RITM–Research Institute for Tropical Medicine (Biologicals Manufacturing Division); SSBT–Shanghai Serum Bio-technology Co Ltd; VINS–VINS Bioproducts Ltd

According to 45 medically important venomous snakes found in Asia identified by the WHO, the authors found that only 22 (49%) medically important venomous snakes were tested and confirmed with neutralizing ability of antivenoms against their lethality. Furthermore, the ineffectiveness of antivenoms against six (13%) medically important venomous snakes was found.

#### Medically important elapids of Asia

No medically important elapids in Central Asia were tested in the included studies.

As demonstrated in **Table 1**, *Bungarus multicinctus* (many-banded krait), found in China, was tested against its specific antivenom, *B*. *multicinctus* antivenin, from Shanghai Serum Bio-technology Co. Ltd. in two studies. These studies reported the ED_50_ in different units; one showed the ED_50_ value of 1.65 μL, while another showed a value of 17.68 μg/g [67, 68]. This antivenom was also tested against *B*. *multicinctus* found in Taiwan, exhibiting neutralizing ability with the ED_50_ value of 4.13 μL. A neurobivalent antivenom from the National Institute of Preventive Medicine, Taiwan, was tested against *B*. *multicinctus* found in China and Taiwan, providing the ED_50_ value of 8.92 μL and 33.39 μL, respectively [67].

The lethality of *Naja atra* (Chinese cobra) found in China is not cross-neutralized by *B*. *multicinctus* antivenin from Shanghai Serum Bio-technology Co. Ltd., China, providing the ED_50_ value at > 800 μg/g [68]. Moreover, the lethality of *N*. *atra* found in Taiwan was neutralized by the neurobivalent antivenom from the National Institute of Preventive Medicine, Taiwan, exhibiting the ED_50_ value of 101.82 mg/g, ranging from 86.97–119.17 mg/g. It is cross-neutralized by a neuro-polyvalent snake antivenom from QSMI, Thailand, resulting in the ED_50_ value of 9.70 mg/g, ranging from 9.28–11.35 mg/g, and cross-neutralized by a refined earth tiger snake antivenom from the Institute of Vaccines and Biological Substances (IVAC), Vietnam, with the ED_50_ value of 17.41 mg/g, ranging from 14.87–20.38 mg/g [70]. However, a *Daboia siamensis* monovalent antivenom from the Center for Disease Control, Taiwan, was ineffective against the lethality of *N*. *atra* found in Taiwan [70].

For South Asian snakes, *Bungarus caeruleus* (common krait) found in India lethality was neutralized by snake venom antiserum I.P. from Premium Serums and Vaccines Pvt. Ltd., India, resulting in the ED_50_ value at 26.17 μL, ranging from 19.36–35.37 μL [59]. The lethality of *B*. *caeruleus* in India was also neutralized by snake venom antiserum I.P. (Asia) from VINS Bioproducts Ltd., India, providing the ED_50_ value of 17.14 μL [62]. In contrast, the lethality of *B*. *caeruleus* found in Sri Lanka was neutralized by snake venom antiserum I.P. (Asia) from VINS Bioproducts Ltd. and snake venom antiserum (polyvalent) from Bharat Serums and Vaccines Limited from India, exhibiting ED_50_ value of 3.92 μL and 2.93 μL, respectively [65]. Moreover, the lethality of *B*. *caeruleus* found in Pakistan can be neutralized by snake venom antiserum I.P. (Asia) from VINS Bioproducts Ltd., India, providing the ED_50_ value of 16.53 μL [62].

*Bungarus sindanus* (Sind krait) found in India lethality was cross-neutralized by snake venom antiserum I.P. from Premium Serums and Vaccines Pvt. Ltd., India, exhibiting the ED_50_ value of 5.43 μL, ranging from 4.34–6.51 μL [59]. In contrast, *B*. *sindanus* from Pakistan can be cross-neutralized by snake venom antiserum I.P. (Asia) from VINS Bioproducts Ltd., India, providing the ED_50_ value of 13.29 μL [58].

For *N*. *kaouthia* (monocled cobra) found in India, its lethality was cross-neutralized by snake antivenin I.P. (Asia) from Haffkine Biopharmaceutical Co. Ltd., India, snake venom antiserum (polyvalent) from Bharat Serums and Vaccines Limited, India, and snake venom antiserum I.P. (Asia) from VINS Bioproducts Ltd., India, with ED_50_ value of 112.66 ± 5.11 mg/g, 92.68 ± 4.68 mg/g, and 76.38 ± 3.48 mg/g, respectively [60]. However, the lethality of *N*. *kaouthia* found in India was not cross-neutralized by snake venom antiserum I.P. from Premium Serums and Vaccines Pvt. Ltd., India [59]. However, the lethality of *N*. *kaouthia* in Bangladesh was cross-neutralized by snake antivenin I.P. (Asia) from Haffkine Biopharmaceutical Co. Ltd., India, snake venom antiserum (polyvalent) from Bharat Serums and Vaccines Limited, India, and snake venom antiserum I.P. (Asia) from VINS Bioproducts Ltd., India, with ED_50_ value of 137.23 ± 4.42 mg/g, 97.28 ± 2.46 mg/g, and 94.62 ± 4.52 mg/g, respectively [60].

For *Naja naja* (Indian cobra) found in India, its lethality can be neutralized by its specific antivenoms; snake venom antiserum (polyvalent) from Bharat Serums and Vaccines Limited, India [53], snake venom antiserum I.P. from Premium Serums and Vaccines Pvt. Ltd., India [53, 55, 59], and snake venom antiserum I.P. (Asia) from VINS Bioproducts Ltd., India with various degrees of effectiveness [40]. However, snake venom antiserum I.P. from Premium Serums and Vaccines Pvt. Ltd., India, showed ineffectiveness against the lethality of *N*. *naja* inhibited in different areas of India [55]. Furthermore, another study found that snake venom antiserum (polyvalent) from Bharat Serums and Vaccines Limited, India was ineffective against the lethality of *N*. *naja* found in India [40]. Indian *N*. *naja* lethality can also be cross-neutralized by the neuro-polyvalent snake antivenom from QSMI, Thailand, with the ED_50_ value of 156.57 μL and 200 μL in two experiments [41]. The lethality of *N*. *naja* in Pakistan was cross-neutralized by the neurobivalent antivenom from the National Institute of Preventive Medicine, Taiwan, snake venom antiserum I.P. (Asia) from VINS Bioproducts Ltd., India, and cobra antivenin from QSMI, Thailand in which reported with the ED_50_ values of 75 μL, 32.77 μL, and 18 μL, respectively [63]. Moreover, the lethality of *N*. *naja* found in Sri Lanka can be neutralized by snake venom antiserum (polyvalent) from Bharat Serums and Vaccines Limited, India, and snake venom antiserum I.P. (Asia) from VINS Bioproducts Ltd., India with various degrees of effectiveness [40, 51, 64, 65]. In contrast, the neuro-polyvalent snake antivenom from QSMI, Thailand can cross-neutralize the lethality of *N*. *naja* found in Sri Lanka with the ED_50_ values of 89.88 μL and 100.00 μL in two experiments [41]. Nevertheless, a study on snake venom antiserum (polyvalent) from Bharat Serums and Vaccines Limited, India, showed ineffective against *N*. *naja* from Sri Lanka [40].

In Southeast Asia, the lethality of *Bungarus candidus* (Malayan krait) found in Thailand was cross-neutralized by banded krait antivenin from QSMI, Thailand, with the ED_50_ value of 319.70 μL, ranging from 251.80–406.00 μL [45]. The lethality of *B*. *candidus* found in Java Island, Indonesia, was cross-neutralized by serum anti bisa ular (SABU) polivalen (Bio Save) from PT Bio Farma (Persero), Indonesia, with the ED_50_ value of 111.25 μL and the neuro-polyvalent snake antivenom from QSMI, Thailand, with the ED_50_ value of 5.56 μL [33]. The lethality of *B*. *candidus* found in Malaysia can be neutralized by the neuro-polyvalent snake antivenom from QSMI, Thailand, providing the ED_50_ value of 13.91 μL [41].

For *N*. *kaouthia* (monocled cobra), studies confirmed that Thai *N*. *kaouthia* lethality could be neutralized by its specific monovalent antivenom, cobra antivenin [32, 34, 41, 44, 46, 47], and its specific polyvalent antivenom, the neuro-polyvalent snake antivenom from QSMI, with various degrees of effectiveness [34, 36]. The lethality of *N*. *kaouthia* from Malaysia can be neutralized by cobra antivenin and the neuro-polyvalent snake antivenom from QSMI, with the ED_50_ value of 78.29 μL and 70.68 μL, respectively [34]. In addition, another study reported that both antivenoms could neutralize against *N*. *kaouthia* found in Malaysia with a similar ED_50_ value of 150 μL [41]. These two antivenoms can also cross-neutralize against the lethality of *N*. *kaouthia* found in Vietnam with the ED_50_ value of 120.86 μL and 89.89 μL [34]. Moreover, snake venom antiserum I.P. (Asia) from VINS Bioproducts Ltd., India, can be cross-neutralized against the lethality of *N*. *kaouthia* venom found in Thailand and Malaysia, reporting ED_50_ values of 75 μL and 70.7 μL, ranging from 68.5–82.1 μL and 64.1–92.3 μL, respectively [40]. Conversely, another antivenom developed in India, snake venom antiserum (polyvalent) from Bharat Serums and Vaccines Limited, can only cross-neutralize against the lethality of *N*. *kaouthia* venom found in Thailand with the ED_50_ value of 55.6 μL, ranging from 36.6–84.5 μL. It was ineffective against the lethality of *N*. *kaouthia* venom from Malaysia [40].

For *Naja philippinensis* (Philippine cobra) found in the Philippines, it was found that its specific antivenom, monovalent (*Naja philippinensis*) cobra antivenin from Biologicals Manufacturing Division (Research Institute for Tropical Medicine), Philippines, can neutralize its lethality, resulting in the ED_50_ value of 44.94 μL, ranging from 20.6–69.23 μL [22]. Moreover, this antivenom can cross-neutralize against the lethality of *Naja samarensis*, providing the ED_50_ value of 120.86 μL, ranging from 104.79–139.40 μL [22].

For *Naja siamensis* (Indo-Chinese spitting cobra) in Thailand, cobra antivenin from QSMI can cross-neutralize against its lethality with the ED_50_ value of 91.6 μL, ranging from 66.2–126.8 μL [46].

*Naja sputatrix* (Javan spitting cobra) found in Indonesia can be neutralized by its specific antivenom, SABU polivalen (Bio Save) from PT Bio Farma (Persero), Indonesia, with the ED_50_ value of 111.25 μL [33]. It can be cross-neutralized by the neuro-polyvalent snake antivenom from QSMI exhibiting the ED_50_ value of 50 μL [33, 36], similar to *N*. *sputatrix* found in Malaysia reporting the ED_50_ value of 136.72 μL [72].

Lastly, *Naja sumatrana* (Equatorial spitting cobra) found in Sumatra Island, Indonesia, can be cross-neutralized by SABU polivalen (Bio Save) from PT Bio Farma (Persero), Indonesia, with the ED_50_ value of 156.57 μL, and the neuro-polyvalent snake antivenom from QSMI, Thailand with the ED_50_ value of 55.63 μL [33]. This is the same with *N*. *sumatrana* found in Malaysia, which can also be cross-neutralized by the neuro-polyvalent snake antivenom from QSMI, Thailand, and exhibits the ED_50_ value of 25 μL [36]. Additionally, two antivenoms from India, snake venom antiserum (polyvalent) from Bharat Serums and Vaccines Limited and snake venom antiserum I.P. (Asia) from VINS Bioproducts Ltd., were cross-neutralized against the lethality of *N*. *sumatrana* in Malaysia, with the ED_50_ value of 150 μL and 39.1 μL, ranging from 37.1–164.2 μL and 32–47.9 μL, respectively [40].

#### Medically important venomous vipers of Asia

No medically important venomous vipers in Central Asia were found being tested in the included studies. However, these true and pit vipers from East, South, and Southeast Asia were tested in the included studies as outlined in **Table 1**.

*Medically important true vipers of Asia*. In South Asia, three medically important venomous snakes in the Viperidae family were tested in the included studies. There were studies confirmed preclinical efficacy of snake venom antiserum (polyvalent) from Bharat Serums and Vaccines Limited [56], snake venom antiserum I.P. from Premium Serums and Vaccines Pvt. Ltd. [54], and snake venom antiserum I.P. (Asia) from VINS Bioproducts Ltd. [52]. These are specific antivenoms against *Daboia russelii* (Russell’s viper) found in India which can neutralize its lethality. While the lethality of *D*. *russelii* found in Sri Lanka can be neutralized by snake venom antiserum (polyvalent) from Bharat Serums and Vaccines Limited, India, with the median effective ratio (ER_50_) value of 1.24 mg/mL [65]. It can also be neutralized by polyvalent antivenoms from India, snake venom antiserum I.P. from Premium Serums and Vaccines Pvt. Ltd. with the ER_50_ value of 2.33 mg/mL [57], and snake venom antiserum I.P. (Asia) from VINS Bioproducts Ltd. with various degrees of effectiveness [52, 57, 64, 65]. The hemato-polyvalent snake antivenom from QSMI, Thailand, can also neutralize the lethality of *D*. *russelii* in Sri Lanka with the ED_50_ value of 7.52 μL, ranging from 3.53–15.30 μL [42]. Moreover, *D*. *russelii* from Bangladesh can be neutralized by snake venom antiserum (polyvalent) from snake venom antiserum I.P. from Premium Serums and Vaccines Pvt. Ltd. and snake venom antiserum I.P. (Asia) from VINS Bioproducts Ltd., India with the ER_50_ value < 1.50 mg/mL [57]. These two antivenoms can also neutralize the lethality of *D*. *russelii* found in Pakistan, exhibiting the ER_50_ value of 2.66 mg/mL and 1.86 mg/mL [57, 61].

*Echis carinatus* (saw-scaled viper) found in India can be neutralized against lethality by its specific antivenom, including snake venom antiserum (polyvalent) from Bharat Serums and Vaccines Limited, India [56], and snake venom antiserum I.P. from Premium Serums and Vaccines Pvt. Ltd., India [59]. In contrast, the lethality of *E*. *carinatus* found in Sri Lanka was neutralized by snake venom antiserum (polyvalent) from Bharat Serums and Vaccines Limited, India, and snake venom antiserum I.P. (Asia) from VINS Bioproducts Ltd. India with ER_50_ value reported at 2.82 mg/mL and 2.79 mg/mL [65]. Moreover, it was found that the lethality of *E*. *carinatus* found in Pakistan cannot be cross-neutralized by the hemato-polyvalent snake antivenom from QSMI, Thailand [42].

Lastly, in Southeast Asia, *Daboia siamensis* (Eastern Russell’s viper) found in Myanmar can be cross-neutralized by the hemato-polyvalent snake antivenom with the ED_50_ value of 35.36 μL [37], and Russell’s viper antivenin from QSMI, Thailand, resulting with the ED_50_ of 60 μL, ranging from 40.58–88.70 μL [30]. *D*. *siamensis* found in Thailand had specific antivenoms, the hemato-polyvalent snake antivenom and Russell’s viper antivenin from QSMI, Thailand, which were neutralized against its lethality through various ED_50_ values [29, 30, 37, 39]. However, SABU polivalen (Bio Save) from PT Bio Farma (Persero), Indonesia was ineffective against both lethality of *D*. *siamensis* from Thailand and Indonesia [29]. In contrast, Russell’s viper antivenin from QSMI, Thailand can be cross-neutralized against the lethality of *D*. *siamensis* from Thailand and Indonesia with the ED_50_ value of 9.5 μL and 6.64 μL, respectively [29].

*Medically important pit vipers of Asia*. In East Asia, *Deinagkistrodon acutus* (sharp-nosed pit viper) found in China was tested against *Agkistrodon acutus* antivenin from Shanghai Serum Bio-technology Co Ltd. and monovalent antivenin snorkel viper from the National Institute of Preventive Medicine, Taiwan exhibiting the ED_50_ value of 32 μL and 5 μL, respectively [66]. Taiwanese *D*. *acutus* was tested against *A*. *acutus* antivenin from Shanghai Serum Bio-technology Co Ltd. and monovalent antivenin snorkel viper from the National Institute of Preventive Medicine, Taiwan, exhibited the ED_50_ value of 6.88 μL and 2.78 μL, respectively [66]. The results showed that both antivenoms could neutralize the lethality of both *D*. *acutus* in China and Taiwan.

Regarding South Asia pit vipers, *Hypnale hypnale* (hump-nosed pit viper) lethality found in Sri Lanka can be cross-neutralized by snake venom antiserum I.P. (Asia) from VINS Bioproducts Ltd., India [64]. Its lethality can be cross-neutralized by the hemato-polyvalent snake antivenom from QSMI with the ED_50_ value of 41.53 μL, ranging from 20.4–88.4 μL [42], and Malayan pit viper antivenin from QSMI with the ED_50_ value of 70.71 μL, ranging from 33.7–148.4 μL [42].

For Southeast Asia pit vipers, *Calloselasma rhodostoma* (Malayan pit viper) found in Java Island, Indonesia, was neutralized by its specific polyvalent antivenom, SABU polivalen (Bio Save) from PT Bio Farma (Persero), Indonesia [33]. It can be neutralized by the hemato-polyvalent snake antivenom from QSMI, Thailand, with the ED_50_ value of 18.75 μL and 11.2 μL reported in two studies [33, 37]. The lethality of *C*. *rhodostom*a found in Malaysia can also be neutralized by the hemato-polyvalent snake antivenom from QSMI, Thailand, with the ED_50_ value of 22.47 μL [37, 42], and Malayan pit viper antivenin from QSMI with the ED_50_ value of 41.53 μL, ranging from 20.4–88.4 μL [42].

*Trimeresurus albolabris* (white-lipped green pit viper) found in Thailand had a specific antivenom, green pit viper antivenin from QSMI, Thailand, where its efficacy against the lethality of this snake species in two studies had been confirmed with the ED_50_ value of 10.95 μL and 14 μL [25, 43].

For the lethality of *Trimeresurus erythrurus* (red-tailed green pit viper) found in Myanmar, it can be cross-neutralized by anti-viper (Russell’s viper) from Myanmar Pharmaceutical Factory, Myanmar, resulting in the ED_50_ value of 125 μL [23], and green pit viper antivenin from QSMI, Thailand providing the ED_50_ value of 75 μL [23].

The lethality of *Trimeresurus insularis* (white-lipped island pit viper) found in Indonesia can also be cross-neutralized by green pit viper antivenin from QSMI, Thailand, with the ED_50_ value of 13.78 μL, ranging from 8.7–21.8 μL [31], and SABU polivalen (Bio Save) from PT Bio Farma (Persero), Indonesia resulting in the ED_50_ value of 145.9 μL, ranging from 129.12–164.97 μl [31].

#### Sea snakes and sea kraits of Asia

*Sea snakes of Asia*. Besides medically important venomous snakes, sea snakes in Asia, *Hydrophis* spp., were also tested against antivenoms. *Hydrophis schistosus* (beaked sea snake) found in Malaysia was cross-neutralized by the neurobivalent antivenom from the National Institute of Preventive Medicine, Taiwan, cobra antivenin, and the neuro-polyvalent snake antivenom from QSMI, Thailand, with ED_50_ values of 141.36 μL, 89.89 μL, and 100 μL, respectively [35]. Malaysian *H*. *schistosus* was also tested and cross-neutralized by the sea snake antivenom from CSL Ltd., Australia, with the ED_50_ value of 13.91 μL [49].

*Hydrophis curtus* (Shaw’s sea snake) found in Malaysia was also cross-neutralized by the neurobivalent antivenom from the National Institute of Preventive Medicine, Taiwan, cobra antivenin, and the neuro-polyvalent snake antivenom from QSMI, Thailand, with ED_50_ values of 200 μL, 89.89 μL, and 125 μL, respectively [35]. Malaysian *H*. *curtus* was also cross-neutralized by the sea snake antivenom from CSL Ltd., Australia, with the ED_50_ value of 9.87 μL, ranging from 7.98–12.21 μL [48].

Lastly, the lethality of *Hydrophis hardwickii* (spine-bellied sea snake) found in Japan was tested against cobra antivenin from QSMI, which showed ED_50_ values of 91.8 μL, 118.3 μL, and 128.5 μL [44].

*Sea krait of Asia*. Indonesian sea krait, *Laticauda colubrina* (yellow-lipped sea krait), was tested against the sea snake antivenom from CSL Ltd., Australia, and showed cross-neutralizing efficacy with the ED_50_ value of 8.84 μL, ranging from 6.76–11.54 μL [50].

### Quality assessment

The risk of bias from the included studies is assessed and presented in **S5 Table**. For selection bias, no information regarding allocation sequence generation and concealment was mentioned in any studies leading to the unclear risk of bias. At the same time, baseline characteristics were not reported in one study (2%), leading to the unclear risk of bias [44]. However, 51 studies stated baseline characteristics that caused no risk of bias in this domain. Regarding performance bias, random housing, caregiver blinding, and investigator blinding were not stated in the included studies leading to the unclear risk of bias. Random outcomes assessment and outcome assessor blinding were also not mentioned in any included preclinical studies resulting in the unclear risk of detection bias. Incomplete outcome data had not been addressed in all included studies, leading to the unclear risk of attrition bias. For reporting bias, no selective outcome reporting was reported in any included studies resulting in no risk of reporting bias. Lastly, all included studies were free of other problems that could result in a high risk of bias which caused no risk of other biases.

Assessment of reported data from 52 studies using reporting guidelines for *in vivo* neutralization of the lethality of antivenom assessment is performed and reported in **S6 Table**. In the experiment setup section, 47 (90%) studies had reported the antivenom batch numbers [21–37, 40–46, 49–56, 58–72]. Thirty-one (60%) studies reported the total protein concentration of antivenoms [22–33, 37, 40, 41, 48, 50, 52–56, 58–60, 64, 65, 67–69, 71]. Four (8%) studies provided no information regarding the origin of some snake venoms used in the studies [37, 40, 41, 72]. The ethical statement was not mentioned in six (13%) studies [43–47, 51].

In the animal section, the source of animals, mouse strains, and mouse weight were mentioned in 36, 49, and 49 studies, respectively. In contrast, husbandry information was not mentioned in any included studies.

In the procedure section, the numbers of LD_50_ used in the experiment, route of administration, and pre-incubation process were informed in all studies. Multiples of LD_50_ used in the experiment are between 2.5 to 8 folds which shows the details of each factor. For the route of administration, intravenous (90%) [21–37, 39–59, 61–67, 69, 72] and intraperitoneal (10%) [38, 60, 68, 70, 71] routes were used. The number of mice per group and experiment length were mentioned in 49 (94%) [21–39, 41–51, 53–60, 62–72] and 50 (96%) studies [21–42, 44, 46–72]. Nevertheless, the total number of mice used in each experiment was not provided in any studies.

In the reported results section, no group outcomes and adverse events were mentioned in any studies. Forty-nine (94%) studies mentioned a statistical method used in the ED_50_ calculation [21, 22, 24–37, 39–52, 54–72]. Probit analysis, a statistical method, was mainly used (79%) [22, 24–37, 40–42, 47–52, 54–67, 69, 71, 72]. The description of statistical analysis was reported in 28 (54%) studies. ED_50_ units were differently applied among the included studies. In most studies, 40 (77%) out of 52 used a microliter (μL) for the ED_50_ unit. However, units are varied among the rest of the studies, such as milligram per milliliter (mg/mL) (6%), microliter per gram (μg/g) (4%), milligram per gram (mg/g) (6%), milligram (mg) (4%), or milligram per kilogram (mg/kg) (2%).

### Situation of effective antivenom availability in each country in Asia

**Fig 2:**
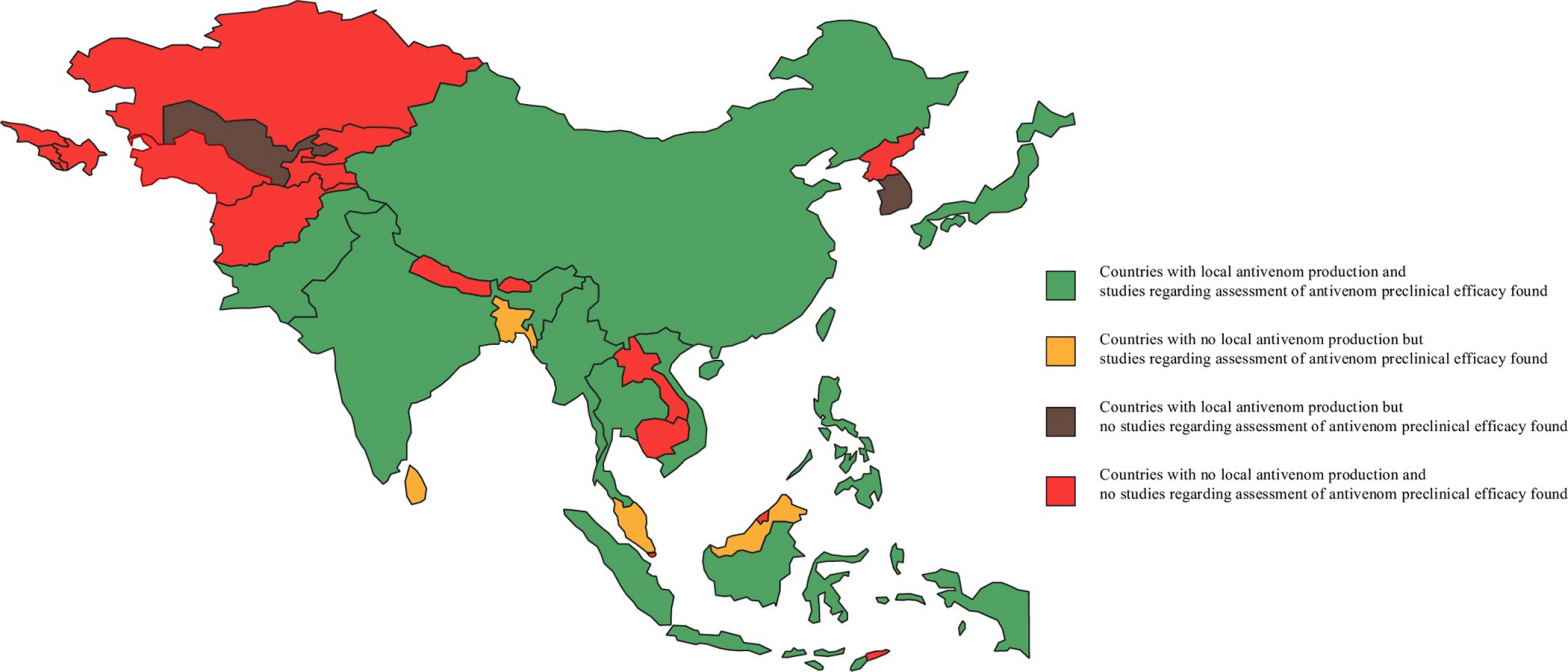
Heat map of local antivenom production availability and studies assessing preclinical efficacy of antivenoms. Made with Natural Earth. Free vector and raster map data @ naturalearthdata.com.

Summaries of the availability of local antivenom production and studies assessing the efficacy of antivenoms in each country in Asia are shown in **Fig 2**. There are 12 countries in Asia with local antivenom production. Among countries with no local antivenom production, the authors found only three countries where the efficacy of antivenoms had been assessed, namely Bangladesh, Sri Lanka, and Malaysia. In countries with local antivenom production, studies assessing antivenom efficacy were found in 10 countries except for South Korea and Uzbekistan.

To focus more on details on each country in Asia, **Table 2** demonstrates the availability of specific antivenoms against medically important venomous snakes in each country in Asia and shows Asian snakes with preclinical studies that confirmed antivenoms with cross-neutralizing or neutralizing ability against their lethality.

**Table 2:** Availability of specific antivenoms against categories one and two medically important venomous snakes in each country in Asia and available studies that confirmed antivenom cross-neutralizing or neutralizing ability against their lethality.

Region	Country	Category 1 Medically important venomous snakes (Category 1 MIVS)				Category 2 Medically important venomous snakes (Category 2 MIVS)			
		Total numbers of category 1 MIVS in each country	Total numbers of category 1 MIVS with specific antivenoms in each country	Total numbers of category 1 MIVS with antivenoms with confirmed neutralizing or cross-neutralizing ability	Total numbers of category 1 MIVS with no antivenoms available*	Total numbers of category 2 MIVS in each country	Total numbers of category 2 MIVS with specific antivenoms in each country	Total numbers of category 2 MIVS with antivenoms with confirmed neutralizing or cross-neutralizing ability	Total numbers of category 2 MIVS with no antivenoms available*
**East Asia**	China	7	3 (42.86%)	4 (57.14%)	3 (42.86%)	16	1 (6.25%)	2 (12.50%)	14 (87.50%)
	Hong Kong	3	0	0	3 (100%)	0	0	0	0
	Japan	1	1 (100%)	0	0	3	1 (33.33%)	0	2 (66.67%)
	North Korea	1	0	0	1 (100%)	3	0	0	3 (100%)
	South Korea	1	1 (100%)	0	0	3	1 (33.33%)	0	2 (66.67%)
	Taiwan	4	4 (100%)	2 (50%)	0	2	2 (100%)	2 (100%)	0
**South Asia**	Afghanistan	3	0	0	3 (100%)	5	0	0	5 (100%)
	Bangladesh	5	0	1 (20%)	4 (80%)	7	0	1 (14.29%)	6 (85.71%)
	Bhutan	2	0	0	2 (100%)	8	0	0	8 (100%)
	India	6	4 (66.67%)	5 (83.33%)	1 (16.67%)	18	0	2 (11.11%)	16 (88.89%)
	Nepal	5	0	0	5 (100%)	9	0	0	9 (100%)
	Pakistan	6	4 (66.67%)	4 (66.67%)	1 (16.67%)	3	0	0	3 (100%)
	Sri Lanka	4	0	4 (100%)	0	4	0	1 (25%)	3 (75%)
**Southeast Asia**	Brunei Darussalam	1	0	0	1 (100%)	7	0	0	7 (100%)
	Cambodia	6	0	0	6 (100%)	4	0	0	4 (100%)
	Indonesia	6	2 (33.83%)	5 (83.33%)	1 (16.67%)	9	1 (11.11%)	4 (44.44%)	5 (55.56%)
	Malaysia	4	0	4 (100%)	0	8	0	4 (50%)	4 (50%)
	Myanmar	7	2 (28.57%)	2 (28.57%)	4 (57.14%)	11	0	0	11 (100%)
	Philippines	3	1 (33.33%)	2 (66.67%)	1 (33.33%)	5	0	0	5 (100%)
	Singapore	2	0	0	2 (100%)	4	0	0	4 (100%)
	Thailand	6	5 (83.33%)	5 (83.33%)	0	8	2 (25%)	4 (50%)	4 (50%)
	Lao People’s Democratic Republic	6	0	0	6 (100%)	6	0	0	6 (100%)
	Timor-Leste	1	0	0	1 (100%)	1	0	0	1 (100%)
	Vietnam	8	1 (12.50%)	1 (12.50%)	7 (87.50%)	8	0	0	8 (100%)

*Neither specific antivenoms nor antivenoms with cross-neutralizing ability are available against them.

East Asia countries such as Japan, South Korea, and Taiwan have local specific antivenoms against all category one medically important venomous snakes in their countries. In comparison, Hong Kong and North Korea have no local specific antivenoms against category one medically important venomous snakes, and no studies have been found confirming antivenoms with cross-neutralizing or neutralizing ability. In addition, China has local specific antivenoms against only three (42.86%) category one medically important venomous snakes and one (6.25%) of category two medically important venomous snakes. However, some studies were found to confirm antivenom efficacy against medically important venomous snakes with no local specific antivenoms found.

India and Pakistan are the only two countries in South Asia that can produce local antivenoms. However, the availability of local antivenoms has covered category one medically important venomous snakes at 66.67% in their countries.

For other countries in South Asia without local antivenom production, it has been confirmed that there are antivenoms that can cross-neutralize against the lethality of category one medically important venomous snakes in their countries. For example, it was found that there are available antivenoms with cross-neutralizing ability against all (100%) category one medically important venomous snakes in Sri Lanka. No specific antivenom was developed against category two medically important venomous snakes in South Asia. However, studies confirming antivenom efficacy against them were conducted in Bangladesh, India, and Sri Lanka.

Among countries in Southeast Asia, Brunei Darussalam, Cambodia, Malaysia, and Lao PDR were the countries without local antivenom production. However, studies confirming antivenom cross-neutralizing ability were found for Malaysia’s four (100%) category one medically important venomous snakes. However, no studies in Brunei Darussalam, Cambodia, and Lao PDR confirmed antivenom efficacy.

Thailand produced local antivenoms against five (83%) out of seven in category one medically important venomous snakes, which was the highest among countries in Southeast Asia. For category two medically important venomous snakes, only Thailand and Indonesia can produce specific antivenoms against two and one category two medically important venomous snakes, respectively. Additionally, studies confirming efficacy against category two medically important venomous snakes were found only in Malaysia among the countries with no local antivenom production.

## Discussion

Snakebite envenoming is a neglected tropical disease with an issue of access to effective treatment. Therefore, imported antivenoms can be used as an alternative treatment in countries with no local antivenom production. Nonetheless, to confirm its effectiveness, antivenom efficacy must be assessed before use in designated areas. This is because snake venoms are different within and between species due to geographic variation, which can lead to varying strengths of antivenom-neutralizing ability [73].

As demonstrated in the results, neuro-polyvalent snake antivenom from QSMI, Thailand, can cross-neutralize against the lethality of many medically important venomous snakes with no specific antivenoms within the Elapidae family. For example, *N*. *atra* in Taiwan, *N*. *naja* in Sri Lanka, *B*. *candidus* in Indonesia (Java Island) and Malaysia, *N*. *kaouthia* in Malaysia, *N*. *sputatrix* in Malaysia, and *N*. *sumatrana* in Indonesia (Sumatra). Consistently, hemato-polyvalent snake antivenom from QSMI, Thailand, can cross-neutralize against the lethality of snakes in Asia without specific antivenoms in Viperidae family, which are *D*. *russelii* in Sri Lanka, *H*. *hypnale* in Sri Lanka, *C*. *rhodostoma* in Malaysia, and *D*. *siamensis* in Myanmar. It showed that polyvalent antivenoms may cross-neutralize against different snake species within a similar family and in other regions.

Regarding monovalent antivenoms, they can cross-neutralize against snakes with a similar genus from different areas. For instance, a cobra (*N*. *kaouthia*) antivenom from QSMI, Thailand, has a cross-neutralizing ability against the lethality of *N*. *kaouthia* in Malaysia and *N*. *siamensis* in Thailand. Furthermore, local monovalent antivenoms can cross-neutralize against the lethality of snakes with a similar genus in their countries, such as monovalent (*N*. *philippinensis*) cobra antivenin from Biologicals Manufacturing Division, Research Institute for Tropical Medicine, Malaysia, can cross-neutralize against the lethality of *N*. *samarensis* in the Philippines. However, no studies confirm neutralizing ability against lethality for more than 50% of medically important venomous snakes in Asia. This result showed a lack of information on this public health issue following a previous systematic review of antivenom preclinical efficacy in sub-Saharan Africa [18]. Surprisingly, a polyvalent snake antivenom from India, in which *N*. *naja* venom was included in the immunizing mixture in the development process, cannot neutralize the lethality of *N*. *naja* in Maharashtra, Southwest India. This finding supports that the quality of antivenoms is also an issue for this neglected tropical disease.

Regarding the risk of bias assessment, the limited reported data in the included studies caused an unclear risk of bias in most domains. Those data should be provided to help assess the risk of bias in the study design. Regarding the information reported in the included studies, some studies included in this systematic review did not report on snake origins. Reporting on the snake origin being tested in the experiment is crucial since there is geographical variation among snake venoms. Most studies did not report the total number of mice used in the experiment. This information can be used to evaluate the appropriate use of animals in which the regulatory requirement of 3Rs suggests that a minimal number of animals should be used, usually three to five groups of animals consisting of at least four per group [74]. For husbandry information, it is likely that detailed information, such as technical and customary to individual animal research facilities, is provided in the animal use protocols for ethics approval applied to the respective bodies. Therefore, ethical clearance should be provided in such studies involving the use of laboratory animals. However, out of the 52 studies analyzed, it was found that six (12%) did not present any ethical statement. Control groups were not directly displayed in some included studies, but it is compulsory and well-known to perform a test in control groups for *in vivo* neutralization studies without reporting them. The numbers of LD_50_ used in each experiment were different. Moreover, metrics of ED_50_ were used differently across the included studies, which limited the authors in a meta-analysis performed by a previous systematic review of antivenom preclinical efficacy in sub-Saharan Africa [18]. It shows that universal guidelines for antivenom efficacy assessment should be optimized and globally applied for the standardization of antivenom. According to the WHO guidelines [13], it is recommended that ED_50_ should be expressed in three units: mg of venom neutralized by mL of antivenom, μL of antivenom required to neutralize the challenge dose of venom used, and μL of antivenom required for 1 mg of venom neutralizing. For the description of statistical analysis, it was reported in some studies. However, it can be referred to the earlier studies or established methods that can also be found in the included studies.

Due to ethical issues, it was challenging to perform clinical trials to assess antivenom efficacy in human participants [75]. According to the previous systematic review by Abouyannis M, et al. [76], only 43 clinical trials of snake antivenoms were conducted worldwide in the past 60 years, while only 22 clinical trials were performed in Asia. Additionally, results reported in the clinical trials were heterogeneous. Some measured outcomes in clinical trials were not valid. Therefore, guidance for conducting and reporting outcomes in clinical trials for snakebite envenoming was essential [76]. In contrast, the WHO developed a valid and reliable guideline to conduct preclinical studies regarding the assessment of antivenom efficacy [13]. Outcomes reported in preclinical studies, such as ED_50_, were universal, as demonstrated in the results of this study that conducting method was based on the WHO guideline. This confirmed that preclinical studies should be performed to ensure the efficacy of antivenoms in case clinical trials are not applicable. However, preclinical studies may not reflect the real-world context in human envenoming. According to the included studies, mice were intravenously or intraperitoneally injected with a preincubated venom-antivenom mixture which does not reflect the real-world context. Moreover, adverse events cannot be assessed in this design of the preclinical studies, consistent with the results where the adverse events were not reported in any included studies. Therefore, real-world evidence of antivenom effectiveness and safety in humans should be encouraged for further development to fulfill the snakebite-information system in the clinical aspect.

According to the antivenom availability situation demonstrated in the results, each country has different problems. Countries with local antivenoms have specific antivenoms but do not cover all medically important venomous snakes in their countries. Few studies from countries with no local antivenom production assessed the efficacy of antivenoms. This may imply that snakebite envenoming and the issue of access to effective antivenoms were not prioritized in these countries. There should be collaboration in Asia on this public health issue, for instance, collective policy suggestions providing guidelines for countries either with or without local antivenom production and a target product profile development for snake antivenoms in Asia.

Moreover, a snakebite database is crucial and remains unavailable [12]. This study is the first step in snakebite-information system development, especially in Asia. Nevertheless, this information system should be continuously updated to enable users, such as healthcare professionals, to search for effective antivenom neutralized against the toxic effects of snake venoms. In addition, despite the availability of antivenoms, the affordability, accessibility, and acceptability of antivenoms are also issues in snakebite envenoming. Snakebite victims may be unable to afford antivenoms and other supportive treatments, even utilizing traditional medicine that could be ineffective [9]. Further investigations on these dimensions are required.

In summary, access to effective antivenoms is an issue for this neglected tropical disease. Improvement of access to effective antivenoms is the key to solving snakebite envenoming. In countries with no local antivenom production, antivenom with confirmed cross-neutralizing ability can be used as an alternative treatment where new antivenom development is limited. To confirm antivenom efficacy, clinical trials are limited due to ethical issues. Thus, non-clinical studies should be performed to ensure antivenom efficacy. In countries with local antivenom production, some antivenoms were ineffective against snake venoms in the immunizing mixture. The efficacy of these available antivenoms also requires assessment. Hence, regulatory guidelines in Asia should be developed, which could elucidate what antivenom should be developed, how to produce antivenoms with assured quality, and what parameters should be reported to assure the reliability and validity of methods and outcomes.

This review had several limitations. First, only available antivenoms were included, while the new-generation antivenoms awaiting approval from the Food and Drug Administration via cross-neutralizing and neutralizing ability assessment were not included. Second, studies on antivenoms’ cross-neutralizing and neutralizing abilities against toxic effects other than lethality were excluded because the neutralization of toxic effects other than lethality was supplementary preclinical assays [13]. However, other toxic effects from snake venoms such as neurotoxic and hemorrhagic activities can cause serious, often lifelong, morbidity and disability [77, 78]. Therefore, further investigations are encouraged to summarize the preclinical efficacy of antivenoms against these toxic effects. Third, only Asian snakes were included. These limitations necessitate the periodic update of this review. Fourth, preclinical studies are not required to be published for antivenom registration. Thus, in-house experiments could not be included in this review. Lastly, the comparison of antivenom efficacy by solely comparing the ED_50_ values is limited because the ED_50_ values are heterogenous and many variables affect the ED_50_ values such as different challenge lethal doses and routes of injection. The amount of venom injected per bite can also affect the efficacy of antivenoms, however, this parameter is not commonly reported in the included studies and it varies depending on the species, size, and geographical origins of snakes, as well as the number of fangs that penetrated the skin [11]. Further investigations are required where snakebite envenoming is an issue to develop more exhaustive databases to solve this neglected tropical disease and achieve the goal of halving the disability and mortality from snakebite envenoming according to the WHO’s roadmap [6].

## Conclusion

Cross-neutralizing ability against the lethality of Asian snake venom was confirmed. This strategy can help improve equal access to geographically effective antivenoms, improving the snakebite envenoming patient outcomes. It may also bypass the investment in new antivenom development, especially in countries without local antivenom production. This study provides data for a snakebite-information system, which is still lacking. Nevertheless, studies confirming antivenom effectiveness against the lethality of some medically important venomous snakes are unavailable. Thus, the development of more databases should be encouraged.

## Supporting information

S1 Table PRISMA checklist. (DOCX)

S2 Table Search strategies of each database from inception to May 30^th^ 2022. (DOCX)

S3 Table Study characteristics. (DOCX)

S4 Table Results of antivenom neutralization against snake venom lethality from the included preclinical studies. (DOCX)

S5 Table Risk of bias assessment using Systematic Review Centre for Laboratory animal Experimentation’s (SYRCLE) risk of bias tool for animal studies. (DOCX)

S6 Table Assessment of reporting guideline for in vivo neutralization of lethality of antivenom assessment. (DOCX)

S7 Table Excluded studies with reasons from the search in this review. (DOCX)
