# Supplementary material for: Antivenom preclinical efficacy testing against Asian snakes and their availability in Asia: A systematic review
Source: PLoS One. 2023 Jul 19;18(7):e0288723. doi: 10.1371/journal.pone.0288723 (PMC10355433; doi:10.1371/journal.pone.0288723)
Supplement: S2 Table — (DOCX) [file pone.0288723.s002.docx]

# **S2 Table. Search strategies of each database from inception to May 30th, 2022.**

| **Database** | **Search Term** | **Results** |
| --- | --- | --- |
| PubMed | ((Antivenom OR Antivenin OR Antivenene OR Anti-venom) AND Snake* AND Neutrali*) | 1,001 |
| Web of Science | TS = (Antivenins OR Antivenom OR Antivenin OR Antivenene OR Anti-venom) AND TS=Snake* AND TS=neutrali* | 967 |
| Scopus | (Antivenom OR Antivenin OR Antivenene OR Anti-venom) AND Snake AND Neutralize. | 1,168 |
| Embase | (‘snake venom antiserum’/exp/mj OR antivenom OR antivenin OR amtivenene OR ‘anti venom’) AND snake* | 1,148 |
