# Supplementary material for: Antivenom preclinical efficacy testing against Asian snakes and their availability in Asia: A systematic review
Source: PLoS One. 2023 Jul 19;18(7):e0288723. doi: 10.1371/journal.pone.0288723 (PMC10355433; doi:10.1371/journal.pone.0288723)
Supplement: S3 Table — (DOCX) [file pone.0288723.s003.docx]

# **S3 Table. Study characteristics.**

| **Author, year** | **Family** | **Snake species** | **Geographic origin of snakes** | **Antivenom** | **Snake venoms included in the immunization mixture** | **Geographic origin of included snake venoms** | **Manufacturer** | **Batch number** | **Expiry date** | **Protein concentration (mg/mL)** |
| --- | --- | --- | --- | --- | --- | --- | --- | --- | --- | --- |
| Tan KY, 2022 (1) | Viperidae | *Deinagkistrodon acutus* | Taiwan | Deinagkistrodon acutus Monovalent Antivenoms | *Deinagkistrodon acutus* | Taiwan | Central for Disease Control, Taiwan | FR10301 | Not reported | Not reported |
| Tan KY, 2022 (1) | Viperidae | *Deinagkistrodon acutus* | China | Deinagkistrodon acutus Monovalent Antivenoms | *Deinagkistrodon acutus* | Taiwan | Central for Disease Control, Taiwan | FR10301 | Not reported | Not reported |
| Tan KY, 2022 (1) | Viperidae | *Deinagkistrodon acutus* | Taiwan | Deinagkistrodon acutus Monovalent Antivenoms | *Deinagkistrodon acutus* | China | Shanghai Serum Bio-Technology Co., Ltd., China | 20140501 | Not reported | Not reported |
| Tan KY, 2022 (1) | Viperidae | *Deinagkistrodon acutus* | China | Deinagkistrodon acutus Monovalent Antivenoms | *Deinagkistrodon acutus* | China | Shanghai Serum Bio-Technology Co., Ltd, China | 20140501 | Not reported | Not reported |
| Chanhome O, 2022 (2) | Viperidae | *Protobothrops kelomohy* | Thailand  (Northern Thailand) | Haemato-polyvalent snake antivenom | *Calloselasma rhodostoma, Trimeresurus albolabris, Daboia siamensis* | Thailand | Queen Saovabha Memorial Institute, Thailand | HP00118 | Not reported | Not reported |
| Chanhome O, 2022 (2) | Viperidae | *Protobothrops kelomohy* | Thailand  (Northern Thailand) | Russell's viper antivenin | *Daboia siamensis* | Thailand | Queen Saovabha Memorial Institute, Thailand | WR00117 | Not reported | Not reported |
| Chanhome O, 2022 (2) | Viperidae | *Protobothrops kelomohy* | Thailand  (Northern Thailand) | Green pit viper antivenin | *Trimeresurus albolabris* | Thailand | Queen Saovabha Memorial Institute, Thailand | TA00119 | Not reported | Not reported |
| Chanhome O, 2022 (2) | Viperidae | *Protobothrops kelomohy* | Thailand  (Northern Thailand) | Malayan pit viper antivenin | *Calloselasma rhodostoma* | Thailand | Queen Saovabha Memorial Institute, Thailand | CR00218 | Not reported | Not reported |
| Wong KY, 2021 (3) | Elapidae | *Naja naja* | Sri Lanka (Colombo) | Snake Venom Antiserum I.P. (Asia) | *Bungarus caeruleus, Daboia russelii, Echis carinatus, Naja naja* | India | VINS Bioproducts Ltd., India | 01AS12041 | Not reported | Not reported |
| Faisal T, 2021 (4) | Viperidae | *Daboia russelii* | Sri Lanka | Snake Venom Antiserum I.P. (Asia) | *Bungarus caeruleus, Daboia russelii, Echis carinatus, Naja naja* | India | VINS Bioproducts Ltd., India | 01AS12041 | Not reported | 84.93 ± 4.3 |
| Faisal T, 2021 (4) | Viperidae | *Daboia russelii* | India | Snake Venom Antiserum I.P. (Asia) | *Bungarus caeruleus, Daboia russelii, Echis carinatus, Naja naja* | India | VINS Bioproducts Ltd., India | 01AS12041 | Not reported | 84.93 ± 4.3 |
| Attarde S, 2021 (5) | Elapidae | *Naja sagittifera* | India  (Andaman Island) | Polyvalent snake antivenom (Asia) | *Bungarus caeruleus, Daboia russelii, Echis carinatus, Naja naja* | India | Premium Serums & Vaccines Pvt. Ltd., India | ASVS-I Lyo.013 | Not reported | 26.2 ± 1.2 |
| Attarde S, 2021 (5) | Elapidae | *Naja sagittifera* | India  (Andaman Island) | Polyvalent Snake Antivenom (Asia) | *Bungarus caeruleus, Daboia russelii, Echis carinatus, Naja naja* | India | Bharat Serums & Vaccines, India | A05318087 | Not reported | 26.5 ± 0.77 |
| Attarde S, 2021 (5) | Elapidae | *Naja sagittifera* | Andaman Island, India | Cobra antivenin | *Naja kaouthia* | Thailand | Queen Saovabha Memorial Institute, Thailand | NK00112 | Not reported | 25.9 ± 0.44 |
| Attarde S, 2021 (5) | Elapidae | *Naja naja* | India | Polyvalent snake antivenom (Asia) | *Bungarus caeruleus, Daboia russelii, Echis carinatus, Naja naja* | India | Premium Serums & Vaccines Pvt. Ltd., India | ASVS-I Lyo.013 | Not reported | 26.2 ± 1.2 |
| Attarde S, 2021 (5) | Elapidae | *Naja naja* | India | Polyvalent Snake Antivenom (Asia) | *Bungarus caeruleus, Daboia russelii, Echis carinatus, Naja naja* | India | Bharat Serums & Vaccines, India | A05318087 | Not reported | 26.5 ± 0.77 |
| Tan CH, 2021 (6) | Elapidae | *Naja philippinensis* | Philippines | Philippine Cobra antivenom | *Naja philippinensis* | Philippines | Research Institute for Tropical Medicine in the Philippines, Philippines | 201804 | Apr 2021 | 16.2 ± 1.1 |
| Tan CH, 2021 (6) | Elapidae | *Naja samarensis* | Philippines | Philippine Cobra antivenom | *Naja philippinensis* | Philippines | Research Institute for Tropical Medicine in the Philippines, Philippines | 201804 | Apr 2021 | 16.2 ± 1.1 |
| Oh AMF, 2021 (7) | Elapidae | *Bungarus multicinctus* | China | Bungarus multicinctus monovalent antivenom | *Bungarus multicinctus* | China | Shanghai Serum Biological Technology Co., Ltd., China | 20150101 | Jan 2018 | 77 |
| Oh AMF, 2021 (7) | Elapidae | *Bungarus multicinctus* | Taiwan | Bungarus multicinctus monovalent antivenom | *Bungarus multicinctus* | China | Shanghai Serum Biological Technology Co., Ltd., China | 20150101 | Jan 2018 | 77 |
| Oh AMF, 2021 (7) | Elapidae | *Bungarus multicinctus* | China | Neuro bivalent antivenom | *Bungarus multicinctus, Naja atra* | Taiwan | Centres for Disease Control, Taiwan | FN10101 | Apr 2017 | 27 |
| Oh AMF, 2021 (7) | Elapidae | *Bungarus multicinctus* | Taiwan | Neuro bivalent antivenom | *Bungarus multicinctus, Naja atra* | Taiwan | Centres for Disease Control, Taiwan | FN10101 | Apr 2017 | 27 |
| Laxme RRS, 2021 (8) | Viperidae | *Daboia russelii* | India (Punjab, North India) | Polyvalent snake antivenom (Asia) | *Bungarus caeruleus, Daboia russelii, Echis carinatus, Naja naja* | India | Premium Serums & Vaccines Pvt. Ltd., India | ASVS(I)-Lyo013 | Nov 2022 | 26.2 ± 1.2 |
| Laxme RRS, 2021 (8) | Viperidae | *Daboia russelii* | India (Andhra Pradesh, Southeast India) | Polyvalent snake antivenom (Asia) | *Bungarus caeruleus, Daboia russelii, Echis carinatus, Naja naja* | India | Premium Serums & Vaccines Pvt. Ltd., India | ASVS(I)-Lyo013 | Nov 2022 | 26.2 ± 1.2 |
| Laxme RRS, 2021 (8) | Viperidae | *Daboia russelii* | India (Wast Bengal, East India) | Polyvalent snake antivenom (Asia) | *Bungarus caeruleus, Daboia russelii, Echis carinatus, Naja naja* | India | Premium Serums & Vaccines Pvt. Ltd., India | ASVS(I)-Lyo013 | Nov 2022 | 26.2 ± 1.2 |
| Laxme RRS, 2021 (8) | Viperidae | *Daboia russelii* | India (Maharashtra, Southwest India) | Polyvalent snake antivenom (Asia) | *Bungarus caeruleus, Daboia russelii, Echis carinatus, Naja naja* | India | Premium Serums & Vaccines Pvt. Ltd., India | ASVS(I)-Lyo013 | Nov 2022 | 26.2 ± 1.2 |
| Laxme RRS, 2021 (8) | Viperidae | *Daboia russelii* | India (Madhya Pradesh, Central India) | Polyvalent snake antivenom (Asia) | *Bungarus caeruleus, Daboia russelii, Echis carinatus, Naja naja* | India | Premium Serums & Vaccines Pvt. Ltd., India | ASVS(I)-Lyo013 | Nov 2022 | 26.2 ± 1.2 |
| Laxme RRS, 2021 (9) | Elapidae | *Naja naja* | India (Punjab, North India) | Polyvalent snake antivenom (Asia) | *Bungarus caeruleus, Daboia russelii, Echis carinatus, Naja naja* | India | Premium Serums & Vaccines Pvt. Ltd., India | ASVS(I)-Lyo013 | Nov 2022 | 26.2 ± 1.2 |
| Laxme RRS, 2021 (9) | Elapidae | *Naja naja* | India (Andhra Pradesh, Southeast India) | Polyvalent snake antivenom (Asia) | *Bungarus caeruleus, Daboia russelii, Echis carinatus, Naja naja* | India | Premium Serums & Vaccines Pvt. Ltd., India | ASVS(I)-Lyo013 | Nov 2022 | 26.2 ± 1.2 |
| Laxme RRS, 2021 (9) | Elapidae | *Naja naja* | India (Wast Bengal, East India) | Polyvalent snake antivenom (Asia) | *Bungarus caeruleus, Daboia russelii, Echis carinatus, Naja naja* | India | Premium Serums & Vaccines Pvt. Ltd., India | ASVS(I)-Lyo013 | Nov 2022 | 26.2 ± 1.2 |
| Laxme RRS, 2021 (9) | Elapidae | *Naja naja* | India (Maharashtra, Southwest India) | Polyvalent snake antivenom (Asia) | *Bungarus caeruleus, Daboia russelii, Echis carinatus, Naja naja* | India | Premium Serums & Vaccines Pvt. Ltd., India | ASVS(I)-Lyo013 | Nov 2022 | 26.2 ± 1.2 |
| Laxme RRS, 2021 (9) | Elapidae | *Naja naja* | India (Madhya Pradesh, Central India) | Polyvalent snake antivenom (Asia) | *Bungarus caeruleus, Daboia russelii, Echis carinatus, Naja naja* | India | Premium Serums & Vaccines Pvt. Ltd., India | ASVS(I)-Lyo013 | Nov 2022 | 26.2 ± 1.2 |
| Yee KT, 2020 (10) | Viperidae | *Trimeresurus erythrurus* | Myanmar | Green pit viper antivenin | *Trimeresurus albolabris* | Thailand | Queen Saovabha Memorial Institute, Thailand | TA00317 | Not reported | Not reported |
| Yee KT, 2020 (10) | Viperidae | *Trimeresurus erythrurus* | Myanmar | Russell’ viper anti-venom | *Daboia siamensis* | Myanmar | Myanmar Pharmaceutical Factory, Myanmar | DJ.1704 | May 2023 | Not reported |
| Tan KY, 2020 (11) | Elapidae | *Ophiophagus hannah* | Malaysia (Seremban) | King cobra antivenin | *Ophiophagus hannah* | Thailand | Queen Saovabha Memorial Institute, Thailand | LH00116 | 7 Nov 2021 | 19.7 ± 0.7 |
| Tan KY, 2020 (11) | Elapidae | *Ophiophagus hannah* | Thailand (Bangkok) | King cobra antivenin | *Ophiophagus hannah* | Thailand | Queen Saovabha Memorial Institute, Thailand | LH00116 | 7 Nov 2021 | 19.7 ± 0.7 |
| Tan KY, 2020 (11) | Elapidae | *Ophiophagus hannah* | China (Guangzhou) | King cobra antivenin | *Ophiophagus hannah* | Thailand | Queen Saovabha Memorial Institute, Thailand | LH00116 | 7 Nov 2021 | 19.7 ± 0.7 |
| Tan KY, 2020 (11) | Elapidae | *Ophiophagus hannah* | Indonesia (East Java Island) | King cobra antivenin | *Ophiophagus hannah* | Thailand | Queen Saovabha Memorial Institute, Thailand | LH00116 | 7 Nov 2021 | 19.7 ± 0.7 |
| Tan KY, 2020 (11) | Elapidae | *Ophiophagus hannah* | Malaysia (Seremban) | Serum Anti Bisa Ular (Biosave) | *Naja sputatrix, Calloselasma rhodostoma, Bungarus fasciatus* | Indonesia | Bio Farma, Indonesia | 4701516 | Aug 2018 | 102.9 ± 0.8 |
| Tan KY, 2020 (11) | Elapidae | *Ophiophagus hannah* | Thailand (Bangkok) | Serum Anti Bisa Ular (Biosave) | *Naja sputatrix, Calloselasma rhodostoma, Bungarus fasciatus* | Indonesia | Bio Farma, Indonesia | 4701516 | Aug 2018 | 102.9 ± 0.8 |
| Tan KY, 2020 (11) | Elapidae | *Ophiophagus hannah* | China (Guangzhou) | Serum Anti Bisa Ular (Biosave) | *Naja sputatrix, Calloselasma rhodostoma, Bungarus fasciatus* | Indonesia | Bio Farma, Indonesia | 4701516 | Aug 2018 | 102.9 ± 0.8 |
| Tan KY, 2020 (11) | Elapidae | *Ophiophagus hannah* | Indonesia (East Java Island) | Serum Anti Bisa Ular (Biosave) | *Naja sputatrix, Calloselasma rhodostoma, Bungarus fasciatus* | Indonesia | Bio Farma, Indonesia | 4701516 | Aug 2018 | 102.9 ± 0.8 |
| Tan KY, 2020 (11) | Elapidae | *Ophiophagus hannah* | Malaysia (Seremban) | Naja atra antivenom | *Naja atra* | China | Shanghai Institute Biological Technology Co., Ltd., China | 20140501 | May 2017 | 255.6 ± 3.2 |
| Tan KY, 2020 (11) | Elapidae | *Ophiophagus hannah* | Thailand (Bangkok) | Naja atra antivenom | *Naja atra* | China | Shanghai Institute Biological Technology Co., Ltd., China | 20140501 | May 2017 | 255.6 ± 3.2 |
| Tan KY, 2020 (11) | Elapidae | *Ophiophagus hannah* | China (Guangzhou) | Naja atra antivenom | *Naja atra* | China | Shanghai Institute Biological Technology Co., Ltd., China | 20140501 | May 2017 | 255.6 ± 3.2 |
| Tan KY, 2020 (11) | Elapidae | *Ophiophagus hannah* | Indonesia (East Java Island) | Naja atra antivenom | *Naja atra* | China | Shanghai Institute Biological Technology Co., Ltd., China | 20140501 | May 2017 | 255.6 ± 3.2 |
| Lin B, 2020 (12) | Elapidae | *Bungarus multicinctus* | China | Bungarus multicinctus monovalent antivenom | *Bungarus multicinctus* | China | Shanghai Serum Biological Technology Co., Ltd., China | S10820179 | 16 Mar 2020 | Not reported |
| Lin B, 2020 (12) | Elapidae | *Bungarus fasciatus* | China | Bungarus multicinctus monovalent antivenom | *Bungarus multicinctus* | China | Shanghai Serum Biological Technology Co., Ltd., China | S10820179 | 16 Mar 2020 | Not reported |
| Lin B, 2020 (12) | Elapidae | *Naja atra* | China | Bungarus multicinctus monovalent antivenom | *Bungarus multicinctus* | China | Shanghai Serum Biological Technology Co., Ltd., China | S10820179 | 16 Mar 2020 | Not reported |
| Lin B, 2020 (12) | Elapidae | *Ophiophagus hannah* | China | Bungarus multicinctus monovalent antivenom | *Bungarus multicinctus* | China | Shanghai Serum Biological Technology Co., Ltd., China | S10820179 | 16 Mar 2020 | Not reported |
| Liew JL, 2020 (13) | Viperidae | *Trimeresurus purpureomaculatus* | Malaysia | Green pit viper antivenin | *Trimeresurus albolabris* | Thailand | Queen Saovabha Memorial Institute, Thailand | TA00213 | Not reported | 20.2 ± 0.7 |
| Liew JL, 2020 (13) | Viperidae | *Trimeresurus albolabris* | Thailand | Green pit viper antivenin | *Trimeresurus albolabris* | Thailand | Queen Saovabha Memorial Institute, Thailand | TA00213 | Not reported | 20.2 ± 0.7 |
| Lee LP, 2020 (14) | Viperidae | *Trimeresurus wiroti* | Malaysia | Green pit viper antivenin | *Trimeresurus albolabris* | Thailand | Queen Saovabha Memorial Institute, Thailand | 00116 | Feb 2021 | 18.07 ± 1.1 |
| Lee LP, 2020 (14) | Viperidae | *Trimeresurus puniceus* | Indonesia | Green pit viper antivenin | *Trimeresurus albolabris* | Thailand | Queen Saovabha Memorial Institute, Thailand | 00116 | Feb 2021 | 18.07 ± 1.1 |
| Hia YL, 2020 (15) | Elapidae | *Bungarus fasciatus* | Malaysia (Peninsular) | Banded krait antivenin | *Bungarus fasciatus* | Thailand | Queen Saovabha Memorial Institute, Thailand | BK00114 | Dec 2019 | 58.73 ± 0.02 |
| Hia YL, 2020 (15) | Elapidae | *Bungarus fasciatus* | Thailand (Bangkok) | Banded krait antivenin | *Bungarus fasciatus* | Thailand | Queen Saovabha Memorial Institute, Thailand | BK00114 | Dec 2019 | 58.73 ± 0.02 |
| Hia YL, 2020 (15) | Elapidae | *Bungarus fasciatus* | Indonesia (Java Island) | Banded krait antivenin | *Bungarus fasciatus* | Thailand | Queen Saovabha Memorial Institute, Thailand | BK00114 | Dec 2019 | 58.73 ± 0.02 |
| Hia YL, 2020 (15) | Elapidae | *Bungarus fasciatus* | Myanmar | Banded krait antivenin | *Bungarus fasciatus* | Thailand | Queen Saovabha Memorial Institute, Thailand | BK00114 | Dec 2019 | 58.73 ± 0.02 |
| Hia YL, 2020 (15) | Elapidae | *Bungarus fasciatus* | China (Guangdong) | Banded krait antivenin | *Bungarus fasciatus* | Thailand | Queen Saovabha Memorial Institute, Thailand | BK00114 | Dec 2019 | 58.73 ± 0.02 |
| Choraria A, 2020 (16) | Viperidae | *Daboia russelii* | India | Polyvalent Snake Antivenom (Asia) | *Bungarus caeruleus, Daboia russelii, Echis carinatus, Naja naja* | India | Bharat Serums & Vaccines, India | A05317069 | Apr 2021 | 30 |
| Choraria A, 2020 (16) | Viperidae | *Echis carinatus* | India | Polyvalent Snake Antivenom (Asia) | *Bungarus caeruleus, Daboia russelii, Echis carinatus, Naja naja* | India | Bharat Serums & Vaccines, India | A05317069 | Apr 2021 | 30 |
| Tan CH, 2019 (17) | Viperidae | *Trimeresurus nebularis* | Malaysia | Green pit viper antivenin | *Trimeresurus albolabris* | Thailand | Queen Saovabha Memorial Institute, Thailand | TA00812 | Not reported | 20.2 |
| Pla D, 2019 (18) | Viperidae | *Daboia russelii* | Sri Lanka | Snake Venom Antiserum I.P. (Asia) | *Bungarus caeruleus, Daboia russelii, Echis carinatus, Naja naja* | India | VINS Bioproducts Ltd., India | Not reported | Jul 2016 | Not reported |
| Pla D, 2019 (18) | Viperidae | *Daboia russelii* | Sri Lanka | Polyvalent snake antivenom (Asia) | *Bungarus caeruleus, Daboia russelii, Echis carinatus, Naja naja* | India | Premium Serums & Vaccines Pvt Ltd., India | Not reported | Feb 2021 | Not reported |
| Pla D, 2019 (18) | Viperidae | *Daboia russelii* | Pakistan | Snake Venom Antiserum I.P. (Asia) | *Bungarus caeruleus, Daboia russelii, Echis carinatus, Naja naja* | India | VINS Bioproducts Ltd., India | Not reported | Jul 2016 | Not reported |
| Pla D, 2019 (18) | Viperidae | *Daboia russelii* | Pakistan | Polyvalent snake antivenom (Asia) | *Bungarus caeruleus, Daboia russelii, Echis carinatus, Naja naja* | India | Premium Serums & Vaccines Pvt Ltd., India | Not reported | Feb 2021 | Not reported |
| Pla D, 2019 (18) | Viperidae | *Daboia russelii* | Bangladesh | Snake Venom Antiserum I.P. (Asia) | *Bungarus caeruleus, Daboia russelii, Echis carinatus, Naja naja* | India | VINS Bioproducts Ltd., India | Not reported | Jul 2016 | Not reported |
| Pla D, 2019 (18) | Viperidae | *Daboia russelii* | Bangladesh | Polyvalent snake antivenom (Asia) | *Bungarus caeruleus, Daboia russelii, Echis carinatus, Naja naja* | India | Premium Serums & Vaccines Pvt Ltd., India | Not reported | Feb 2021 | Not reported |
| Oh AMF, 2019 (19) | Elapidae | *Bungarus sindanus* | Pakistan | Snake Venom Antiserum I.P. (Asia) | *Bungarus caeruleus, Daboia russelii, Echis carinatus, Naja naja* | India | VINS Bioproducts Ltd., India | 01AS12041 | Mar 2016 | 82 |
| Lingam TMC, 2019 (20) | Viperidae | *Daboia siamensis* | Thailand | Russell's viper antivenin | *Daboia siamensis* | Thailand | Queen Saovabha Memorial Institute, Thailand | WR00212 | Nov 2017 | 40.5 ± 0.6 |
| Lingam TMC, 2019 (20) | Viperidae | *Daboia siamensis* | Indonesia | Russell's viper antivenin | *Daboia siamensis* | Thailand | Queen Saovabha Memorial Institute, Thailand | WR00212 | Nov 2017 | 40.5 ± 0.6 |
| Lingam TMC, 2019 (20) | Viperidae | *Daboia siamensis* | Thailand | Serum Anti Bisa Ular (Biosave) | *Naja sputatrix, Calloselasma rhodostoma, Bungarus fasciatus* | Indonesia | Bio Farma, Indonesia | 4701516 | Aug 2018 | 123.1 ± 0.2 |
| Lingam TMC, 2019 (20) | Viperidae | *Daboia siamensis* | Indonesia | Serum Anti Bisa Ular (Biosave) | *Naja sputatrix, Calloselasma rhodostoma, Bungarus fasciatus* | Indonesia | Bio Farma, Indonesia | 4701516 | Aug 2018 | 123.1 ± 0.2 |
| Laxme RRS, 2019 (21) | Elapidae | *Naja naja* | India (Maharashtra, West India) | Polyvalent snake antivenom (Asia) | *Bungarus caeruleus, Daboia russelii, Echis carinatus, Naja naja* | India | Premium Serums & Vaccines Pvt. Ltd., India | 212013 | Not reported | 8.16 ± 0.15 |
| Laxme RRS, 2019 (21) | Elapidae | *Naja kaouthia* | India (Arunachal Pradesh, Northeast India) | Polyvalent snake antivenom (Asia) | *Bungarus caeruleus, Daboia russelii, Echis carinatus, Naja naja* | India | Premium Serums & Vaccines Pvt. Ltd., India | 212013 | Not reported | 8.16 ± 0.15 |
| Laxme RRS, 2019 (21) | Elapidae | *Naja kaouthia* | India (West Bengal, East India) | Polyvalent snake antivenom (Asia) | *Bungarus caeruleus, Daboia russelii, Echis carinatus, Naja naja* | India | Premium Serums & Vaccines Pvt. Ltd., India | 212013 | Not reported | 8.16 ± 0.15 |
| Laxme RRS, 2019 (21) | Elapidae | *Bungarus caeruleus* | India (Punjab, North India) | Polyvalent snake antivenom (Asia) | *Bungarus caeruleus, Daboia russelii, Echis carinatus, Naja naja* | India | Premium Serums & Vaccines Pvt. Ltd., India | 212013 | Not reported | 8.16 ± 0.15 |
| Laxme RRS, 2019 (21) | Elapidae | *Bungarus sindanus* | India (Rajasthan, Northwest India) | Polyvalent snake antivenom (Asia) | *Bungarus caeruleus, Daboia russelii, Echis carinatus, Naja naja* | India | Premium Serums & Vaccines Pvt. Ltd., India | 212013 | Not reported | 8.16 ± 0.15 |
| Laxme RRS, 2019 (21) | Elapidae | *Bungarus fasciatus* | India (West Bengal, East India) | Polyvalent snake antivenom (Asia) | *Bungarus caeruleus, Daboia russelii, Echis carinatus, Naja naja* | India | Premium Serums & Vaccines Pvt. Ltd., India | 212013 | Not reported | 8.16 ± 0.15 |
| Laxme RRS, 2019 (21) | Viperidae | *Echis carinatus* | India (Maharashtra, West India) | Polyvalent snake antivenom (Asia) | *Bungarus caeruleus, Daboia russelii, Echis carinatus, Naja naja* | India | Premium Serums & Vaccines Pvt. Ltd., India | 212013 | Not reported | 8.16 ± 0.15 |
| Laxme RRS, 2019 (21) | Viperidae | *Echis carinatus sochureki* | India (Rajasthan, Northwest India) | Polyvalent snake antivenom (Asia) | *Bungarus caeruleus, Daboia russelii, Echis carinatus, Naja naja* | India | Premium Serums & Vaccines Pvt. Ltd., India | 212013 | Not reported | 8.16 ± 0.15 |
| Deka A, 2019 (22) | Elapidae | *Naja kaouthia* | India | Polyvalent Snake Antivenom (Asia) | *Bungarus caeruleus, Daboia russelii, Echis carinatus, Naja naja* | India | Bharat Serums & Vaccines, India | A05315011 | Dec 2018 | Not reported |
| Deka A, 2019 (22) | Elapidae | *Naja kaouthia* | India | Snake Venom Antiserum I.P. (Asia) | *Bungarus caeruleus, Daboia russelii, Echis carinatus, Naja naja* | India | VINS Bioproducts Ltd., India | 01AS15007 | Jan 2019 | Not reported |
| Deka A, 2019 (22) | Elapidae | *Naja kaouthia* | India | Snake antivenin I.P. (Asia) | *Bungarus caeruleus, Daboia russelii, Echis carinatus, Naja naja* | India | Haffkine Biopharmaceutical Corporation Limited, India | AS111109 | Apr 2016 | Not reported |
| Deka A, 2019 (22) | Elapidae | *Naja kaouthia* | Bangladesh | Polyvalent Snake Antivenom (Asia) | *Bungarus caeruleus, Daboia russelii, Echis carinatus, Naja naja* | India | Bharat Serums & Vaccines, India | A05315011 | Dec 2018 | Not reported |
| Deka A, 2019 (22) | Elapidae | *Naja kaouthia* | Bangladesh | Snake Venom Antiserum I.P. (Asia) | *Bungarus caeruleus, Daboia russelii, Echis carinatus, Naja naja* | India | VINS Bioproducts Ltd., India | 01AS15007 | Jan 2019 | Not reported |
| Deka A, 2019 (22) | Elapidae | *Naja kaouthia* | Bangladesh | Snake antivenin I.P. (Asia) | *Bungarus caeruleus, Daboia russelii, Echis carinatus, Naja naja* | India | Haffkine Biopharmaceutical Corporation Limited, India | AS111109 | Apr 2016 | Not reported |
| Chaisakul J, 2019 (23) | Viperidae | *Daboia siamensis* | Thailand | Russell's viper antivenin | *Daboia siamensis* | Thailand | Queen Saovabha Memorial Institute, Thailand | WR00117 | Nov 2022 | Not reported |
| Chaisakul J, 2019 (23) | Viperidae | *Daboia siamensis* | Myanmar | Russell's viper antivenin | *Daboia siamensis* | Thailand | Queen Saovabha Memorial Institute, Thailand | WR00117 | Nov 2022 | Not reported |
| Chaisakul J, 2019 (23) | Viperidae | *Daboia siamensis* | Taiwan | Russell's viper antivenin | *Daboia siamensis* | Thailand | Queen Saovabha Memorial Institute, Thailand | WR00117 | Nov 2022 | Not reported |
| Chaisakul J, 2019 (23) | Viperidae | *Daboia siamensis* | China | Russell's viper antivenin | *Daboia siamensis* | Thailand | Queen Saovabha Memorial Institute, Thailand | WR00117 | Nov 2022 | Not reported |
| Tan CH, 2018 (24) | Elapidae | *Hydrophis curtus* | Malaysia | Sea Snake Antivenom | *Enhydrina schistosa* | Australia | CSL Ltd., Australia | Not reported | Not reported | 217.2 ± 3 |
| Tan KY, 2018 (25) | Viperidae | *Daboia siamensis* | China (Guangxi) | Doboia siamensis Monovalent Snake Antivenom | *Doboia siamensis* | Taiwan | Centres for Disease Control, Taiwan | FR10301 | 31 Oct 2019 | 19.3 ± 0.5 |
| Tan KY, 2018 (25) | Viperidae | *Daboia siamensis* | Taiwan | Doboia siamensis Monovalent Snake Antivenom | *Doboia siamensis* | Taiwan | Centres for Disease Control, Taiwan | FR10301 | 31 Oct 2019 | 19.3 ± 0.5 |
| Tan KY, 2018 (25) | Viperidae | *Daboia siamensis* | China (Guangxi) | Gloydius brevicaudus Monovalent Snake Antivenom | *Gloydius brevicaudus* | China | Shanghai Serum Biological Technology Co., Ltd., China | 20141001 | 30 Oct 2017 | 168.5 ± 0.7 |
| Tan KY, 2018 (25) | Viperidae | *Daboia siamensis* | China (Guangxi) | Deinagkistrodon acutus Monovalent Snake Antivenom | *Deinagkistrodon acutus* | China | Shanghai Serum Biological Technology Co., Ltd., China | 20140501 | 26 May 2017 | 181.1 ± 6.4 |
| Sanz L, 2018 (26) | Viperidae | *Daboia siamensis* | Taiwan | Doboia siamensis Monovalent Snake Antivenom | *Doboia siamensis* | Taiwan | Centres for Disease Control, Taiwan | Various batches | 30 Oct 2019 | 1310 U/vial |
| Liu BS, 2018 (27) | Elapidae | *Naja atra* | Taiwan | Neuro bivalent antivenom | *Bungarus multicinctus, Naja atra* | Taiwan | Centres for Disease Control, Taiwan | 61-06-0002 | 23 Dec 2019 | Not reported |
| Liu BS, 2018 (27) | Elapidae | *Naja atra* | Taiwan | SAV-Naja | *Naja kaouthia* | Vietnam | Institute of Vaccines and Biological Substances (IVAC), Vietnam | 34 | 14 Aug 2016 | Not reported |
| Liu BS, 2018 (27) | Elapidae | *Naja atra* | Taiwan | Neuro polyvalent snake antivenom | *Ophiophagus Hannah, Naja Kaouthia, Bungarus fasciatus, Bungarus candidus* | Thailand | Queen Saovabha Memorial Institute, Thailand | NP00115 | 24 Mar 2020 | Not reported |
| Liu BS, 2018 (27) | Elapidae | *Naja atra* | Taiwan | Doboia siamensis Monovalent Snake Antivenom | *Doboia siamensis* | Taiwan | Centres for Disease Control, Taiwan | 62-06-0006 | 23 Dec 2019 | Not reported |
| Faisal T, 2018 (28) | Viperidae | *Daboia russelii* | Pakistan | Snake Venom Antiserum I.P. (Asia) | *Bungarus caeruleus, Daboia russelii, Echis carinatus, Naja naja* | India | VINS Bioproducts Ltd., India | 01AS12041 | Mar 2016 | 20 |
| Tan CH, 2017 (29) | Viperidae | *Trimeresurus insularis* | Indonesia | Serum Anti Bisa Ular (Biosave) | *Naja sputatrix, Calloselasma rhodostoma, Bungarus fasciatus* | Indonesia | Bio Farma, Indonesia | 4701314 | Oct 2016 | 104.3 ± 0.5 |
| Tan CH, 2017 (29) | Viperidae | *Trimeresurus purpureomaculatus* | Indonesia | Serum Anti Bisa Ular (Biosave) | *Naja sputatrix, Calloselasma rhodostoma, Bungarus fasciatus* | Indonesia | Bio Farma, Indonesia | 4701314 | Oct 2016 | 104.3 ± 0.5 |
| Tan CH, 2017 (29) | Viperidae | *Trimeresurus hageni* | Indonesia | Serum Anti Bisa Ular (Biosave) | *Naja sputatrix, Calloselasma rhodostoma, Bungarus fasciatus* | Indonesia | Bio Farma, Indonesia | 4701314 | Oct 2016 | 104.3 ± 0.5 |
| Tan CH, 2017 (29) | Viperidae | *Trimeresurus puniceus* | Indonesia | Serum Anti Bisa Ular (Biosave) | *Naja sputatrix, Calloselasma rhodostoma, Bungarus fasciatus* | Indonesia | Bio Farma, Indonesia | 4701314 | Oct 2016 | 104.3 ± 0.5 |
| Tan CH, 2017 (29) | Viperidae | *Trimeresurus insularis* | Indonesia | Green pit viper antivenin | *Trimeresurus albolabris* | Thailand | Queen Saovabha Memorial Institute, Thailand | TA00312 | Jan 2018 | 20.22 ± 0.7 |
| Tan CH, 2017 (29) | Viperidae | *Trimeresurus purpureomaculatus* | Indonesia | Green pit viper antivenin | *Trimeresurus albolabris* | Thailand | Queen Saovabha Memorial Institute, Thailand | TA00312 | Jan 2018 | 20.22 ± 0.7 |
| Tan CH, 2017 (29) | Viperidae | *Trimeresurus hageni* | Indonesia | Green pit viper antivenin | *Trimeresurus albolabris* | Thailand | Queen Saovabha Memorial Institute, Thailand | TA00312 | Jan 2018 | 20.22 ± 0.7 |
| Tan CH, 2017 (29) | Viperidae | *Trimeresurus puniceus* | Indonesia | Green pit viper antivenin | *Trimeresurus albolabris* | Thailand | Queen Saovabha Memorial Institute, Thailand | TA00312 | Jan 2018 | 20.22 ± 0.7 |
| Tan CH, 2017 (30) | Elapidae | *Laticauda colubrina* | Indonesia | Sea Snake Antivenom | *Enhydrina schistosa* | Australia | 0549-08201 | Apr 2015 | Mar 2016 | 217.2 ± 3 |
| Oh AMF, 2017 (31) | Elapidae | *Bungarus caeruleus* | Sri Lanka | Snake Venom Antiserum I.P. (Asia) | *Bungarus caeruleus, Daboia russelii, Echis carinatus, Naja naja* | India | VINS Bioproducts Ltd., India | 01AS12041 | Mar 2016 | Not reported |
| Oh AMF, 2017 (31) | Elapidae | *Bungarus caeruleus* | India | Snake Venom Antiserum I.P. (Asia) | *Bungarus caeruleus, Daboia russelii, Echis carinatus, Naja naja* | India | VINS Bioproducts Ltd., India | 01AS12041 | Mar 2016 | Not reported |
| Oh AMF, 2017 (31) | Elapidae | *Bungarus caeruleus* | Pakistan | Snake Venom Antiserum I.P. (Asia) | *Bungarus caeruleus, Daboia russelii, Echis carinatus, Naja naja* | India | VINS Bioproducts Ltd., India | 01AS12041 | Mar 2016 | Not reported |
| Wong KY, 2016 (32) | Elapidae | *Naja naja* | Pakistan | Snake Venom Antiserum I.P. (Asia) | *Bungarus caeruleus, Daboia russelii, Echis carinatus, Naja naja* | India | VINS Bioproducts Ltd., India | 01AS12041 | Mar 2016 | Not reported |
| Wong KY, 2016 (32) | Elapidae | *Naja naja* | Pakistan | Cobra antivenin | *Naja kaouthia* | Thailand | Queen Saovabha Memorial Institute, Thailand | NK00514 | Oct 2019 | Not reported |
| Wong KY, 2016 (32) | Elapidae | *Naja naja* | Pakistan | Neuro bivalent antivenom | *Bungarus multicinctus, Naja atra* | Taiwan | Centres for Disease Control, Taiwan | FN10101 | Apr 2017 | Not reported |
| Villalta M, 2016 (33) | Viperidae | *Daboia russelii* | Sri Lanka | Snake Venom Antiserum I.P. (Asia) | *Bungarus caeruleus, Daboia russelii, Echis carinatus, Naja naja* | India | VINS Bioproducts Ltd., India | 01AS13100 | Nov 2017 | 41.90 ± 0.04 |
| Villalta M, 2016 (33) | Elapidae | *Echis carinatus* | Sri Lanka | Snake Venom Antiserum I.P. (Asia) | *Bungarus caeruleus, Daboia russelii, Echis carinatus, Naja naja* | India | VINS Bioproducts Ltd., India | 01AS13100 | Nov 2017 | 41.90 ± 0.04 |
| Villalta M, 2016 (33) | Viperidae | *Hypnale hynale* | Sri Lanka | Snake Venom Antiserum I.P. (Asia) | *Bungarus caeruleus, Daboia russelii, Echis carinatus, Naja naja* | India | VINS Bioproducts Ltd., India | 01AS13100 | Nov 2017 | 41.90 ± 0.04 |
| Villalta M, 2016 (33) | Elapidae | *Naja naja* | Sri Lanka | Snake Venom Antiserum I.P. (Asia) | *Bungarus caeruleus, Daboia russelii, Echis carinatus, Naja naja* | India | VINS Bioproducts Ltd., India | 01AS13100 | Nov 2017 | 41.90 ± 0.04 |
| Tan KY, 2016 (34) | Elapidae | *Naja kaouthia* | Thailand | Cobra antivenin | *Naja kaouthia* | Thailand | Queen Saovabha Memorial Institute, Thailand | 80210 | 9 Aug 2015 | 45.00 ± 0.60 |
| Tan KY, 2016 (34) | Elapidae | *Naja kaouthia* | Thailand | Sea Snake Antivenom | *Enhydrina schistosa* | Australia | CSL Ltd., Australia | 0080210 | 9 Aug 2015 | 217.2 ± 3 |
| Tan CH, 2016 (35) | Elapidae | *Naja sputatrix* | Indonesia (Java Island) | Serum Anti Bisa Ular (Biosave) | *Naja sputatrix, Calloselasma rhodostoma, Bungarus fasciatus* | Indonesia | Bio Farma, Indonesia | 4701314 | Oct 2016 | 104.3 ± 0.5 |
| Tan CH, 2016 (35) | Elapidae | *Bungarus fasciatus* | Indonesia (Java Island) | Serum Anti Bisa Ular (Biosave) | *Naja sputatrix, Calloselasma rhodostoma, Bungarus fasciatus* | Indonesia | Bio Farma, Indonesia | 4701314 | Oct 2016 | 104.3 ± 0.5 |
| Tan CH, 2016 (35) | Viperidae | *Calloselasma rhodostoma* | Indonesia (Java Island) | Serum Anti Bisa Ular (Biosave) | *Naja sputatrix, Calloselasma rhodostoma, Bungarus fasciatus* | Indonesia | Bio Farma, Indonesia | 4701314 | Oct 2016 | 104.3 ± 0.5 |
| Tan CH, 2016 (35) | Elapidae | *Naja sputatrix* | Indonesia (Java Island) | Neuro-polyvalent snake antivenom | *Naja kaouthia, Ophiophagus hannah, Bungarus candidus, and Bungarus fasciatus* | Thailand | Queen Saovabha Memorial Institute, Thailand | NP00414 | 9 Dec 2019 | 75.3 ± 0.6 |
| Tan CH, 2016 (35) | Elapidae | *Bungarus fasciatus* | Indonesia (Java Island) | Neuro-polyvalent snake antivenom | *Naja kaouthia, Ophiophagus hannah, Bungarus candidus, and Bungarus fasciatus* | Thailand | Queen Saovabha Memorial Institute, Thailand | NP00414 | 9 Dec 2019 | 75.3 ± 0.6 |
| Tan CH, 2016 (35) | Viperidae | *Calloselasma rhodostoma* | Indonesia (Java Island) | Haemato-polyvalent snake antivenom | *Calloselasma rhodostoma, Trimeresurus albolabris, and Daboia siamensis* | Thailand | Queen Saovabha Memorial Institute, Thailand | HP00216 | 8 Mar 2021 | 43 ± 0.5 |
| Tan CH, 2016 (35) | Elapidae | *Naja sumatrana* | Indonesia (Sumatra) | Serum Anti Bisa Ular (Biosave) | *Naja sputatrix, Calloselasma rhodostoma, Bungarus fasciatus* | Indonesia | Bio Farma, Indonesia | 4701314 | Oct 2016 | 104.3 ± 0.5 |
| Tan CH, 2016 (35) | Elapidae | *Bungarus candidus* | Indonesia (Java Island) | Serum Anti Bisa Ular (Biosave) | *Naja sputatrix, Calloselasma rhodostoma, Bungarus fasciatus* | Indonesia | Bio Farma, Indonesia | 4701314 | Oct 2016 | 104.3 ± 0.5 |
| Tan CH, 2016 (35) | Elapidae | *Naja sumatrana* | Indonesia (Sumatra) | Neuro-polyvalent snake antivenom | *Naja kaouthia, Ophiophagus hannah, Bungarus candidus, and Bungarus fasciatus* | Thailand | Queen Saovabha Memorial Institute, Thailand | NP00414 | 9 Dec 2019 | 75.3 ± 0.6 |
| Tan CH, 2016 (35) | Elapidae | *Bungarus candidus* | Indonesia (Java Island) | Neuro-polyvalent snake antivenom | *Naja kaouthia, Ophiophagus hannah, Bungarus candidus, and Bungarus fasciatus* | Thailand | Queen Saovabha Memorial Institute, Thailand | NP00414 | 9 Dec 2019 | 75.3 ± 0.6 |
| Tan CH, 2016 (36) | Elapidae | *Hydrophis schistosus* | Malaysia | Sea Snake Antivenom | *Enhydrina schistosa* | Australia | CSL Ltd., Australia | 0549-08201 | Apr 2015 | Not reported |
| Maduwage K, 2016 (37) | Viperidae | *Daboia russelii* | Sri Lanka | Snake Venom Antiserum I.P. (Asia) | *Bungarus caeruleus, Daboia russelii, Echis carinatus, Naja naja* | India | VINS Bioproducts Ltd., India | 01011/10-11 | 2010 | 198 (mg/vial) |
| Maduwage K, 2016 (37) | Viperidae | *Echis carinatus* | Sri Lanka | Snake Venom Antiserum I.P. (Asia) | *Bungarus caeruleus, Daboia russelii, Echis carinatus, Naja naja* | India | VINS Bioproducts Ltd., India | 01011/10-11 | 2010 | 198 (mg/vial) |
| Maduwage K, 2016 (37) | Elapidae | *Naja naja* | Sri Lanka | Snake Venom Antiserum I.P. (Asia) | *Bungarus caeruleus, Daboia russelii, Echis carinatus, Naja naja* | India | VINS Bioproducts Ltd., India | 01011/10-11 | 2010 | 198 (mg/vial) |
| Maduwage K, 2016 (37) | Elapidae | *Bungarus caeruleus* | Sri Lanka | Snake Venom Antiserum I.P. (Asia) | *Bungarus caeruleus, Daboia russelii, Echis carinatus, Naja naja* | India | VINS Bioproducts Ltd., India | 01011/10-11 | 2010 | 198 (mg/vial) |
| Maduwage K, 2016 (37) | Viperidae | *Daboia russelii* | Sri Lanka | Polyvalent Snake Antivenom (Asia) | *Bungarus caeruleus, Daboia russelii, Echis carinatus, Naja naja* | India | Bharat Serums & Vaccines, India | A5311006 | Jan 2011 | 98 (mg/vial) |
| Maduwage K, 2016 (37) | Viperidae | *Echis carinatus* | Sri Lanka | Polyvalent Snake Antivenom (Asia) | *Bungarus caeruleus, Daboia russelii, Echis carinatus, Naja naja* | India | Bharat Serums & Vaccines, India | A5311006 | Jan 2011 | 98 (mg/vial) |
| Maduwage K, 2016 (37) | Elapidae | *Naja naja* | Sri Lanka | Polyvalent Snake Antivenom (Asia) | *Bungarus caeruleus, Daboia russelii, Echis carinatus, Naja naja* | India | Bharat Serums & Vaccines, India | A5311006 | Jan 2011 | 98 (mg/vial) |
| Maduwage K, 2016 (37) | Elapidae | *Bungarus caeruleus* | Sri Lanka | Polyvalent Snake Antivenom (Asia) | *Bungarus caeruleus, Daboia russelii, Echis carinatus, Naja naja* | India | Bharat Serums & Vaccines, India | A5311006 | Jan 2011 | 98 (mg/vial) |
| Yap MK, 2015 (38) | Elapidae | *Naja sputatrix* | Not reported (Studies from Malaysia) | Neuro-polyvalent snake antivenom | *Naja kaouthia, Ophiophagus hannah, Bungarus candidus, and Bungarus fasciatus* | Thailand | Queen Saovabha Memorial Institute, Thailand | 0030208 | Not reported | Not reported |
| Tan KY, 2015 (39) | Elapidae | *Naja kaouthia* | Malaysia | Cobra antivenin | *Naja kaouthia* | Thailand | Queen Saovabha Memorial Institute, Thailand | 0080210 | 9 Aug 2015 | Not reported |
| Tan KY, 2015 (39) | Elapidae | *Naja kaouthia* | Thailand | Cobra antivenin | *Naja kaouthia* | Thailand | Queen Saovabha Memorial Institute, Thailand | 0080210 | 9 Aug 2015 | Not reported |
| Tan KY, 2015 (39) | Elapidae | *Naja kaouthia* | Vietnam | Cobra antivenin | *Naja kaouthia* | Thailand | Queen Saovabha Memorial Institute, Thailand | 0080210 | 9 Aug 2015 | Not reported |
| Tan KY, 2015 (39) | Elapidae | *Naja kaouthia* | Malaysia | Neuro-polyvalent snake antivenom | *Naja kaouthia, Ophiophagus hannah, Bungarus candidus, and Bungarus fasciatus* | Thailand | Queen Saovabha Memorial Institute, Thailand | 0020208 | 5 Oct 2014 | Not reported |
| Tan KY, 2015 (39) | Elapidae | *Naja kaouthia* | Thailand | Neuro-polyvalent snake antivenom | *Naja kaouthia, Ophiophagus hannah, Bungarus candidus, and Bungarus fasciatus* | Thailand | Queen Saovabha Memorial Institute, Thailand | 0020208 | 5 Oct 2014 | Not reported |
| Tan KY, 2015 (39) | Elapidae | *Naja kaouthia* | Vietnam | Neuro-polyvalent snake antivenom | *Naja kaouthia, Ophiophagus hannah, Bungarus candidus, and Bungarus fasciatus* | Thailand | Queen Saovabha Memorial Institute, Thailand | 0020208 | 5 Oct 2014 | Not reported |
| Tan CH, 2015 (40) | Elapidae | *Hydrophis schistosus* | Malaysia | Neuro bivalent antivenom | *Bungarus multicinctus, Naja atra* | Taiwan | Centres for Disease Control, Taiwan | FN10001 | 31 Mar 2016 | Not reported |
| Tan CH, 2015 (40) | Elapidae | *Hydrophis curtus* | Malaysia | Neuro bivalent antivenom | *Bungarus multicinctus, Naja atra* | Taiwan | Centres for Disease Control, Taiwan | FN10001 | 31 Mar 2016 | Not reported |
| Tan CH, 2015 (40) | Elapidae | *Hydrophis schistosus* | Malaysia | Cobra antivenin | *Naja kaouthia* | Thailand | Queen Saovabha Memorial Institute, Thailand | NK00310 | 9 Aug 2015 | Not reported |
| Tan CH, 2015 (40) | Elapidae | *Hydrophis curtus* | Malaysia | Cobra antivenin | *Naja kaouthia* | Thailand | Queen Saovabha Memorial Institute, Thailand | NK00310 | 9 Aug 2015 | Not reported |
| Tan CH, 2015 (40) | Elapidae | *Hydrophis schistosus* | Malaysia | Neuro-polyvalent snake antivenom | *Naja kaouthia, Ophiophagus hannah, Bungarus candidus, and Bungarus fasciatus* | Thailand | Queen Saovabha Memorial Institute, Thailand | NP00109 | 5 Oct 2014 | Not reported |
| Tan CH, 2015 (40) | Elapidae | *Hydrophis curtus* | Malaysia | Neuro-polyvalent snake antivenom | *Naja kaouthia, Ophiophagus hannah, Bungarus candidus, and Bungarus fasciatus* | Thailand | Queen Saovabha Memorial Institute, Thailand | NP00109 | 5 Oct 2014 | Not reported |
| Leong PK, 2015 (41) | Elapidae | *Naja sumatrana* | Malaysia | Neuro-polyvalent snake antivenom | *Naja kaouthia, Ophiophagus hannah, Bungarus candidus, and Bungarus fasciatus* | Thailand | Queen Saovabha Memorial Institute, Thailand | NP00109 | 10 May 2014 | Not reported |
| Leong PK, 2015 (41) | Elapidae | *Naja kaouthia* | Thailand | Neuro-polyvalent snake antivenom | *Naja kaouthia, Ophiophagus hannah, Bungarus candidus, and Bungarus fasciatus* | Thailand | Queen Saovabha Memorial Institute, Thailand | NP00109 | 10 May 2014 | Not reported |
| Leong PK, 2015 (41) | Elapidae | *Naja sputatrix* | Indonesia | Neuro-polyvalent snake antivenom | *Naja kaouthia, Ophiophagus hannah, Bungarus candidus, and Bungarus fasciatus* | Thailand | Queen Saovabha Memorial Institute, Thailand | NP00109 | 10 May 2014 | Not reported |
| Leong PK, 2014 (42) | Viperidae | *Calloselasma rhodostoma* | Malaysia | Haemato-polyvalent snake antivenom | *Calloselasma rhodostoma, Trimeresurus albolabris, and Daboia siamensis* | Thailand | Queen Saovabha Memorial Institute, Thailand | 0020107 | 6 Nov 2013 | Not reported |
| Leong PK, 2014 (42) | Viperidae | *Calloselasma rhodostoma* | Indonesia | Haemato-polyvalent snake antivenom | *Calloselasma rhodostoma, Trimeresurus albolabris, and Daboia siamensis* | Thailand | Queen Saovabha Memorial Institute, Thailand | 0020107 | 6 Nov 2013 | 19.82 ± 1.39 |
| Leong PK, 2014 (42) | Viperidae | *Trimeresurus albolabris* | Not reported | Haemato-polyvalent snake antivenom | *Calloselasma rhodostoma, Trimeresurus albolabris, and Daboia siamensis* | Thailand | Queen Saovabha Memorial Institute, Thailand | 0020107 | 6 Nov 2013 | 19.82 ± 1.39 |
| Leong PK, 2014 (42) | Viperidae | *Trimeresurus purpureomaculatus* | Not reported | Haemato-polyvalent snake antivenom | *Calloselasma rhodostoma, Trimeresurus albolabris, and Daboia siamensis* | Thailand | Queen Saovabha Memorial Institute, Thailand | 0020107 | 6 Nov 2013 | 19.82 ± 1.39 |
| Leong PK, 2014 (42) | Viperidae | *Trimeresurus popeiorum* | Not reported | Haemato-polyvalent snake antivenom | *Calloselasma rhodostoma, Trimeresurus albolabris, and Daboia siamensis* | Thailand | Queen Saovabha Memorial Institute, Thailand | 0020107 | 6 Nov 2013 | 19.82 ± 1.39 |
| Leong PK, 2014 (42) | Viperidae | *Tropidolaemus wagleri* | Not reported | Haemato-polyvalent snake antivenom | *Calloselasma rhodostoma, Trimeresurus albolabris, and Daboia siamensis* | Thailand | Queen Saovabha Memorial Institute, Thailand | 0020107 | 6 Nov 2013 | 19.82 ± 1.39 |
| Leong PK, 2014 (42) | Viperidae | *Daboia siamensis* | Thailand | Haemato-polyvalent snake antivenom | *Calloselasma rhodostoma, Trimeresurus albolabris, and Daboia siamensis* | Thailand | Queen Saovabha Memorial Institute, Thailand | 0020107 | 6 Nov 2013 | 19.82 ± 1.39 |
| Leong PK, 2014 (42) | Viperidae | *Daboia siamensis* | Myanmar | Haemato-polyvalent snake antivenom | *Calloselasma rhodostoma, Trimeresurus albolabris, and Daboia siamensis* | Thailand | Queen Saovabha Memorial Institute, Thailand | 0020107 | 6 Nov 2013 | 19.82 ± 1.39 |
| Leong PK, 2014 (42) | Viperidae | *Calloselasma rhodostoma* | Malaysia | Malayan pit viper antivenin | *Calloselasma rhodostoma* | Thailand | Queen Saovabha Memorial Institute, Thailand | 0120406 | 2 Nov 2014 | 14.53 ± 0.74 |
| Danpaiboon W, 2014 (43) | Elapidae | *Ophiophagus hannah* | Thailand | Cobra antivenin | *Naja kaouthia* | Thailand | Queen Saovabha Memorial Institute, Thailand | Not report | Not report | Not reported |
| Pakmanee N, 2013 (44) | Viperidae | *Daboia siamensis* | Thailand | Russell's viper antivenin | *Daboia siamensis* | Thailand | Queen Saovabha Memorial Institute, Thailand | Not report | Not report | Not reported |
| Leong PK, 2012 (45) | Elapidae | *Naja sputatrix* | Not reported | Snake Venom Antiserum I.P. (Asia) | *Bungarus caeruleus, Daboia russelii, Echis carinatus, Naja naja* | India | VINS Bioproducts Ltd., India | 01082/10-11 | 1 Nov 2014 | 19.47 ± 1.71 |
| Leong PK, 2012 (45) | Elapidae | *Naja siamensis* | Not reported | Snake Venom Antiserum I.P. (Asia) | *Bungarus caeruleus, Daboia russelii, Echis carinatus, Naja naja* | India | VINS Bioproducts Ltd., India | 01082/10-11 | 1 Nov 2014 | 19.47 ± 1.71 |
| Leong PK, 2012 (45) | Elapidae | *Naja kaouthia* | Thailand | Snake Venom Antiserum I.P. (Asia) | *Bungarus caeruleus, Daboia russelii, Echis carinatus, Naja naja* | India | VINS Bioproducts Ltd., India | 01082/10-11 | 1 Nov 2014 | 19.47 ± 1.71 |
| Leong PK, 2012 (45) | Elapidae | *Naja kaouthia* | Malaysia | Snake Venom Antiserum I.P. (Asia) | *Bungarus caeruleus, Daboia russelii, Echis carinatus, Naja naja* | India | VINS Bioproducts Ltd., India | 01082/10-11 | 1 Nov 2014 | 19.47 ± 1.71 |
| Leong PK, 2012 (45) | Elapidae | *Naja sumatrana* | Malaysia | Snake Venom Antiserum I.P. (Asia) | *Bungarus caeruleus, Daboia russelii, Echis carinatus, Naja naja* | India | VINS Bioproducts Ltd., India | 01082/10-11 | 1 Nov 2014 | 19.47 ± 1.71 |
| Leong PK, 2012 (45) | Elapidae | *Naja philippinensis* | Not reported | Snake Venom Antiserum I.P. (Asia) | *Bungarus caeruleus, Daboia russelii, Echis carinatus, Naja naja* | India | VINS Bioproducts Ltd., India | 01082/10-11 | 1 Nov 2014 | 19.47 ± 1.71 |
| Leong PK, 2012 (45) | Elapidae | *Ophiophagus hannah* | Not reported | Snake Venom Antiserum I.P. (Asia) | *Bungarus caeruleus, Daboia russelii, Echis carinatus, Naja naja* | India | VINS Bioproducts Ltd., India | 01082/10-11 | 1 Nov 2014 | 19.47 ± 1.71 |
| Leong PK, 2012 (45) | Elapidae | *Bungarus fasciatus* | Not reported | Snake Venom Antiserum I.P. (Asia) | *Bungarus caeruleus, Daboia russelii, Echis carinatus, Naja naja* | India | VINS Bioproducts Ltd., India | 01082/10-11 | 1 Nov 2014 | 19.47 ± 1.71 |
| Leong PK, 2012 (45) | Elapidae | *Bungarus candidus* | Not reported | Snake Venom Antiserum I.P. (Asia) | *Bungarus caeruleus, Daboia russelii, Echis carinatus, Naja naja* | India | VINS Bioproducts Ltd., India | 01082/10-11 | 1 Nov 2014 | 19.47 ± 1.71 |
| Leong PK, 2012 (45) | Elapidae | *Naja naja* | India | Snake Venom Antiserum I.P. (Asia) | *Bungarus caeruleus, Daboia russelii, Echis carinatus, Naja naja* | India | VINS Bioproducts Ltd., India | 01082/10-11 | 1 Nov 2014 | 19.47 ± 1.71 |
| Leong PK, 2012 (45) | Elapidae | *Naja naja* | India | Snake Venom Antiserum I.P. (Asia) | *Bungarus caeruleus, Daboia russelii, Echis carinatus, Naja naja* | India | VINS Bioproducts Ltd., India | 01082/10-11 | 1 Nov 2014 | 19.47 ± 1.71 |
| Leong PK, 2012 (45) | Elapidae | *Naja naja* | Sri Lanka | Snake Venom Antiserum I.P. (Asia) | *Bungarus caeruleus, Daboia russelii, Echis carinatus, Naja naja* | India | VINS Bioproducts Ltd., India | 01082/10-11 | 1 Nov 2014 | 19.47 ± 1.71 |
| Leong PK, 2012 (45) | Elapidae | *Naja naja* | Sri Lanka | Snake Venom Antiserum I.P. (Asia) | *Bungarus caeruleus, Daboia russelii, Echis carinatus, Naja naja* | India | VINS Bioproducts Ltd., India | 01082/10-11 | 1 Nov 2014 | 19.47 ± 1.71 |
| Leong PK, 2012 (45) | Elapidae | *Naja sputatrix* | Not reported | Polyvalent Snake Antivenom (Asia) | *Bungarus caeruleus, Daboia russelii, Echis carinatus, Naja naja* | India | Bharat Serums & Vaccines, India | A5309049 | Mar 2013 | 7.04 ± 1.71 |
| Leong PK, 2012 (45) | Elapidae | *Naja siamensis* | Not reported | Polyvalent Snake Antivenom (Asia) | *Bungarus caeruleus, Daboia russelii, Echis carinatus, Naja naja* | India | Bharat Serums & Vaccines, India | A5309049 | Mar 2013 | 7.04 ± 1.71 |
| Leong PK, 2012 (45) | Elapidae | *Naja kaouthia* | Thailand | Polyvalent Snake Antivenom (Asia) | *Bungarus caeruleus, Daboia russelii, Echis carinatus, Naja naja* | India | Bharat Serums & Vaccines, India | A5309049 | Mar 2013 | 7.04 ± 1.71 |
| Leong PK, 2012 (45) | Elapidae | *Naja kaouthia* | Malaysia | Polyvalent Snake Antivenom (Asia) | *Bungarus caeruleus, Daboia russelii, Echis carinatus, Naja naja* | India | Bharat Serums & Vaccines, India | A5309049 | Mar 2013 | 7.04 ± 1.71 |
| Leong PK, 2012 (45) | Elapidae | *Naja sumatrana* | Malaysia | Polyvalent Snake Antivenom (Asia) | *Bungarus caeruleus, Daboia russelii, Echis carinatus, Naja naja* | India | Bharat Serums & Vaccines, India | A5309049 | Mar 2013 | 7.04 ± 1.71 |
| Leong PK, 2012 (45) | Elapidae | *Naja philippinensis* | Not reported | Polyvalent Snake Antivenom (Asia) | *Bungarus caeruleus, Daboia russelii, Echis carinatus, Naja naja* | India | Bharat Serums & Vaccines, India | A5309049 | Mar 2013 | 7.04 ± 1.71 |
| Leong PK, 2012 (45) | Elapidae | *Ophiophagus hannah* | Not reported | Polyvalent Snake Antivenom (Asia) | *Bungarus caeruleus, Daboia russelii, Echis carinatus, Naja naja* | India | Bharat Serums & Vaccines, India | A5309049 | Mar 2013 | 7.04 ± 1.71 |
| Leong PK, 2012 (45) | Elapidae | *Bungarus fasciatus* | Not reported | Polyvalent Snake Antivenom (Asia) | *Bungarus caeruleus, Daboia russelii, Echis carinatus, Naja naja* | India | Bharat Serums & Vaccines, India | A5309049 | Mar 2013 | 7.04 ± 1.71 |
| Leong PK, 2012 (45) | Elapidae | *Bungarus candidus* | Not reported | Polyvalent Snake Antivenom (Asia) | *Bungarus caeruleus, Daboia russelii, Echis carinatus, Naja naja* | India | Bharat Serums & Vaccines, India | A5309049 | Mar 2013 | 7.04 ± 1.71 |
| Leong PK, 2012 (45) | Elapidae | *Naja naja* | India | Polyvalent Snake Antivenom (Asia) | *Bungarus caeruleus, Daboia russelii, Echis carinatus, Naja naja* | India | Bharat Serums & Vaccines, India | A5309049 | Mar 2013 | 7.04 ± 1.71 |
| Leong PK, 2012 (45) | Elapidae | *Naja naja* | India | Polyvalent Snake Antivenom (Asia) | *Bungarus caeruleus, Daboia russelii, Echis carinatus, Naja naja* | India | Bharat Serums & Vaccines, India | A5309049 | Mar 2013 | 7.04 ± 1.71 |
| Leong PK, 2012 (45) | Elapidae | *Naja naja* | Sri Lanka | Polyvalent Snake Antivenom (Asia) | *Bungarus caeruleus, Daboia russelii, Echis carinatus, Naja naja* | India | Bharat Serums & Vaccines, India | A5309049 | Mar 2013 | 7.04 ± 1.71 |
| Leong PK, 2012 (45) | Elapidae | *Naja naja* | Sri Lanka | Polyvalent Snake Antivenom (Asia) | *Bungarus caeruleus, Daboia russelii, Echis carinatus, Naja naja* | India | Bharat Serums & Vaccines, India | A5309049 | Mar 2013 | 7.04 ± 1.71 |
| Leong PK, 2012 (46) | Elapidae | *Naja sputatrix* | Not reported | Neuro-polyvalent snake antivenom | *Naja kaouthia, Ophiophagus hannah, Bungarus candidus, and Bungarus fasciatus* | Thailand | Queen Saovabha Memorial Institute, Thailand | 0030208 | 21 Apr 2013 | 20.3 |
| Leong PK, 2012 (46) | Elapidae | *Naja siamensis* | Not reported | Neuro-polyvalent snake antivenom | *Naja kaouthia, Ophiophagus hannah, Bungarus candidus, and Bungarus fasciatus* | Thailand | Queen Saovabha Memorial Institute, Thailand | 0030208 | 21 Apr 2013 | 20.3 |
| Leong PK, 2012 (46) | Elapidae | *Naja sumatrana* | Malaysia | Neuro-polyvalent snake antivenom | *Naja kaouthia, Ophiophagus hannah, Bungarus candidus, and Bungarus fasciatus* | Thailand | Queen Saovabha Memorial Institute, Thailand | 0030208 | 21 Apr 2013 | 20.3 |
| Leong PK, 2012 (46) | Elapidae | *Naja kaouthia* | Thailand | Neuro-polyvalent snake antivenom | *Naja kaouthia, Ophiophagus hannah, Bungarus candidus, and Bungarus fasciatus* | Thailand | Queen Saovabha Memorial Institute, Thailand | 0030208 | 21 Apr 2013 | 20.3 |
| Leong PK, 2012 (46) | Elapidae | *Naja kaouthia* | Malaysia | Neuro-polyvalent snake antivenom | *Naja kaouthia, Ophiophagus hannah, Bungarus candidus, and Bungarus fasciatus* | Thailand | Queen Saovabha Memorial Institute, Thailand | 0030208 | 21 Apr 2013 | 20.3 |
| Leong PK, 2012 (46) | Elapidae | *Naja philippinensis* | Not reported | Neuro-polyvalent snake antivenom | *Naja kaouthia, Ophiophagus hannah, Bungarus candidus, and Bungarus fasciatus* | Thailand | Queen Saovabha Memorial Institute, Thailand | 0030208 | 21 Apr 2013 | 20.3 |
| Leong PK, 2012 (46) | Elapidae | *Naja atra* | Not reported | Neuro-polyvalent snake antivenom | *Naja kaouthia, Ophiophagus hannah, Bungarus candidus, and Bungarus fasciatus* | Thailand | Queen Saovabha Memorial Institute, Thailand | 0030208 | 21 Apr 2013 | 20.3 |
| Leong PK, 2012 (46) | Elapidae | *Naja oxiana* | Not reported | Neuro-polyvalent snake antivenom | *Naja kaouthia, Ophiophagus hannah, Bungarus candidus, and Bungarus fasciatus* | Thailand | Queen Saovabha Memorial Institute, Thailand | 0030208 | 21 Apr 2013 | 20.3 |
| Leong PK, 2012 (46) | Elapidae | *Naja naja* | India | Neuro-polyvalent snake antivenom | *Naja kaouthia, Ophiophagus hannah, Bungarus candidus, and Bungarus fasciatus* | Thailand | Queen Saovabha Memorial Institute, Thailand | 0030208 | 21 Apr 2013 | 20.3 |
| Leong PK, 2012 (46) | Elapidae | *Naja naja* | India | Neuro-polyvalent snake antivenom | *Naja kaouthia, Ophiophagus hannah, Bungarus candidus, and Bungarus fasciatus* | Thailand | Queen Saovabha Memorial Institute, Thailand | 0030208 | 21 Apr 2013 | 20.3 |
| Leong PK, 2012 (46) | Elapidae | *Naja naja* | Sri Lanka | Neuro-polyvalent snake antivenom | *Naja kaouthia, Ophiophagus hannah, Bungarus candidus, and Bungarus fasciatus* | Thailand | Queen Saovabha Memorial Institute, Thailand | 0030208 | 21 Apr 2013 | 20.3 |
| Leong PK, 2012 (46) | Elapidae | *Naja naja* | Sri Lanka | Neuro-polyvalent snake antivenom | *Naja kaouthia, Ophiophagus hannah, Bungarus candidus, and Bungarus fasciatus* | Thailand | Queen Saovabha Memorial Institute, Thailand | 0030208 | 21 Apr 2013 | 20.3 |
| Leong PK, 2012 (46) | Elapidae | *Naja haje* | Not reported | Neuro-polyvalent snake antivenom | *Naja kaouthia, Ophiophagus hannah, Bungarus candidus, and Bungarus fasciatus* | Thailand | Queen Saovabha Memorial Institute, Thailand | 0030208 | 21 Apr 2013 | 20.3 |
| Leong PK, 2012 (46) | Elapidae | *Naja melanoleuca* | Not reported | Neuro-polyvalent snake antivenom | *Naja kaouthia, Ophiophagus hannah, Bungarus candidus, and Bungarus fasciatus* | Thailand | Queen Saovabha Memorial Institute, Thailand | 0030208 | 21 Apr 2013 | 20.3 |
| Leong PK, 2012 (46) | Elapidae | *Naja nigricollis* | Not reported | Neuro-polyvalent snake antivenom | *Naja kaouthia, Ophiophagus hannah, Bungarus candidus, and Bungarus fasciatus* | Thailand | Queen Saovabha Memorial Institute, Thailand | 0030208 | 21 Apr 2013 | 20.3 |
| Leong PK, 2012 (46) | Elapidae | *Naja nubiae* | Not reported | Neuro-polyvalent snake antivenom | *Naja kaouthia, Ophiophagus hannah, Bungarus candidus, and Bungarus fasciatus* | Thailand | Queen Saovabha Memorial Institute, Thailand | 0030208 | 21 Apr 2013 | 20.3 |
| Leong PK, 2012 (46) | Elapidae | *Naja katiensis* | Not reported | Neuro-polyvalent snake antivenom | *Naja kaouthia, Ophiophagus hannah, Bungarus candidus, and Bungarus fasciatus* | Thailand | Queen Saovabha Memorial Institute, Thailand | 0030208 | 21 Apr 2013 | 20.3 |
| Leong PK, 2012 (46) | Elapidae | *Ophiophagus hannah* | Malaysia | Neuro-polyvalent snake antivenom | *Naja kaouthia, Ophiophagus hannah, Bungarus candidus, and Bungarus fasciatus* | Thailand | Queen Saovabha Memorial Institute, Thailand | 0030208 | 21 Apr 2013 | 20.3 |
| Leong PK, 2012 (46) | Elapidae | *Bungarus fasciatus* | Malaysia | Neuro-polyvalent snake antivenom | *Naja kaouthia, Ophiophagus hannah, Bungarus candidus, and Bungarus fasciatus* | Thailand | Queen Saovabha Memorial Institute, Thailand | 0030208 | 21 Apr 2013 | 20.3 |
| Leong PK, 2012 (46) | Elapidae | *Bungarus candidus* | Malaysia | Neuro-polyvalent snake antivenom | *Naja kaouthia, Ophiophagus hannah, Bungarus candidus, and Bungarus fasciatus* | Thailand | Queen Saovabha Memorial Institute, Thailand | 0030208 | 21 Apr 2013 | 20.3 |
| Leong PK, 2012 (46) | Elapidae | *Bungarus flaviceps* | Malaysia | Neuro-polyvalent snake antivenom | *Naja kaouthia, Ophiophagus hannah, Bungarus candidus, and Bungarus fasciatus* | Thailand | Queen Saovabha Memorial Institute, Thailand | 0030208 | 21 Apr 2013 | 20.3 |
| Leong PK, 2012 (46) | Elapidae | *Bungarus multicinctus* | Not reported | Neuro-polyvalent snake antivenom | *Naja kaouthia, Ophiophagus hannah, Bungarus candidus, and Bungarus fasciatus* | Thailand | Queen Saovabha Memorial Institute, Thailand | 0030208 | 21 Apr 2013 | 20.3 |
| Leong PK, 2012 (46) | Elapidae | *Bungarus caeruleus* | Not reported | Neuro-polyvalent snake antivenom | *Naja kaouthia, Ophiophagus hannah, Bungarus candidus, and Bungarus fasciatus* | Thailand | Queen Saovabha Memorial Institute, Thailand | 0030208 | 21 Apr 2013 | 20.3 |
| Leong PK, 2012 (46) | Elapidae | *Naja sputatrix* | Not reported | Cobra antivenin | *Naja kaouthia* | Thailand | Queen Saovabha Memorial Institute, Thailand | 0090406 | 31 Aug 2014 | 12.5 |
| Leong PK, 2012 (46) | Elapidae | *Naja siamensis* | Not reported | Cobra antivenin | *Naja kaouthia* | Thailand | Queen Saovabha Memorial Institute, Thailand | 0090406 | 31 Aug 2014 | 12.5 |
| Leong PK, 2012 (46) | Elapidae | *Naja sumatrana* | Malaysia | Cobra antivenin | *Naja kaouthia* | Thailand | Queen Saovabha Memorial Institute, Thailand | 0090406 | 31 Aug 2014 | 12.5 |
| Leong PK, 2012 (46) | Elapidae | *Naja kaouthia* | Thailand | Cobra antivenin | *Naja kaouthia* | Thailand | Queen Saovabha Memorial Institute, Thailand | 0090406 | 31 Aug 2014 | 12.5 |
| Leong PK, 2012 (46) | Elapidae | *Naja kaouthia* | Malaysia | Cobra antivenin | *Naja kaouthia* | Thailand | Queen Saovabha Memorial Institute, Thailand | 0090406 | 31 Aug 2014 | 12.5 |
| Leong PK, 2012 (46) | Elapidae | *Ophiophagus hannah* | Malaysia | Cobra antivenin | *Naja kaouthia* | Thailand | Queen Saovabha Memorial Institute, Thailand | 0090406 | 31 Aug 2014 | 12.5 |
| Tan CH, 2011 (47) | Viperidae | *Calloselasma rhodostoma* | Malaysia | Malayan pit viper antivenin | *Calloselasma rhodostoma* | Thailand | Queen Saovabha Memorial Institute, Thailand | CR00909 | 2 Nov 2014 | Not reported |
| Tan CH, 2011 (47) | Viperidae | *Hypnale hypnale* | Sri Lanka | Malayan pit viper antivenin | *Calloselasma rhodostoma* | Thailand | Queen Saovabha Memorial Institute, Thailand | CR00909 | 2 Nov 2014 | Not reported |
| Tan CH, 2011 (47) | Viperidae | *Calloselasma rhodostoma* | Malaysia | Haemato-polyvalent snake antivenom | *Calloselasma rhodostoma, Trimeresurus albolabris, and Daboia siamensis* | Thailand | Queen Saovabha Memorial Institute, Thailand | HP00108 | 6 Nov 2013 | Not reported |
| Tan CH, 2011 (47) | Viperidae | *Hypnale hypnale* | Sri Lanka | Haemato-polyvalent snake antivenom | *Calloselasma rhodostoma, Trimeresurus albolabris, and Daboia siamensis* | Thailand | Queen Saovabha Memorial Institute, Thailand | HP00108 | 6 Nov 2013 | Not reported |
| Tan CH, 2011 (47) | Viperidae | *Daboia russelii* | Sri Lanka | Haemato-polyvalent snake antivenom | *Calloselasma rhodostoma, Trimeresurus albolabris, and Daboia siamensis* | Thailand | Queen Saovabha Memorial Institute, Thailand | HP00108 | 6 Nov 2013 | Not reported |
| Tan CH, 2011 (47) | Viperidae | *Echis carinatus sochureki* | Pakistan | Haemato-polyvalent snake antivenom | *Calloselasma rhodostoma, Trimeresurus albolabris, and Daboia siamensis* | Thailand | Queen Saovabha Memorial Institute, Thailand | HP00108 | 6 Nov 2013 | Not reported |
| Chanhome L, 2002 (48) | Viperidae | *Trimeresurus albolabris* | Thailand | Green pit viper antivenin | *Trimeresurus albolabris* | Thailand | Queen Saovabha Memorial Institute, Thailand | 9 | 11 Jun 2003 | Not reported |
| Chanhome L, 2002 (48) | Viperidae | *Trimeresurus macrops* | Thailand | Green pit viper antivenin | *Trimeresurus albolabris* | Thailand | Queen Saovabha Memorial Institute, Thailand | 9 | 11 Jun 2003 | Not reported |
| Chanhome L, 2002 (48) | Viperidae | *Trimeresurus popeiorum* | Thailand | Green pit viper antivenin | *Trimeresurus albolabris* | Thailand | Queen Saovabha Memorial Institute, Thailand | 9 | 11 Jun 2003 | Not reported |
| Chanhome L, 2002 (48) | Viperidae | *Trimeresurus hageni* | Thailand | Green pit viper antivenin | *Trimeresurus albolabris* | Thailand | Queen Saovabha Memorial Institute, Thailand | 9 | 11 Jun 2003 | Not reported |
| Chanhome L, 2002 (48) | Viperidae | *Trimeresurus purpureomaculatus* | Thailand | Green pit viper antivenin | *Trimeresurus albolabris* | Thailand | Queen Saovabha Memorial Institute, Thailand | 9 | 11 Jun 2003 | Not reported |
| Chanhome L, 2002 (48) | Viperidae | *Trimeresurus kanburiensis* | Thailand | Green pit viper antivenin | *Trimeresurus albolabris* | Thailand | Queen Saovabha Memorial Institute, Thailand | 9 | 11 Jun 2003 | Not reported |
| Khow O, 2001 (49) | Elapidae | *Naja kaouthia* | Thailand | Cobra antivenin | *Naja kaouthia* | Thailand | Queen Saovabha Memorial Institute, Thailand | 3 | 11 Feb 2002 | Not reported |
| Khow O, 2001 (49) | Elapidae | *Naja kaouthia* | Thailand | Cobra antivenin | *Naja kaouthia* | Thailand | Queen Saovabha Memorial Institute, Thailand | 4 | 16 Feb 2004 | Not reported |
| Khow O, 2001 (49) | Elapidae | *Naja kaouthia* | Thailand | Cobra antivenin | *Naja kaouthia* | Thailand | Queen Saovabha Memorial Institute, Thailand | 12 | 22 Dec 2003 | Not reported |
| Khow O, 2001 (49) | Elapidae | *Naja kaouthia* | Thailand | Cobra antivenin | *Naja kaouthia* | Thailand | Queen Saovabha Memorial Institute, Thailand | 13 | 23 Dec 2003 | Not reported |
| Khow O, 2001 (49) | Elapidae | *Lapemis hardwickii* | Japan | Cobra antivenin | *Naja kaouthia* | Thailand | Queen Saovabha Memorial Institute, Thailand | 3 | 1 Feb 2002 | Not reported |
| Khow O, 2001 (49) | Elapidae | *Lapemis hardwickii* | Japan | Cobra antivenin | *Naja kaouthia* | Thailand | Queen Saovabha Memorial Institute, Thailand | 4 | 16 Feb 2004 | Not reported |
| Khow O, 2001 (49) | Elapidae | *Lapemis hardwickii* | Japan | Cobra antivenin | *Naja kaouthia* | Thailand | Queen Saovabha Memorial Institute, Thailand | 12 | 22 Dec 2003 | Not reported |
| Khow O, 2001 (49) | Elapidae | *Lapemis hardwickii* | Japan | Cobra antivenin | *Naja kaouthia* | Thailand | Queen Saovabha Memorial Institute, Thailand | 13 | 23 Dec 2003 | Not reported |
| Chanhome L, 1999 (50) | Elapidae | *Bungarus fasciatus* | Thailand | Banded krait antivenin | *Bungarus fasciatus* | Thailand | Queen Saovabha Memorial Institute, Thailand | 2 | Not reported | Not reported |
| Chanhome L, 1999 (50) | Elapidae | *Bungarus candidus* | Thailand | Banded krait antivenin | *Bungarus fasciatus* | Thailand | Queen Saovabha Memorial Institute, Thailand | 2 | Not reported | Not reported |
| Chanhome L, 1999 (50) | Elapidae | *Bungarus flaviceps* | Thailand | Banded krait antivenin | *Bungarus fasciatus* | Thailand | Queen Saovabha Memorial Institute, Thailand | 2 | Not reported | Not reported |
| Khow O, 1997 (51) | Elapidae | *Naja kaouthia* | Thailand | Cobra antivenin | *Naja kaouthia* | Thailand | Queen Saovabha Memorial Institute, Thailand | 51 | Not reported | Not reported |
| Khow O, 1997 (51) | Elapidae | *Naja siamensis* | Thailand | Cobra antivenin | *Naja kaouthia* | Thailand | Queen Saovabha Memorial Institute, Thailand | 51 | Not reported | Not reported |
| Khow O, 1997 (51) | Elapidae | *Naja kaouthia* | Thailand | Cobra antivenin | *Naja kaouthia* | Thailand | Queen Saovabha Memorial Institute, Thailand | 10 | Not reported | Not reported |
| Khow O, 1997 (51) | Elapidae | *Naja siamensis* | Thailand | Cobra antivenin | *Naja kaouthia* | Thailand | Queen Saovabha Memorial Institute, Thailand | 10 | Not reported | Not reported |
| Khow O, 1997 (51) | Elapidae | *Naja kaouthia* | Thailand | Cobra antivenin | *Naja kaouthia* | Thailand | Queen Saovabha Memorial Institute, Thailand | 2 | Not reported | Not reported |
| Khow O, 1997 (51) | Elapidae | *Naja siamensis* | Thailand | Cobra antivenin | *Naja kaouthia* | Thailand | Queen Saovabha Memorial Institute, Thailand | 2 | Not reported | Not reported |
| Sells PG, 1994 (52) | Elapidae | *Naja kaouthia* | Thailand | Cobra antivenin | *Naja kaouthia* | Thailand | Queen Saovabha Memorial Institute, Thailand | Not reported | Not reported | Not reported |

**References**

1. Tan KY, Shamsuddin NN, Tan CH. Sharp-nosed Pit Viper (Deinagkistrodon acutus) from Taiwan and China: A comparative study on venom toxicity and neutralization by two specific antivenoms across the Strait. Acta Trop. 2022;232:106495.

2. Chanhome L, Khow O, Reamtong O, Vasaruchapong T, Laoungbua P, Tawan T, et al. Biochemical and proteomic analyses of venom from a new pit viper, Protobothrops kelomohy. Journal of Venomous Animals and Toxins Including Tropical Diseases. 2022;28:14.

3. Wong KY, Tan KY, Tan NH, Gnanathasan CA, Tan CH. Elucidating the Venom Diversity in Sri Lankan Spectacled Cobra (Naja naja) through De Novo Venom Gland Transcriptomics, Venom Proteomics and Toxicity Neutralization. Toxins. 2021;13(8):30.

4. Faisal T, Tan KY, Tan NH, Sim SM, Gnanathasan CA, Tan CH. Proteomics, toxicity and antivenom neutralization of Sri Lankan and Indian Russell's viper (Daboia russelii) venoms. Journal of Venomous Animals and Toxins Including Tropical Diseases. 2021;27:15.

5. Attarde S, Khochare S, Iyer A, Dam P, Martin G, Sunagar K. Venomics of the Enigmatic Andaman Cobra (Naja sagittifera) and the Preclinical Failure of Indian Antivenoms in Andaman and Nicobar Islands. Frontiers in Pharmacology. 2021;12:16.

6. Tan CH, Palasuberniam P, Blanco FB, Tan KY. Immunoreactivity and neutralization capacity of Philippine cobra antivenom against Naja philippinensis and Naja samarensis venoms. Transactions of the Royal Society of Tropical Medicine and Hygiene. 2021;115(1):78-84.

7. Oh AMF, Tan KY, Tan NH, Tan CH. Proteomics and neutralization of Bungarus multicinctus (Many-banded Krait) venom: Intra-specific comparisons between specimens from China and Taiwan. Comparative Biochemistry and Physiology Part - C: Toxicology and Pharmacology. 2021;247.

8. Laxme RRS, Khochare S, Attarde S, Suranse V, Iyer A, Casewell NR, et al. Biogeographic venom variation in Russell's viper (Daboia russelii) and the preclinical inefficacy of antivenom therapy in snakebite hotspots. Plos Neglected Tropical Diseases. 2021;15(3).

9. Laxme RRS, Attarde S, Khochare S, Suranse V, Martin G, Casewell NR, et al. Biogeographical venom variation in the Indian spectacled cobra (Naja naja) underscores the pressing need for pan-India efficacious snakebite therapy. Plos Neglected Tropical Diseases. 2021;15(2).

10. Yee KT, Maw LZ, Kyaw AM, Khow O, Oo AW, Oo TKK, et al. Evaluation of the cross-neutralization capacity of Thai green pit viper antivenom against venom of Myanmar green pit viper. Toxicon. 2020;177:41-5.

11. Tan KY, Ng TS, Bourges A, Ismail AK, Maharani T, Khomvilai S, et al. Geographical variations in king cobra (Ophiophagus hannah) venom from Thailand, Malaysia, Indonesia and China: On venom lethality, antivenom immunoreactivity and in vivo neutralization. Acta Tropica. 2020;203.

12. Lin B, Zhang JR, Lu HJ, Zhao L, Chen J, Zhang HF, et al. Immunoreactivity and neutralization study of chinese bungarus multicinctus antivenin and lab-prepared anti-bungarotoxin antisera towards purified bungarotoxins and snake venoms. PLoS Neglected Tropical Diseases. 2020;14(11):1-19.

13. Liew JL, Tan NH, Tan CH. Proteomics and preclinical antivenom neutralization of the mangrove pit viper (Trimeresurus purpureomaculatus, Malaysia) and white-lipped pit viper (Trimeresurus albolabris, Thailand) venoms. Acta Tropica. 2020;209.

14. Lee LP, Tan KY, Tan CH. Toxicity and cross-neutralization of snake venoms from two lesser-known arboreal pit vipers in Southeast Asia: Trimeresurus wiroti and Trimeresurus puniceus. Toxicon. 2020;185:91-6.

15. Hia YL, Tan KY, Tan CH. Comparative venom proteomics of banded krait (Bungarus fasciatus) from five geographical locales: Correlation of venom lethality, immunoreactivity and antivenom neutralization. Acta Tropica. 2020;207.

16. Choraria A, Somasundaram R, Gautam M, Ramanathan M, Paray BA, Al-Sadoon MK, et al. Experimental antivenoms from chickens and rabbits and their comparison with commercially available equine antivenom against the venoms of Daboia russelii and Echis carinatus snakes. Toxin Reviews. 2020.

17. Tan CH, Tan KY, Ng TS, Quah ESH, Ismail AK, Khomvilai S, et al. Venomics of trimeresurus (Popeia) nebularis, the cameron highlands pit viper from Malaysia: Insights into venom proteome, toxicity and neutralization of antivenom. Toxins. 2019;11(2).

18. Pla D, Sanz L, Quesada-Bernat S, Villalta M, Baal J, Chowdhury MAW, et al. Phylovenomics of Daboia russelii across the Indian subcontinent. Bioactivities and comparative in vivo neutralization and in vitro third-generation antivenomics of antivenoms against venoms from India, Bangladesh and Sri Lanka. Journal of Proteomics. 2019;207.

19. Oh AMF, Tan CH, Tan KY, Quraishi NH, Tan NH. Venom proteome of Bungarus sindanus (Sind krait) from Pakistan and in vivo cross-neutralization of toxicity using an Indian polyvalent antivenom. Journal of Proteomics. 2019;193:243-54.

20. Lingam TMC, Tan KY, Tan CH. Thai Russell's viper monospecific antivenom is immunoreactive and effective in neutralizing the venom of Daboia siamensis from Java, Indonesia. Toxicon. 2019;168:95-7.

21. Laxme RRS, Khochare S, de Souza HF, Ahuja B, Suranse V, Martin G, et al. Beyond the 'big four': Venom profiling of the medically important yet neglected Indian snakes reveals disturbing antivenom deficiencies. Plos Neglected Tropical Diseases. 2019;13(12).

22. Deka A, Abu Reza M, Hoque KMF, Deka K, Saha S, Doley R. Comparative analysis of Naja kaouthia venom from North-East India and Bangladesh and its cross reactivity with Indian polyvalent antivenoms. Toxicon. 2019;164:31-43.

23. Chaisakul J, Alsolaiss J, Charoenpitakchai M, Wiwatwarayos K, Sookprasert N, Harrison RA, et al. Evaluation of the geographical utility of Eastern Russell’s viper (Daboia siamensis) antivenom from Thailand and an assessment of its protective effects against venom-induced nephrotoxicity. PLoS Neglected Tropical Diseases. 2019;13(10).

24. Tan CH, Tan KY, Ng TS, Sim SM, Tan NH. Venom Proteome of Spine-Bellied Sea Snake (Hydrophis curtus) from Penang, Malaysia: Toxicity Correlation, Immunoprofiling and Cross-Neutralization by Sea Snake Antivenom. Toxins (Basel). 2018;11(1).

25. Tan KY, Tan NH, Tan CH. Venom proteomics and antivenom neutralization for the Chinese eastern Russell's viper, Daboia siamensis from Guangxi and Taiwan. Scientific reports. 2018;8(1):8545.

26. Sanz L, Quesada-Bernat S, Chen PY, Lee CD, Chiang JR, Calvete JJ. Translational Venomics: Third-Generation Antivenomics of Anti-Siamese Russell's Viper, Daboia siamensis, Antivenom Manufactured in Taiwan CDC's Vaccine Center. Trop Med Infect Dis. 2018;3(2).

27. Liu BS, Wu WG, Lin MH, Li CH, Jiang BR, Wu SC, et al. Identification of immunoreactive peptides of toxins to simultaneously assess the neutralization potency of antivenoms against neurotoxicity and cytotoxicity of Naja atra venom. Toxins. 2018;10(1).

28. Faisal T, Tan KY, Sim SM, Quraishi N, Tan NH, Tan CH. Proteomics, functional characterization and antivenom neutralization of the venom of Pakistani Russell's viper (Daboia russelii) from the wild. Journal of Proteomics. 2018;183:1-13.

29. Tan CH, Liew JL, Tan NH, Ismail AK, Maharani T, Khomvilai S, et al. Cross reactivity and lethality neutralization of venoms of Indonesian Trimeresurus complex species by Thai Green Pit Viper Antivenom. Toxicon. 2017;140:32-7.

30. Tan CH, Wong KY, Tan KY, Tan NH. Venom proteome of the yellow-lipped sea krait, Laticauda colubrina from Bali: Insights into subvenomic diversity, venom antigenicity and cross-neutralization by antivenom. J Proteomics. 2017;166:48-58.

31. Oh AMF, Tan CH, Ariaranee GC, Quraishi N, Tan NH. Venomics of Bungarus caeruleus (Indian krait): Comparable venom profiles, variable immunoreactivities among specimens from Sri Lanka, India and Pakistan. Journal of Proteomics. 2017;164:1-18.

32. Wong KY, Tan CH, Tan NH. Venom and purified toxins of the spectacled cobra (Naja naja) from Pakistan: Insights into toxicity and antivenom neutralization. American Journal of Tropical Medicine and Hygiene. 2016;94(6):1392-9.

33. Villalta M, Sánchez A, Herrera M, Vargas M, Segura Á, Cerdas M, et al. Development of a new polyspecific antivenom for snakebite envenoming in Sri Lanka: Analysis of its preclinical efficacy as compared to a currently available antivenom. Toxicon. 2016;122:152-9.

34. Tan KY, Tan CH, Fung SY, Tan NH. Neutralization of the principal toxins from the venoms of thai naja kaouthia and malaysian hydrophis schistosus: Insights into toxin-specific neutralization by two different antivenoms. Toxins. 2016;8(4).

35. Tan CH, Liew JL, Tan KY, Tan NH. Assessing SABU (Serum Anti Bisa Ular), the sole Indonesian antivenom: A proteomic analysis and neutralization efficacy study. Scientific reports. 2016;6:37299.

36. Tan CH, Tan KY, Tan NH. Revisiting Notechis scutatus venom: on shotgun proteomics and neutralization by the "bivalent" Sea Snake Antivenom. J Proteomics. 2016;144:33-8.

37. Maduwage K, Silva A, O'Leary MA, Hodgson WC, Isbister GK. Efficacy of Indian polyvalent snake antivenoms against Sri Lankan snake venoms: lethality studies or clinically focussed in vitro studies. Scientific reports. 2016;6:26778.

38. Yap MK, Tan NH, Sim SM, Fung SY, Tan CH. The Effect of a Polyvalent Antivenom on the Serum Venom Antigen Levels of Naja sputatrix (Javan Spitting Cobra) Venom in Experimentally Envenomed Rabbits. Basic Clin Pharmacol Toxicol. 2015;117(4):274-9.

39. Tan KY, Tan CH, Fung SY, Tan NH. Venomics, lethality and neutralization of Naja kaouthia (monocled cobra) venoms from three different geographical regions of Southeast Asia. Journal of Proteomics. 2015;120:105-25.

40. Tan CH, Tan NH, Tan KY, Kwong KO. Antivenom cross-neutralization of the venoms of Hydrophis schistosus and Hydrophis curtus, two common sea snakes in Malaysian waters. Toxins. 2015;7(2):572-81.

41. Leong PK, Fung SY, Tan CH, Sim SM, Tan NH. Immunological cross-reactivity and neutralization of the principal toxins of Naja sumatrana and related cobra venoms by a Thai polyvalent antivenom (Neuro Polyvalent Snake Antivenom). Acta Tropica. 2015;149:86-93.

42. Leong PK, Tan CH, Sim SM, Fung SY, Sumana K, Sitprija V, et al. Cross neutralization of common Southeast Asian viperid venoms by a Thai polyvalent snake antivenom (Hemato Polyvalent Snake Antivenom). Acta Tropica. 2014;132(1):7-14.

43. Danpaiboon W, Reamtong O, Sookrung N, Seesuay W, Sakolvaree Y, Thanongsaksrikul J, et al. Ophiophagus hannah venom: Proteome, components bound by Naja kaouthia antivenin and neutralization by n. kaouthia neurotoxin-specific human ScFv. Toxins. 2014;6(5):1526-58.

44. Pakmanee N, Noiphrom J, Kay A, Pornmuttakun D, Sakolparp L, Hemmala W, et al. Comparative abilities of IgG and F(ab ')(2) monovalent antivenoms to neutralize lethality, phospholipase A(2), and coagulant activities induced by Daboia siamensis venom and their anticomplementary activity. Scienceasia. 2013;39(2):160-6.

45. Leong PK, Tan NH, Fung SY, Sim SM. Cross neutralisation of Southeast Asian cobra and krait venoms by Indian polyvalent antivenoms. Transactions of the Royal Society of Tropical Medicine and Hygiene. 2012;106(12):731-7.

46. Leong PK, Sim SM, Fung SY, Sumana K, Sitprija V, Tan NH. Cross neutralization of afro-asian cobra and asian krait venoms by a thai polyvalent snake antivenom (neuro polyvalent snake antivenom). PLoS Neglected Tropical Diseases. 2012;6(6).

47. Tan CH, Leong PK, Fung SY, Sim SM, Ponnudurai G, Ariaratnam C, et al. Cross neutralization of Hypnale hypnale (hump-nosed pit viper) venom by polyvalent and monovalent Malayan pit viper antivenoms in vitro and in a rodent model. Acta Tropica. 2011;117(2):119-24.

48. Chanhome L, Khow O, Omori-Satoh T, Sitprija V. Capacity of Thai green pit viper antivenom to neutralize the venoms of Thai Trimeresurus snakes and comparison of biological activities of these venoms. Journal of natural toxins. 2002;11(3):251-9.

49. Khow O, Chanhome L, Omori-Satoh T, Sitprija V. Effectiveness of Thai cobra (Naja kaouthia) antivenom against sea snake (Lapemis hardwickii) venom: verification by affinity purified F(AB')2 fragments. J Nat Toxins. 2001;10(3):249-53.

50. Chanhome L, Wongtongkam N, Khow O, Pakmanee N, Omori-Satoh T, Sitprija V. Genus specific neutralization of Bungarus snake venoms by Thai Red Cross banded krait antivenom. Journal of Natural Toxins. 1999;8(1):135-40.

51. Khow O, Pakmanee N, Chanhome L, Sriprapat S, Omori-Satoh T, Sitprija V. Cross-neutralization of Thai cobra (Naja kaouthia) and spitting cobra (Naja siamensis) venoms by Thai cobra antivenom. Toxicon. 1997;35(11):1649-51.

52. Sells PG, Jones RG, Laing GD, Smith DC, Theakston RD. Experimental evaluation of ovine antisera to Thai cobra (Naja kaouthia) venom and its alpha-neurotoxin. Toxicon. 1994;32(12):1657-65.
