# Supplementary material for: Antivenom preclinical efficacy testing against Asian snakes and their availability in Asia: A systematic review
Source: PLoS One. 2023 Jul 19;18(7):e0288723. doi: 10.1371/journal.pone.0288723 (PMC10355433; doi:10.1371/journal.pone.0288723)
Supplement: S4 Table — (DOCX) [file pone.0288723.s004.docx]

# **S4 Table. Results of antivenom neutralization against snake venom lethality from the included preclinical studies.**

| **Author, year** | **Snake species** | **Country** | **Route of admini-stration** | **LD50** | **Antivenom** | **Manufacturer** | **Chal-lenge dose** | **ED50 (μL)** | **ER50 (mg venom/ mL antivenom)** | **Potency (mg/mL)** | **Potency (mg/g)** |
| --- | --- | --- | --- | --- | --- | --- | --- | --- | --- | --- | --- |
| Tan KY, 2022 (1) | *Deinagkistrodon acutus* | Taiwan | I.V. | 8.27 (7.03-9.72) μg/g | Deinagkistrodon acutus Monovalent Antivenoms | Central for Disease Control, Taiwan | 2 | 2.78 | 136.84 (116.32-160.83) | 68.42 | 928.23 |
| Tan KY, 2022 (1) | *Deinagkistrodon acutus* | China | I.V. | 3.00 (2.74-3.28) μg/g | Deinagkistrodon acutus Monovalent Antivenoms | Central for Disease Control, Taiwan | 2 | 5.00 | 27.60 (25.21-30.18) | 13.80 | 187.22 |
| Tan KY, 2022 (1) | *Deinagkistrodon acutus* | Taiwan | I.V. | 8.27 (7.03-9.72) μg/g | Deinagkistrodon acutus Monovalent Antivenoms | Shanghai Serum Bio-Technology Co., Ltd., China | 2 | 6.88 | 60.15 (51.13-70.69) | 30.07 | 161.29 |
| Tan KY, 2022 (1) | *Deinagkistrodon acutus* | China | I.V. | 3.00 (2.74-3.28) μg/g | Deinagkistrodon acutus Monovalent Antivenoms | Shanghai Serum Bio-Technology Co., Ltd, China | 2 | 32.00 | 32.00 (29.23-34.99) | 16.00 | 85.82 |
| Chanhome O, 2022 (2) | *Protobothrops kelomohy* | Thailand (Northern Thailand) | I.V. | 0.67 (0.58-0.78) μg/g | Haemato-polyvalent snake antivenom | Queen Saovabha Memorial Institute, Thailand | 3 | 39.70 (32.13-49.08) | 1.02 (0.83-1.26) | Not reported | Not reported |
| Chanhome O, 2022 (2) | *Protobothrops kelomohy* | Thailand (Northern Thailand) | I.V. | 0.67 (0.58-0.78) μg/g | Russell's viper antivenin | Queen Saovabha Memorial Institute, Thailand | 3 | 111.33 (85.23-145.41) | 0.36 (0.28-0.48) | Not reported | Not reported |
| Chanhome O, 2022 (2) | *Protobothrops kelomohy* | Thailand (Northern Thailand) | I.V. | 0.67 (0.58-0.78) μg/g | Green pit viper antivenin | Queen Saovabha Memorial Institute, Thailand | 2.5 | 293.38 (234.96-356.59) | 0.12 (0.09-0.14) | Not reported | Not reported |
| Chanhome O, 2022 (2) | *Protobothrops kelomohy* | Thailand (Northern Thailand) | I.V. | 0.67 (0.58-0.78) μg/g | Malayan pit viper antivenin | Queen Saovabha Memorial Institute, Thailand | 2 | Not effective | Not effective | Not reported | Not reported |
| Wong KY, 2021 (3) | *Naja naja* | Sri Lanka (Colombo) | I.V. | 0.75 (0.48-1.18) μg/g | Snake Venom Antiserum I.P. (Asia) | VINS Bioproducts Ltd., India | 2.5 | 35.00 | 1.23 (1.11-1.37) | Not reported | 0.74 |
| Faisal T, 2021 (4) | *Daboia russelii* | Sri Lanka | I.V. | 0.24 (0.22-0.39) μg/g | Snake Venom Antiserum I.P. (Asia) | VINS Bioproducts Ltd., India | 5 | 6.25 (5.04-7.75) | 4.61 (3.65-11.90) | 3.69 | 43.40 |
| Faisal T, 2021 (4) | *Daboia russelii* | India | I.V. | 0.32 (0.27-0.46) μg/g | Snake Venom Antiserum I.P. (Asia) | VINS Bioproducts Ltd., India | 5 | 7.83 (6.40-9.58) | 4.70 (3.97-6.76) | 3.76 | 44.30 |
| Attarde S, 2021 (5) | *Naja sagittifera* | India  (Andaman Island) | I.V. | 0.47 μg/g | Polyvalent snake antivenom (Asia) | Premium Serums & Vaccines Pvt. Ltd., India | 3 | Not effective | Not reported | Not effective | Not reported |
| Attarde S, 2021 (5) | *Naja sagittifera* | India  (Andaman Island) | I.V. | 0.47 μg/g | Polyvalent Snake Antivenom (Asia) | Bharat Serums & Vaccines, India | 3 | 126.00 (100.00-158.32) | Not reported | 0.15 | Not reported |
| Attarde S, 2021 (5) | *Naja sagittifera* | Andaman Island, India | I.V. | 0.47 μg/g | Cobra antivenin | Queen Saovabha Memorial Institute, Thailand | 3 | 136.00 (117.43-157.68) | Not reported | 0.14 | Not reported |
| Attarde S, 2021 (5) | *Naja naja* | India | I.V. | 0.84 μg/g | Polyvalent snake antivenom (Asia) | Premium Serums & Vaccines Pvt. Ltd., India | 5 | 151.74 (123.78-186.00) | Not reported | 0.44 | Not reported |
| Attarde S, 2021 (5) | *Naja naja* | India | I.V. | 0.84 μg/g | Polyvalent Snake Antivenom (Asia) | Bharat Serums & Vaccines, India | 5 | 198.46 (140.51-280.32) | Not reported | 0.34 | Not reported |
| Tan CH, 2021 (6) | *Naja philippinensis* | Philippines | I.V. | 0.18 (0.12-0.27) μg/g | Philippine Cobra antivenom | Research Institute for Tropical Medicine in the Philippines, Philippines | 5 | 44.94 (20.6-69.23) | 0.5 (0.33- 0.75) | 0.40 | 24.72 |
| Tan CH, 2021 (6) | *Naja samarensis* | Philippines | I.V. | 0.2 (0.16-0.25) μg/g | Philippine Cobra antivenom | Research Institute for Tropical Medicine in the Philippines, Philippines | 5 | 120.86 (104.79-139.40) | 0.21 (0.17-0.26) | 0.17 | 10.21 |
| Oh AMF, 2021 (7) | *Bungarus multicinctus* | China | I.V. | 0.027 (0.026-0.028) μg/g | Bungarus multicinctus monovalent antivenom | Shanghai Serum Biological Technology Co., Ltd., China | 5 | 1.65 | 1.88 (1.81-1.95) | 1.50 | 19.52 |
| Oh AMF, 2021 (7) | *Bungarus multicinctus* | Taiwan | I.V. | 0.087 (0.084-0.091) μg/g | Bungarus multicinctus monovalent antivenom | Shanghai Serum Biological Technology Co., Ltd., China | 5 | 4.13 | 2.49 (2.41-2.61) | 2.00 | 25.91 |
| Oh AMF, 2021 (7) | *Bungarus multicinctus* | China | I.V. | 0.027 (0.026-0.028) μg/g | Neuro bivalent antivenom | Centres for Disease Control, Taiwan | 5 | 8.92 | 0.35 (0.34-0.36) | 0.28 | 10.3 |
| Oh AMF, 2021 (7) | *Bungarus multicinctus* | Taiwan | I.V. | 0.087 (0.084-0.091) μg/g | Neuro bivalent antivenom | Centres for Disease Control, Taiwan | 5 | 33.39 | 0.31 (0.30-0.32) | 0.25 | 9.15 |
| Laxme RRS, 2021 (8) | *Daboia russelii* | India (Punjab, North India) | I.V. | 2.97 (2.46 – 3.58) μg/mouse | Polyvalent snake antivenom (Asia) | Premium Serums & Vaccines Pvt. Ltd., India | 5 | 29.99 (24.99-36) | Not reported | 0.40 | Not reported |
| Laxme RRS, 2021 (8) | *Daboia russelii* | India (Andhra Pradesh, Southeast India) | I.V. | 3.65 (3.37-3.95) μg/mouse | Polyvalent snake antivenom (Asia) | Premium Serums & Vaccines Pvt. Ltd., India | 5 | 16.07 (13.39-19.30) | Not reported | 0.91 | Not reported |
| Laxme RRS, 2021 (8) | *Daboia russelii* | India (Wast Bengal, East India) | I.V. | 6.9 (6.23-7.62) μg/mouse | Polyvalent snake antivenom (Asia) | Premium Serums & Vaccines Pvt. Ltd., India | 5 | 27.66 (20.47-37.37) | Not reported | 1.00 | Not reported |
| Laxme RRS, 2021 (8) | *Daboia russelii* | India (Maharashtra, Southwest India) | I.V. | 3.8 (3.42-4.21) μg/mouse | Polyvalent snake antivenom (Asia) | Premium Serums & Vaccines Pvt. Ltd., India | 5 | 17.93 (15.52-20.71) | Not reported | 0.85 | Not reported |
| Laxme RRS, 2021 (8) | *Daboia russelii* | India (Madhya Pradesh, Central India) | I.V. | 2.29 (2.11-2.48) μg/mouse | Polyvalent snake antivenom (Asia) | Premium Serums & Vaccines Pvt. Ltd., India | 5 | 10.71 (8.92-12.86) | Not reported | 0.86 | Not reported |
| Laxme RRS, 2021 (9) | *Naja naja* | India (Punjab, North India) | I.V. | 6.53 (5.65-7.54) μg/mouse | Polyvalent snake antivenom (Asia) | Premium Serums & Vaccines Pvt. Ltd., India | 5 | 67.37 | Not reported | 0.39 | Not reported |
| Laxme RRS, 2021 (9) | *Naja naja* | India (Andhra Pradesh, Southeast India) | I.V. | 10.93 (9.10-13.10) μg/mouse | Polyvalent snake antivenom (Asia) | Premium Serums & Vaccines Pvt. Ltd., India | 5 | 54.24 | Not reported | 0.81 | Not reported |
| Laxme RRS, 2021 (9) | *Naja naja* | India (Wast Bengal, East India) | I.V. | 5.46 (4.56-6.53) μg/mouse | Polyvalent snake antivenom (Asia) | Premium Serums & Vaccines Pvt. Ltd., India | 5 | 60.45 | Not reported | 0.36 | Not reported |
| Laxme RRS, 2021 (9) | *Naja naja* | India (Maharashtra, Southwest India) | I.V. | 50.63 μg/mouse | Polyvalent snake antivenom (Asia) | Premium Serums & Vaccines Pvt. Ltd., India | 5 | Not effective | Not reported | Not effective | Not reported |
| Laxme RRS, 2021 (9) | *Naja naja* | India (Madhya Pradesh, Central India) | I.V. | 4.36 (3.76-5.04) μg/mouse | Polyvalent snake antivenom (Asia) | Premium Serums & Vaccines Pvt. Ltd., India | 5 | 60.45 | Not reported | 0.29 | Not reported |
| Yee KT, 2020 (10) | *Trimeresurus erythrurus* | Myanmar | I.V. | 93.76 (74.64-116.68) μg/mouse | Green pit viper antivenin | Queen Saovabha Memorial Institute, Thailand | 3 | 71 | Not reported | 2.64 | Not reported |
| Yee KT, 2020 (10) | *Trimeresurus erythrurus* | Myanmar | I.V. | 93.76 (74.64-116.68) μg/mouse | Russell’ viper anti-venom | Myanmar Pharmaceutical Factory, Myanmar | 3.5 | 125 | Not reported | 1.88 | Not reported |
| Tan KY, 2020 (11) | *Ophiophagus hannah* | Malaysia (Seremban) | I.V. | 0.9 (0.59-1.36) μg/g | King cobra antivenin | Queen Saovabha Memorial Institute, Thailand | 5 | 129.09 (111.99-148.94) | 0.77 (0.50-1.16) | 0.61 | 30.96 |
| Tan KY, 2020 (11) | *Ophiophagus hannah* | Thailand (Bangkok) | I.V. | 1.04 (0.88-1.23) μg/g | King cobra antivenin | Queen Saovabha Memorial Institute, Thailand | 5 | 39.37 (35.69-43.43) | 2.91 (2.46-3.44) | 2.32 | 117.77 |
| Tan KY, 2020 (11) | *Ophiophagus hannah* | China (Guangzhou) | I.V. | 0.51 (0.44-0.60) μg/g | King cobra antivenin | Queen Saovabha Memorial Institute, Thailand | 5 | 170.16 (153.72-188.37) | 0.33 (0.28-0.39) | 0.26 | 13.19 |
| Tan KY, 2020 (11) | *Ophiophagus hannah* | Indonesia (East Java Island) | I.V. | 0.48 (0.38-0.59 μg/g | King cobra antivenin | Queen Saovabha Memorial Institute, Thailand | 5 | 139.58 (104.98-185.53) | 0.38 (0.30-0.47) | 0.3 | 15.23 |
| Tan KY, 2020 (11) | *Ophiophagus hannah* | Malaysia (Seremban) | I.V. | 0.90 (0.59-1.36) μg/g | Serum Anti Bisa Ular (Biosave) | Bio Farma, Indonesia | 2.5 | 97.24 (79.44-119.03) | 0.53 (0.35-0.80) | 0.32 | 3.11 |
| Tan KY, 2020 (11) | *Ophiophagus hannah* | Thailand (Bangkok) | I.V. | 1.04 (0.88-1.23) μg/g | Serum Anti Bisa Ular (Biosave) | Bio Farma, Indonesia | 5 | 45.30 (43.65-47.02) | 2.53 (2.14-2.99) | 2.02 | 19.63 |
| Tan KY, 2020 (11) | *Ophiophagus hannah* | China (Guangzhou) | I.V. | 0.51 (0.44-0.60) μg/g | Serum Anti Bisa Ular (Biosave) | Bio Farma, Indonesia | 2.5 | 73.45 (64.53-83.61) | 0.40 (0.34-0.47) | 0.24 | 2.33 |
| Tan KY, 2020 (11) | *Ophiophagus hannah* | Indonesia (East Java Island) | I.V. | 0.48 (0.38-0.59) μg/g | Serum Anti Bisa Ular (Biosave) | Bio Farma, Indonesia | 5 | 148.41 (139.14-158.30) | 0.36 (0.28-0.44) | 0.29 | 2.81 |
| Tan KY, 2020 (11) | *Ophiophagus hannah* | Malaysia (Seremban) | I.V. | 0.90 (0.59-1.36 μg/g) | Naja atra antivenom | Shanghai Institute Biological Technology Co., Ltd., China | 2.5 | >200 | - | Not effective | - |
| Tan KY, 2020 (11) | *Ophiophagus hannah* | Thailand (Bangkok) | I.V. | 1.04 (0.88-1.23) μg/g | Naja atra antivenom | Shanghai Institute Biological Technology Co., Ltd., China | 5 | 97.24 (79.44-119.03) | 1.34 (1.13-1.58) | 1.07 | 4.19 |
| Tan KY, 2020 (11) | *Ophiophagus hannah* | China (Guangzhou) | I.V. | 0.51 (0.44-0.60) μg/g | Naja atra antivenom | Shanghai Institute Biological Technology Co., Ltd., China | 2.5 | >200 | - | Not effective | - |
| Tan KY, 2020 (11) | *Ophiophagus hannah* | Indonesia (East Java Island) | I.V. | 0.48 (0.38-0.59) μg/g | Naja atra antivenom | Shanghai Institute Biological Technology Co., Ltd., China | 2.5 | >200 | - | Not effective | - |
| Lin B, 2020 (12) | *Bungarus multicinctus* | China | I.P. | 0.09 μg/g | Bungarus multicinctus monovalent antivenom | Shanghai Serum Biological Technology Co., Ltd., China | 3 | 17.68 μg/g | Not reported | Not reported | Not reported |
| Lin B, 2020 (12) | *Bungarus fasciatus* | China | I.P. | 1.50 μg/g | Bungarus multicinctus monovalent antivenom | Shanghai Serum Biological Technology Co., Ltd., China | 3 | >800 μg/g | Not reported | Not reported | Not reported |
| Lin B, 2020 (12) | *Naja atra* | China | I.P. | 0.50 μg/g | Bungarus multicinctus monovalent antivenom | Shanghai Serum Biological Technology Co., Ltd., China | 3 | >800 μg/g | Not reported | Not reported | Not reported |
| Lin B, 2020 (12) | *Ophiophagus hannah* | China | I.P. | 0.44 μg/g | Bungarus multicinctus monovalent antivenom | Shanghai Serum Biological Technology Co., Ltd., China | 3 | 499 μg/g | Not reported | Not reported | Not reported |
| Liew JL, 2020 (13) | *Trimeresurus purpureomaculatus* | Malaysia | I.V. | 0.89 (0.59-1.36) μg/g | Green pit viper antivenin | Queen Saovabha Memorial Institute, Thailand | 5 | 35 | 2.54 | 2.03 | 100.5 |
| Liew JL, 2020 (13) | *Trimeresurus albolabris* | Thailand | I.V. | 0.50 (0.40-0.63) μg/g | Green pit viper antivenin | Queen Saovabha Memorial Institute, Thailand | 5 | 10.95 | 5.73 | 4.59 | 227.23 |
| Lee LP, 2020 (14) | *Trimeresurus wiroti* | Malaysia | I.V. | 0.78 (0.64-0.96) μg/g | Green pit viper antivenin | Queen Saovabha Memorial Institute, Thailand | 2.5 | 22.47 (14.80-34.11) | Not reported | 1.05 | Not reported |
| Lee LP, 2020 (14) | *Trimeresurus puniceus* | Indonesia | I.V. | 1.21 (1.05-1.39) μg/g | Green pit viper antivenin | Queen Saovabha Memorial Institute, Thailand | 2.5 | 45.62 (29.15-71.40) | Not reported | 0.79 | Not reported |
| Hia YL, 2020 (15) | *Bungarus fasciatus* | Malaysia (Peninsular) | I.V. | 0.91 (0.54-1.52) μg/g | Banded krait antivenin | Queen Saovabha Memorial Institute, Thailand | 2.5 | 150 | 0.32 (0.19-0.53) | 0.19 | 3.24 |
| Hia YL, 2020 (15) | *Bungarus fasciatus* | Thailand (Bangkok) | I.V. | 2.55 (2.27-2.86) μg/g | Banded krait antivenin | Queen Saovabha Memorial Institute, Thailand | 2.5 | 50 | 2.68 (2.91-3.00) | 1.61 | 27.43 |
| Hia YL, 2020 (15) | *Bungarus fasciatus* | Indonesia (Java Island) | I.V. | 0.45 (0.30-0.68) μg/g | Banded krait antivenin | Queen Saovabha Memorial Institute, Thailand | 2.5 | 15.80 | 1.50 (1.00-2.26) | 0.9 | 15.33 |
| Hia YL, 2020 (15) | *Bungarus fasciatus* | Myanmar | I.V. | 2.44 (2.15-2.78) μg/g | Banded krait antivenin | Queen Saovabha Memorial Institute, Thailand | 2.5 | 77.80 | 1.65 (1.45-1.88) | 0.99 | 16.87 |
| Hia YL, 2020 (15) | *Bungarus fasciatus* | China (Guangdong) | I.V. | 1.44 (1.15-1.81) μg/g | Banded krait antivenin | Queen Saovabha Memorial Institute, Thailand | 2.5 | 100 | 0.76 (0.60-0.95) | 0.45 | 7.67 |
| Choraria A, 2020 (16) | *Daboia russelii* | India | I.V. | 10 μg/mouse | Polyvalent Snake Antivenom (Asia) | Bharat Serums & Vaccines, India | 3 | 35 (29.34-40.65) | Not reported | 0.57 | Not reported |
| Choraria A, 2020 (16) | *Echis carinatus* | India | I.V. | 12 μg/mouse | Polyvalent Snake Antivenom (Asia) | Bharat Serums & Vaccines, India | 3 | 40.80 (35.34-46.25) | Not reported | 0.60 | Not reported |
| Tan CH, 2019 (17) | *Trimeresurus nebularis* | Malaysia | I.V. | 2.00 (1.61-2.48) μg/g | Green pit viper antivenin | Queen Saovabha Memorial Institute, Thailand | 5 | 100 | 2.00 (1.61-2.48) | 1.60 | 79.2 |
| Pla D, 2019 (18) | *Daboia russelii* | Sri Lanka | I.V. | 7.89 (7.16-10.90) μg/mouse | Snake Venom Antiserum I.P. (Asia) | VINS Bioproducts Ltd., India | 3 | Not reported | 1.89 (1.49-2.84) | 1.26 | Not reported |
| Pla D, 2019 (18) | *Daboia russelii* | Sri Lanka | I.V. | 7.89 (7.16-10.90) μg/mouse | Polyvalent snake antivenom (Asia) | Premium Serums & Vaccines Pvt Ltd., India | 3 | Not reported | 2.33 (1.62-5.12) | 1.55 | Not reported |
| Pla D, 2019 (18) | *Daboia russelii* | Pakistan | I.V. | 3.67 (3.01-4.37) μg/mouse | Snake Venom Antiserum I.P. (Asia) | VINS Bioproducts Ltd., India | 3 | Not reported | 1.86 (1.14-3.66) | 1.24 | Not reported |
| Pla D, 2019 (18) | *Daboia russelii* | Pakistan | I.V. | 3.67 (3.01-4.37) μg/mouse | Polyvalent snake antivenom (Asia) | Premium Serums & Vaccines Pvt Ltd., India | 3 | Not reported | 2.66 (1.73-5.48) | 1.78 | Not reported |
| Pla D, 2019 (18) | *Daboia russelii* | Bangladesh | I.V. | 3.69 (2.00-5.86) μg/mouse | Snake Venom Antiserum I.P. (Asia) | VINS Bioproducts Ltd., India | 3 | Not reported | <1.50 | < 1.00 | Not reported |
| Pla D, 2019 (18) | *Daboia russelii* | Bangladesh | I.V. | 3.69 (2.00-5.86) μg/mouse | Polyvalent snake antivenom (Asia) | Premium Serums & Vaccines Pvt Ltd., India | 3 | Not reported | <1.50 | < 1.00 | Not reported |
| Oh AMF, 2019 (19) | *Bungarus sindanus* | Pakistan | I.V. | 0.04 (0.035-0.045) μg/g | Snake Venom Antiserum I.P. (Asia) | VINS Bioproducts Ltd., India | 5 | 13.29 | 0.32 (0.24-0.41) | 0.25 | 3.05 |
| Lingam TΜC, 2019 (20) | *Daboia siamensis* | Thailand | I.V. | 0.34 (0.30-0.38) μg/g | Russell's viper antivenin | Queen Saovabha Memorial Institute, Thailand | 5 | 9.5 | 3.75 (3.31-4.20) | 3.01 | 74.32 |
| Lingam TΜC, 2019 (20) | *Daboia siamensis* | Indonesia | I.V. | 0.22 (0.20-0.24) μg/g | Russell's viper antivenin | Queen Saovabha Memorial Institute, Thailand | 5 | 6.64 | 3.48 (3.16-3.80) | 2.78 | 68.64 |
| Lingam TΜC, 2019 (20) | *Daboia siamensis* | Thailand | I.V. | 0.34 (0.30-0.38) μg/g | Serum Anti Bisa Ular (Biosave) | Bio Farma, Indonesia | 5 | Not effective | Not reported | Not reported | Not reported |
| Lingam TΜC, 2019 (20) | *Daboia siamensis* | Indonesia | I.V. | 0.22 (0.20-0.24) μg/g | Serum Anti Bisa Ular (Biosave) | Bio Farma, Indonesia | 5 | Not effective | Not reported | Not reported | Not reported |
| Laxme RRS, 2019 (21) | *Naja naja* | India (Maharashtra, West India) | I.V. | 0.73 (0.50-0.88) μg/g | Polyvalent snake antivenom (Asia) | Premium Serums & Vaccines Pvt. Ltd., India | 5 | 81.27 (67.30-98.13) | Not reported | 0.72 | Not reported |
| Laxme RRS, 2019 (21) | *Naja kaouthia* | India (Arunachal Pradesh, Northeast India) | I.V. | 0.24 (0.18-0.28) μg/g | Polyvalent snake antivenom (Asia) | Premium Serums & Vaccines Pvt. Ltd., India | 5 | 122.04 (101.1-147.32) | Not reported | 0.16 | Not reported |
| Laxme RRS, 2019 (21) | *Naja kaouthia* | India (West Bengal, East India) | I.V. | 1.23 (1.14-1.33) μg/g | Polyvalent snake antivenom (Asia) | Premium Serums & Vaccines Pvt. Ltd., India | 5 | Not effective | Not reported | Not effective | Not reported |
| Laxme RRS, 2019 (21) | *Bungarus caeruleus* | India (Punjab, North India) | I.V. | 0.10 (0.03-0.31) μg/g | Polyvalent snake antivenom (Asia) | Premium Serums & Vaccines Pvt. Ltd., India | 5 | 26.17 (19.36-35.37) | Not reported | 0.31 | Not reported |
| Laxme RRS, 2019 (21) | *Bungarus sindanus* | India (Rajasthan, Northwest India) | I.V. | 0.02 (0.01-0.03) μg/g | Polyvalent snake antivenom (Asia) | Premium Serums & Vaccines Pvt. Ltd., India | 5 | 5.43 (4.34-6.51) | Not reported | 0.27 | Not reported |
| Laxme RRS, 2019 (21) | *Bungarus fasciatus* | India (West Bengal, East India) | I.V. | 1.12 (0.93-1.33) μg/g | Polyvalent snake antivenom (Asia) | Premium Serums & Vaccines Pvt. Ltd., India | 5 | 138.89 (111.11-166.67) | Not reported | 0.64 | Not reported |
| Laxme RRS, 2019 (21) | *Echis carinatus* | India (Maharashtra, West India) | I.V. | 0.61 (0.34-0.75) μg/g | Polyvalent snake antivenom (Asia) | Premium Serums & Vaccines Pvt. Ltd., India | 5 | 92.54 (73.96-111.11) | Not reported | 0.53 | Not reported |
| Laxme RRS, 2019 (21) | *Echis carinatus sochureki* | India (Rajasthan, Northwest India) | I.V. | 1.76 (0.77-2.10) μg/g | Polyvalent snake antivenom (Asia) | Premium Serums & Vaccines Pvt. Ltd., India | 5 | 92.54 (73.96-111.11) | Not reported | 1.51 | Not reported |
| Deka A, 2019 (22) | *Naja kaouthia* | India | Not reported | 0.148 μg/g | Polyvalent Snake Antivenom (Asia) | Bharat Serums & Vaccines, India | 4 | 92.68 ± 4.68 mg/g | Not reported | Not reported | Not reported |
| Deka A, 2019 (22) | *Naja kaouthia* | India | Not reported | 0.148 μg/g | Snake Venom Antiserum I.P. (Asia) | VINS Bioproducts Ltd., India | 4 | 76.38 ± 3.48 mg/g | Not reported | Not reported | Not reported |
| Deka A, 2019 (22) | *Naja kaouthia* | India | Not reported | 0.148 μg/g | Snake antivenin I.P. (Asia) | Haffkine Biopharmaceutical Corporation Limited, India | 4 | 112.66 ± 5.11 mg/g | Not reported | Not reported | Not reported |
| Deka A, 2019 (22) | *Naja kaouthia* | Bangladesh | Not reported | 0.12 μg/g | Polyvalent Snake Antivenom (Asia) | Bharat Serums & Vaccines, India | 4 | 97.28 ± 2.46 mg/g | Not reported | Not reported | Not reported |
| Deka A, 2019 (22) | *Naja kaouthia* | Bangladesh | Not reported | 0.12 μg/g | Snake Venom Antiserum I.P. (Asia) | VINS Bioproducts Ltd., India | 4 | 94.62 ± 4.52 mg/g | Not reported | Not reported | Not reported |
| Deka A, 2019 (22) | *Naja kaouthia* | Bangladesh | Not reported | 0.12 μg/g | Snake antivenin I.P. (Asia) | Haffkine Biopharmaceutical Corporation Limited, India | 4 | 137.23 ± 4.42 mg/g | Not reported | Not reported | Not reported |
| Chaisakul J, 2019 (23) | *Daboia siamensis* | Thailand | I.V. | 10.40 (5.61-19.26) μg/mouse | Russell's viper antivenin | Queen Saovabha Memorial Institute, Thailand | 6 | 17 (10.88-26.56) | Not reported | Not reported | Not reported |
| Chaisakul J, 2019 (23) | *Daboia siamensis* | Myanmar | I.V. | 6.00 (3.26-11.04) μg/mouse | Russell's viper antivenin | Queen Saovabha Memorial Institute, Thailand | 6 | 60 (40.58-88.70) | Not reported | Not reported | Not reported |
| Chaisakul J, 2019 (23) | *Daboia siamensis* | Taiwan | I.V. | 6.70 (3.26-13.77) μg/mouse | Russell's viper antivenin | Queen Saovabha Memorial Institute, Thailand | 6 | 29.16 (23.75-35.79) | Not reported | Not reported | Not reported |
| Chaisakul J, 2019 (23) | *Daboia siamensis* | China | I.V. | 4.89 (4.11-5.83) μg/mouse | Russell's viper antivenin | Queen Saovabha Memorial Institute, Thailand | 6 | 49.99 (47.23-52.90) | Not reported | Not reported | Not reported |
| Tan CH, 2018 (24) | *Hydrophis curtus* | Malaysia | I.V. | 0.20 (0.18-0.24) μg/g | Sea Snake Antivenom | CSL Ltd., Australia | 5 | 9.87 (7.98-12.21) | 2.53 (0.28-3.04) | 2.03 | 9.35 |
| Tan KY, 2018 (25) | *Daboia siamensis* | China (Guangxi) | I.V. | 0.18 (0.12-0.27) μg/g | Doboia siamensis Monovalent Snake Antivenom | Centres for Disease Control, Taiwan | 5 | 11.24 | 1.76 (1.17-2.64) | 1.41 | 73.1 |
| Tan KY, 2018 (25) | *Daboia siamensis* | Taiwan | I.V. | 0.09 (0.06-0.14) μg/g | Doboia siamensis Monovalent Snake Antivenom | Centres for Disease Control, Taiwan | 5 | 4.9 | 2.02 (1.35-3.14) | 1.62 | 83.9 |
| Tan KY, 2018 (25) | *Daboia siamensis* | China (Guangxi) | I.V. | 0.18 (0.12-0.27) μg/g | Gloydius brevicaudus Monovalent Snake Antivenom | Shanghai Serum Biological Technology Co., Ltd., China | 5 | 91.24 | 0.22 (0.14-0.33) | 0.17 | 1 |
| Tan KY, 2018 (25) | *Daboia siamensis* | China (Guangxi) | I.V. | 0.18 (0.12-0.27) μg/g | Deinagkistrodon acutus Monovalent Snake Antivenom | Shanghai Serum Biological Technology Co., Ltd., China | 5 | Not effective | Not effective | Not effective | Not effective |
| Sanz L, 2018 (26) | *Daboia siamensis* | Taiwan | I.P. | 0.47 μg/g | Doboia siamensis Monovalent Snake Antivenom | Centres for Disease Control, Taiwan | 8 | 10.27 (7.95-12.67)  mg /g | Not reported | Not reported | Not reported |
| Liu BS, 2018 (27) | *Naja atra* | Taiwan | I.P. | 0.67 μg/g | Neuro bivalent antivenom | Centres for Disease Control, Taiwan | 5 | 101.82 (86.97-119.17) mg/g | Not reported | Not reported | 81.5 |
| Liu BS, 2018 (27) | *Naja atra* | Taiwan | I.P. | 0.67 μg/g | SAV-Naja | Institute of Vaccines and Biological Substances (IVAC), Vietnam | 5 | 17.41 (14.87-20.38) mg/g | Not reported | Not reported | 13.9 |
| Liu BS, 2018 (27) | *Naja atra* | Taiwan | I.P. | 0.67 μg/g | Neuro polyvalent snake antivenom | Queen Saovabha Memorial Institute, Thailand | 5 | 9.70 (9.28-11.35) mg/g | Not reported | Not reported | 7.8 |
| Liu BS, 2018 (27) | *Naja atra* | Taiwan | I.P. | 0.67 μg/g | Doboia siamensis Monovalent Snake Antivenom | Centres for Disease Control, Taiwan | 5 | Not effective | Not reported | Not reported | Not effective |
| Faisal T, 2018 (28) | *Daboia russelii* | Pakistan | I.V. | 0.19 (0.17-0.25) μg/g | Snake Venom Antiserum I.P. (Asia) | VINS Bioproducts Ltd., India | 5 | 78.29 (63.98-95.80) | 0.29 (0.26-0.31) | 0.23 | 2.7 |
| Tan CH, 2017 (29) | *Trimeresurus insularis* | Indonesia | I.V. | 0.78 (0.64-0.96) μg/g | Serum Anti Bisa Ular (Biosave) | Bio Farma, Indonesia | 2.5 | 145.90 (129.12-164.97) | 0.27 (0.11-0.33) | 0.16 | 1.54 |
| Tan CH, 2017 (29) | *Trimeresurus purpureomaculatus* | Indonesia | I.V. | 0.70 (0.65-0.76) μg/g | Serum Anti Bisa Ular (Biosave) | Bio Farma, Indonesia | 2.5 | 100 (80.68-123.94) | 0.35 (0.34-0.35) | 0.21 | 2.01 |
| Tan CH, 2017 (29) | *Trimeresurus hageni* | Indonesia | I.V. | 0.50 (0.40-0.63) μg/g | Serum Anti Bisa Ular (Biosave) | Bio Farma, Indonesia | 2.5 | 70.68 (54.11-92.31) | 0.35 (0.28-0.45) | 0.21 | 2.03 |
| Tan CH, 2017 (29) | *Trimeresurus puniceus* | Indonesia | I.V. | 1.10 (0.73-1.69) μg/g | Serum Anti Bisa Ular (Biosave) | Bio Farma, Indonesia | 2.5 | 141 (104.04-191.2) | 0.39 (0.25-0.60) | 0.23 | 2.24 |
| Tan CH, 2017 (29) | *Trimeresurus insularis* | Indonesia | I.V. | 0.78 (0.64-0.96) μg/g | Green pit viper antivenin | Queen Saovabha Memorial Institute, Thailand | 2.5 | 13.78 (8.70-21.80) | 2.83 (1.23-3.48) | 1.7 | 83.98 |
| Tan CH, 2017 (29) | *Trimeresurus purpureomaculatus* | Indonesia | I.V. | 0.70 (0.65-0.76) μg/g | Green pit viper antivenin | Queen Saovabha Memorial Institute, Thailand | 5 | 38.13 (28.03-51.86) | 1.84 (1.78-1.89) | 1.47 | 72.63 |
| Tan CH, 2017 (29) | *Trimeresurus hageni* | Indonesia | I.V. | 0.50 (0.40-0.63) μg/g | Green pit viper antivenin | Queen Saovabha Memorial Institute, Thailand | 2.5 | 8 (5.50-11.60) | 3.13 (2.50-3.94) | 1.88 | 92.73 |
| Tan CH, 2017 (29) | *Trimeresurus puniceus* | Indonesia | I.V. | 1.10 (0.73-1.69) μg/g | Green pit viper antivenin | Queen Saovabha Memorial Institute, Thailand | 2.5 | 55.63 (36.60-84.40) | 0.99 (0.66-1.52) | 0.59 | 29.34 |
| Tan CH, 2017 (30) | *Laticauda colubrina* | Indonesia | I.V. | 0.10 (0.08-0.12) μg/g | Sea Snake Antivenom | CSL Ltd., Australia | 5 | 8.84 (6.76-11.54) | 1.36 (1.09-1.63) | 1.09 | Not reported |
| Oh AMF, 2017 (31) | *Bungarus caeruleus* | Sri Lanka | I.V. | 0.06 (0.04-0.08) μg/g | Snake Venom Antiserum I.P. (Asia) | VINS Bioproducts Ltd., India | 5 | 9.44 | 0.56 (0.43-0.72) | 0.44 | 5.36 |
| Oh AMF, 2017 (31) | *Bungarus caeruleus* | India | I.V. | 0.10 (0.08-0.12) μg/g | Snake Venom Antiserum I.P. (Asia) | VINS Bioproducts Ltd., India | 5 | 17.14 | 0.60 (0.48-0.74) | 0.48 | 5.85 |
| Oh AMF, 2017 (31) | *Bungarus caeruleus* | Pakistan | I.V. | 0.06 (0.05-0.07) μg/g | Snake Venom Antiserum I.P. (Asia) | VINS Bioproducts Ltd., India | 5 | 16.53 | 0.37 (0.32-0.42) | 0.3 | 3.66 |
| Wong KY, 2016 (32) | *Naja naja* | Pakistan | I.V. | 0.22 (0.12-0.40) μg/g | Snake Venom Antiserum I.P. (Asia) | VINS Bioproducts Ltd., India | 5 | 32.77 | 0.77 (0.69-0.85) | 0.61 | Not reported |
| Wong KY, 2016 (32) | *Naja naja* | Pakistan | I.V. | 0.22 (0.12-0.40) μg/g | Cobra antivenin | Queen Saovabha Memorial Institute, Thailand | 5 | 18 | 1.39 (1.25-1.55) | 1.11 | Not reported |
| Wong KY, 2016 (32) | *Naja naja* | Pakistan | I.V. | 0.22 (0.12-0.40) μg/g | Neuro bivalent antivenom | Centres for Disease Control, Taiwan | 5 | 75 | 0.34 (0.32-0.35) | 0.27 | Not reported |
| Villalta M, 2016 (33) | *Daboia russelii* | Sri Lanka | I.V. | 7.80 (7.00-8.60) μg/mouse | Snake Venom Antiserum I.P. (Asia) | VINS Bioproducts Ltd., India | 3 | Not reported | 1.90 (1.50-2.80) | Not reported | Not reported |
| Villalta M, 2016 (33) | *Echis carinatus* | Sri Lanka | I.V. | 8.80 (6.50-11.40) μg/mouse | Snake Venom Antiserum I.P. (Asia) | VINS Bioproducts Ltd., India | 3 | Not reported | 0.80 (0.50-1.30) | Not reported | Not reported |
| Villalta M, 2016 (33) | *Hypnale hynale* | Sri Lanka | I.V. | 17.30 (15.60-19.40) μg/mouse | Snake Venom Antiserum I.P. (Asia) | VINS Bioproducts Ltd., India | 3 | Not reported | 0.60 (0.30-0.90) | Not reported | Not reported |
| Villalta M, 2016 (33) | *Naja naja* | Sri Lanka | I.V. | 22.60 (15.90-29.70) μg/mouse | Snake Venom Antiserum I.P. (Asia) | VINS Bioproducts Ltd., India | 3 | Not reported | 0.70 (0.60-0.90) | Not reported | Not reported |
| Tan KY, 2016 (34) | *Naja kaouthia* | Thailand | I.V. | 0.18 (0.12-0.27) μg/g | Cobra antivenin | Queen Saovabha Memorial Institute, Thailand | 5 | 18.75 | 1.15 (0.77-1.73) | 0.92 | 20.44 |
| Tan KY, 2016 (34) | *Naja kaouthia* | Thailand | I.V. | 0.18 (0.12-0.27) μg/g | Sea Snake Antivenom | CSL Ltd., Australia | 5 | 11.24 | 2.00 (1.33-3.00) | 1.60 | 7.37 |
| Tan CH, 2016 (35) | *Naja sputatrix* | Indonesia (Java Island) | I.V. | 0.90 (0.59-1.36) μg/g | Serum Anti Bisa Ular (Biosave) | Bio Farma, Indonesia | 2.5 | 111.25 | 0.51 (0.33-0.76) | 0.3 | 2.9 |
| Tan CH, 2016 (35) | *Bungarus fasciatus* | Indonesia (Java Island) | I.V. | 0.45 (0.30-0.68) μg/g | Serum Anti Bisa Ular (Biosave) | Bio Farma, Indonesia | 5 | 44.94 | 1.10 (0.73-1.66) | 0.88 | 8.5 |
| Tan CH, 2016 (35) | *Calloselasma rhodostoma* | Indonesia (Java Island) | I.V. | 1.35 (0.78-2.06) μg/g | Serum Anti Bisa Ular (Biosave) | Bio Farma, Indonesia | 5 | 9.38 | 15.83 (9.15-24.16) | 12.67 | 121.8 |
| Tan CH, 2016 (35) | *Naja sputatrix* | Indonesia (Java Island) | I.V. | 0.90 (0.59-1.36) μg/g | Neuro-polyvalent snake antivenom | Queen Saovabha Memorial Institute, Thailand | 2.5 | 50 | 1.04 (0.68-1.56) | 0.62 | 8.3 |
| Tan CH, 2016 (35) | *Bungarus fasciatus* | Indonesia (Java Island) | I.V. | 0.45 (0.30-0.68) μg/g | Neuro-polyvalent snake antivenom | Queen Saovabha Memorial Institute, Thailand | 5 | 43.75 | 1.08 (0.72-1.63) | 0.86 | 11.5 |
| Tan CH, 2016 (35) | *Calloselasma rhodostoma* | Indonesia (Java Island) | I.V. | 1.35 (0.78-2.06) μg/g | Haemato-polyvalent snake antivenom | Queen Saovabha Memorial Institute, Thailand | 5 | 18.75 | 7.92 (4.58-12.09) | 6.34 | 147.5 |
| Tan CH, 2016 (35) | *Naja sumatrana* | Indonesia (Sumatra) | I.V. | 0.39 (0.32-0.48) μg/g | Serum Anti Bisa Ular (Biosave) | Bio Farma, Indonesia | 5 | 156.57 | 0.24 (0.19-0.29) | 0.19 | 1.8 |
| Tan CH, 2016 (35) | *Bungarus candidus* | Indonesia (Java Island) | I.V. | 0.11 (0.07-0.17) μg/g | Serum Anti Bisa Ular (Biosave) | Bio Farma, Indonesia | 5 | 111.25 | 0.12 (0.00-0.19) | 0.10 | 1 |
| Tan CH, 2016 (35) | *Naja sumatrana* | Indonesia (Sumatra) | I.V. | 0.39 (0.32-0.48) μg/g | Neuro-polyvalent snake antivenom | Queen Saovabha Memorial Institute, Thailand | 5 | 55.63 | 0.67 (0.55-0.82) | 0.53 | 7.1 |
| Tan CH, 2016 (35) | *Bungarus candidus* | Indonesia (Java Island) | I.V. | 0.11 (0.07-0.17) μg/g | Neuro-polyvalent snake antivenom | Queen Saovabha Memorial Institute, Thailand | 5 | 5.56 | 2.18 (1.39-3.36) | 1.74 | 23.2 |
| Tan CH, 2016 (36) | *Hydrophis schistosus* | Malaysia | I.V. | 0.07 (0.05-0.09) μg/g | Sea Snake Antivenom | CSL Ltd., Australia | 5 | 13.91 | 0.60 (0.43-0.78) | 0.48 | Not reported |
| Maduwage K, 2016 (37) | *Daboia russelii* | Sri Lanka | I.V. | 0.10 (0.08-0.12) μg/g | Snake Venom Antiserum I.P. (Asia) | VINS Bioproducts Ltd., India | 5 | Not reported | 2.06 | Not reported | Not reported |
| Maduwage K, 2016 (37) | *Echis carinatus* | Sri Lanka | I.V. | 0.66 (0.52-0.81) μg/g | Snake Venom Antiserum I.P. (Asia) | VINS Bioproducts Ltd., India | 5 | Not reported | 2.79 | Not reported | Not reported |
| Maduwage K, 2016 (37) | *Naja naja* | Sri Lanka | I.V. | 0.66 (0.48-0.98) μg/g | Snake Venom Antiserum I.P. (Asia) | VINS Bioproducts Ltd., India | 5 | Not reported | 4.32 | Not reported | Not reported |
| Maduwage K, 2016 (37) | *Bungarus caeruleus* | Sri Lanka | I.V. | 0.20 (0.15-0.25) μg/g | Snake Venom Antiserum I.P. (Asia) | VINS Bioproducts Ltd., India | 5 | Not reported | 3.92 | Not reported | Not reported |
| Maduwage K, 2016 (37) | *Daboia russelii* | Sri Lanka | I.V. | 0.10 (0.08-0.12) μg/g | Polyvalent Snake Antivenom (Asia) | Bharat Serums & Vaccines, India | 5 | Not reported | 1.24 | Not reported | Not reported |
| Maduwage K, 2016 (37) | *Echis carinatus* | Sri Lanka | I.V. | 0.66 (0.52-0.81) μg/g | Polyvalent Snake Antivenom (Asia) | Bharat Serums & Vaccines, India | 5 | Not reported | 2.82 | Not reported | Not reported |
| Maduwage K, 2016 (37) | *Naja naja* | Sri Lanka | I.V. | 0.66 (0.48-0.98) μg/g | Polyvalent Snake Antivenom (Asia) | Bharat Serums & Vaccines, India | 5 | Not reported | 2.42 | Not reported | Not reported |
| Maduwage K, 2016 (37) | *Bungarus caeruleus* | Sri Lanka | I.V. | 0.20 (0.15-0.25) μg/g | Polyvalent Snake Antivenom (Asia) | Bharat Serums & Vaccines, India | 5 | Not reported | 2.93 | Not reported | Not reported |
| Yap MK, 2015 (38) | *Naja sputatrix* | Not reported (Studies from Malaysia) | I.V. | 0.90 (0.59-1.36) μg/g | Neuro-polyvalent snake antivenom | Queen Saovabha Memorial Institute, Thailand | 2.5 | 136.72 | Not reported | Not reported | Not reported |
| Tan KY, 2015 (39) | *Naja kaouthia* | Malaysia | I.V. | 0.90 (0.59-1.36) μg/g | Cobra antivenin | Queen Saovabha Memorial Institute, Thailand | 5 | 78.29 | 1.38 (0.90-2.08) | 1.10 | Not reported |
| Tan KY, 2015 (39) | *Naja kaouthia* | Thailand | I.V. | 0.18 (0.12-0.27) μg/g | Cobra antivenin | Queen Saovabha Memorial Institute, Thailand | 5 | 18.75 | 1.15 (0.77-1.73) | 0.92 | Not reported |
| Tan KY, 2015 (39) | *Naja kaouthia* | Vietnam | I.V. | 0.90 (0.59-1.36) μg/g | Cobra antivenin | Queen Saovabha Memorial Institute, Thailand | 5 | 120.86 | 0.87 (0.57-1.32) | 0.70 | Not reported |
| Tan KY, 2015 (39) | *Naja kaouthia* | Malaysia | I.V. | 0.90 (0.59-1.36) μg/g | Neuro-polyvalent snake antivenom | Queen Saovabha Memorial Institute, Thailand | 5 | 70.68 | 1.43 (0.94-2.16) | 1.14 | Not reported |
| Tan KY, 2015 (39) | *Naja kaouthia* | Thailand | I.V. | 0.18 (0.12-0.27) μg/g | Neuro-polyvalent snake antivenom | Queen Saovabha Memorial Institute, Thailand | 5 | 17.67 | 1.17 (0.78-1.76) | 0.94 | Not reported |
| Tan KY, 2015 (39) | *Naja kaouthia* | Vietnam | I.V. | 0.90 (0.59-1.36) μg/g | Neuro-polyvalent snake antivenom | Queen Saovabha Memorial Institute, Thailand | 5 | 89.89 | 1.10 (0.72-1.66) | 0.88 | Not reported |
| Tan CH, 2015 (40) | *Hydrophis schistosus* | Malaysia | I.V. | 0.07 (0.05-0.09) μg/g | Neuro bivalent antivenom | Centres for Disease Control, Taiwan | 2.5 | 141.36 | 0.03 (0.02-0.04) | 0.02 | Not reported |
| Tan CH, 2015 (40) | *Hydrophis curtus* | Malaysia | I.V. | 0.11 (0.07-0.17) μg/g | Neuro bivalent antivenom | Centres for Disease Control, Taiwan | 2.5 | 200 | 0.03 (0.02-0.05) | 0.02 | Not reported |
| Tan CH, 2015 (40) | *Hydrophis schistosus* | Malaysia | I.V. | 0.07 (0.05-0.09) μg/g | Cobra antivenin | Queen Saovabha Memorial Institute, Thailand | 2.5 | 89.89 | 0.05 (0.03-0.06) | 0.03 | Not reported |
| Tan CH, 2015 (40) | *Hydrophis curtus* | Malaysia | I.V. | 0.11 (0.07-0.17) μg/g | Cobra antivenin | Queen Saovabha Memorial Institute, Thailand | 2.5 | 89.89 | 0.07 (0.05-0.11) | 0.04 | Not reported |
| Tan CH, 2015 (40) | *Hydrophis schistosus* | Malaysia | I.V. | 0.07 (0.05-0.09) μg/g | Neuro-polyvalent snake antivenom | Queen Saovabha Memorial Institute, Thailand | 5 | 100 | 0.07 (0.05-1.00) | 0.06 | Not reported |
| Tan CH, 2015 (40) | *Hydrophis curtus* | Malaysia | I.V. | 0.11 (0.07-0.17) μg/g | Neuro-polyvalent snake antivenom | Queen Saovabha Memorial Institute, Thailand | 5 | 125 | 0.09 (0.06-0.14) | 0.07 | Not reported |
| Leong PK, 2015 (41) | *Naja sumatrana* | Malaysia | I.V. | 0.50 (0.40-0.62) μg/g | Neuro-polyvalent snake antivenom | Queen Saovabha Memorial Institute, Thailand | 5 | 25 | 2.30 (1.86-2.85) | 1.84 | Not reported |
| Leong PK, 2015 (41) | *Naja kaouthia* | Thailand | I.V. | 0.23 (0.15-0.34) μg/g | Neuro-polyvalent snake antivenom | Queen Saovabha Memorial Institute, Thailand | 5 | 22.47 | 1.18 (0.78-1.79) | 0.94 | Not reported |
| Leong PK, 2015 (41) | *Naja sputatrix* | Indonesia | I.V. | 0.90 (0.59-1.36) μg/g | Neuro-polyvalent snake antivenom | Queen Saovabha Memorial Institute, Thailand | 5 | 111.25 | 0.93 (0.61-1.41) | 0.74 | Not reported |
| Leong PK, 2014 (42) | *Calloselasma rhodostoma* | Malaysia | I.V. | 1.48 (0.92-2.56) μg/g | Haemato-polyvalent snake antivenom | Queen Saovabha Memorial Institute, Thailand | 5 | 22.47 | 9.09 (5.88-14.29) | 7.27 | Not reported |
| Leong PK, 2014 (42) | *Calloselasma rhodostoma* | Indonesia | I.V. | 1.35 (0.78-2.06) μg/g | Haemato-polyvalent snake antivenom | Queen Saovabha Memorial Institute, Thailand | 5 | 11.2 | 12.50 (7.69-20.00) | 10.00 | Not reported |
| Leong PK, 2014 (42) | *Trimeresurus albolabris* | Not reported | I.V. | 0.50 (0.40-0.63) μg/g | Haemato-polyvalent snake antivenom | Queen Saovabha Memorial Institute, Thailand | 5 | 11.24 | 4.55 (2.94-6.67) | 3.64 | Not reported |
| Leong PK, 2014 (42) | *Trimeresurus purpureomaculatus* | Not reported | I.V. | 1.10 (0.75-1.68) μg/g | Haemato-polyvalent snake antivenom | Queen Saovabha Memorial Institute, Thailand | 5 | 21.55 | 5.26 (2.04-14.28) | 4.21 | Not reported |
| Leong PK, 2014 (42) | *Trimeresurus popeiorum* | Not reported | I.V. | 2.00 (1.61-2.48) μg/g | Haemato-polyvalent snake antivenom | Queen Saovabha Memorial Institute, Thailand | 5 | 21.81 | 8.33 (5.56-12.50) | 6.66 | Not reported |
| Leong PK, 2014 (42) | *Tropidolaemus wagleri* | Not reported | I.V. | 1.50 (1.37-1.64) μg/g | Haemato-polyvalent snake antivenom | Queen Saovabha Memorial Institute, Thailand | 2.5 | Not effective | Not effective | Not effective | Not reported |
| Leong PK, 2014 (42) | *Daboia siamensis* | Thailand | I.V. | 0.13 (0.10-0.15) μg/g | Haemato-polyvalent snake antivenom | Queen Saovabha Memorial Institute, Thailand | 5 | 11.24 | 1.27 (0.84-1.92) | 1.02 | Not reported |
| Leong PK, 2014 (42) | *Daboia siamensis* | Myanmar | I.V. | 0.34 (0.08-0.81) μg/g | Haemato-polyvalent snake antivenom | Queen Saovabha Memorial Institute, Thailand | 5 | 35.36 | 5.00 (2.38-11.00) | 4.00 | Not reported |
| Leong PK, 2014 (42) | *Calloselasma rhodostoma* | Malaysia | I.V. | 1.48 (0.92-2.56) μg/g | Malayan pit viper antivenin | Queen Saovabha Memorial Institute, Thailand | 5 | Not reported | 4.00 (1.87-8.33) | Not reported | Not reported |
| Danpaiboon W, 2014 (43) | *Ophiophagus hannah* | Thailand | I.P. | 1.10 μg/g | Cobra antivenin | Queen Saovabha Memorial Institute, Thailand | 1.5 | 19.60 mg/kg | Not reported | Not reported | Not reported |
| Pakmanee N, 2013 (44) | *Daboia siamensis* | Thailand | I.V. | 3.71 μg/mouse | Russell's viper antivenin | Queen Saovabha Memorial Institute, Thailand | 2 | Not reported | 12.40 (12.00-12.70) | 0.60 | Not reported |
| Leong PK, 2012 (45) | *Naja sputatrix* | Not reported | I.V. | 0.90 (0.59-1.36) μg/g | Snake Venom Antiserum I.P. (Asia) | VINS Bioproducts Ltd., India | 2.5 | 156.60 (128-191.6) | 0.33 (0.27-0.40) | 0.20 | Not reported |
| Leong PK, 2012 (45) | *Naja siamensis* | Not reported | I.V. | 0.28 (0.18-0.42) μg/g | Snake Venom Antiserum I.P. (Asia) | VINS Bioproducts Ltd., India | 5 | 50 (40.30-62.00) | 0.65 (0.53-0.80) | 0.52 | Not reported |
| Leong PK, 2012 (45) | *Naja kaouthia* | Thailand | I.V. | 0.23 (0.15-0.34) μg/g | Snake Venom Antiserum I.P. (Asia) | VINS Bioproducts Ltd., India | 5 | 75 (68.50-82.10) | 0.35 (0.32-0.39) | 0.28 | Not reported |
| Leong PK, 2012 (45) | *Naja kaouthia* | Malaysia | I.V. | 0.89 (0.59-1.35) μg/g | Snake Venom Antiserum I.P. (Asia) | VINS Bioproducts Ltd., India | 2.5 | 70.70 (64.10-92.30) | 0.36 (0.32-0.39) | 0.22 | Not reported |
| Leong PK, 2012 (45) | *Naja sumatrana* | Malaysia | I.V. | 0.50 (0.40-0.62) μg/g | Snake Venom Antiserum I.P. (Asia) | VINS Bioproducts Ltd., India | 5 | 39.10 (32.00-47.90) | 1.47 (1.20-1.79) | 1.18 | Not reported |
| Leong PK, 2012 (45) | *Naja philippinensis* | Not reported | I.V. | 0.18 (0.12-0.27) μg/g | Snake Venom Antiserum I.P. (Asia) | VINS Bioproducts Ltd., India | 2.5 | 156.60 (128.00-191.60) | 0.07 (0.05-0.08) | 0.04 | Not reported |
| Leong PK, 2012 (45) | *Ophiophagus hannah* | Not reported | I.V. | 1.00 (0.81-1.24) μg/g | Snake Venom Antiserum I.P. (Asia) | VINS Bioproducts Ltd., India | 2.5 | 156.60 (128.00-191.60) | 0.37 (0.30-0.45) | 0.22 | Not reported |
| Leong PK, 2012 (45) | *Bungarus fasciatus* | Not reported | I.V. | 1.67 (1.10-2.53) μg/g | Snake Venom Antiserum I.P. (Asia) | VINS Bioproducts Ltd., India | Not report | Not effective | Not effective | Not effective | Not reported |
| Leong PK, 2012 (45) | *Bungarus candidus* | Not reported | I.V. | 0.11 (0.07-0.17) μg/g | Snake Venom Antiserum I.P. (Asia) | VINS Bioproducts Ltd., India | 5 | 44.90 (29.60-68.20) | 0.28 (0.19-0.43) | 0.22 | Not reported |
| Leong PK, 2012 (45) | *Naja naja* | India | I.V. | 1.80 (1.18-2.73) μg/g | Snake Venom Antiserum I.P. (Asia) | VINS Bioproducts Ltd., India | 2.5 | 55.60 (36.60-84.50) | 2.86 (1.92-4.35) | 1.71 | Not reported |
| Leong PK, 2012 (45) | *Naja naja* | India | I.V. | 1.08 (0.71-1.64) μg/g | Snake Venom Antiserum I.P. (Asia) | VINS Bioproducts Ltd., India | 2.5 | 37.50 (34.30-41.00) | 1.85 (1.22-2.86) | 1.11 | Not reported |
| Leong PK, 2012 (45) | *Naja naja* | Sri Lanka | I.V. | 1.13 (0.54-2.38) μg/g | Snake Venom Antiserum I.P. (Asia) | VINS Bioproducts Ltd., India | 2.5 | 22.50 (14.80-34.10) | 1.67 (1.52-1.82) | 1.00 | Not reported |
| Leong PK, 2012 (45) | *Naja naja* | Sri Lanka | I.V. | 1.08 (0.71-1.64) μg/g | Snake Venom Antiserum I.P. (Asia) | VINS Bioproducts Ltd., India | 5 | 150 (137.10-164.20) | 0.83 (0.75-0.91) | 0.66 | Not reported |
| Leong PK, 2012 (45) | *Naja sputatrix* | Not reported | I.V. | 0.90 (0.59-1.36) μg/g | Polyvalent Snake Antivenom (Asia) | Bharat Serums & Vaccines, India | Not report | Not effective | Not effective | Not effective | Not reported |
| Leong PK, 2012 (45) | *Naja siamensis* | Not reported | I.V. | 0.28 (0.18-0.42) μg/g | Polyvalent Snake Antivenom (Asia) | Bharat Serums & Vaccines, India | 2.5 | 70.70 (54.10-92.30) | 0.23 (0.17-0.30) | 0.14 | Not reported |
| Leong PK, 2012 (45) | *Naja kaouthia* | Thailand | I.V. | 0.23 (0.15-0.34) μg/g | Polyvalent Snake Antivenom (Asia) | Bharat Serums & Vaccines, India | 5 | 55.60 (36.60-84.50) | 0.46 (0.30-0.70) | 0.37 | Not reported |
| Leong PK, 2012 (45) | *Naja kaouthia* | Malaysia | I.V. | 0.89 (0.59-1.35) μg/g | Polyvalent Snake Antivenom (Asia) | Bharat Serums & Vaccines, India | Not report | Not effective | Not effective | Not effective | Not reported |
| Leong PK, 2012 (45) | *Naja sumatrana* | Malaysia | I.V. | 0.50 (0.40-0.62) μg/g | Polyvalent Snake Antivenom (Asia) | Bharat Serums & Vaccines, India | 5 | 150 (137.10-164.20) | 0.38 (0.36-0.42) | 0.30 | Not reported |
| Leong PK, 2012 (45) | *Naja philippinensis* | Not reported | I.V. | 0.18 (0.12-0.27) μg/g | Polyvalent Snake Antivenom (Asia) | Bharat Serums & Vaccines, India | Not report | Not effective | Not effective | Not effective | Not reported |
| Leong PK, 2012 (45) | *Ophiophagus hannah* | Not reported | I.V. | 1.00 (0.81-1.24) μg/g | Polyvalent Snake Antivenom (Asia) | Bharat Serums & Vaccines, India | Not report | Not effective | Not effective | Not effective | Not reported |
| Leong PK, 2012 (45) | *Bungarus fasciatus* | Not reported | I.V. | 1.67 (1.10-2.53) μg/g | Polyvalent Snake Antivenom (Asia) | Bharat Serums & Vaccines, India | Not report | Not effective | Not effective | Not effective | Not reported |
| Leong PK, 2012 (45) | *Bungarus candidus* | Not reported | I.V. | 0.11 (0.07-0.17) μg/g | Polyvalent Snake Antivenom (Asia) | Bharat Serums & Vaccines, India | 2.5 | 26.70 (12.70-56.40) | 0.24 (0.11-0.50) | 0.14 | Not reported |
| Leong PK, 2012 (45) | *Naja naja* | India | I.V. | 1.80 (1.18-2.73) μg/g | Polyvalent Snake Antivenom (Asia) | Bharat Serums & Vaccines, India | Not report | Not effective | Not effective | Not effective | Not reported |
| Leong PK, 2012 (45) | *Naja naja* | India | I.V. | 1.08 (0.71-1.64) μg/g | Polyvalent Snake Antivenom (Asia) | Bharat Serums & Vaccines, India | Not report | Not effective | Not effective | Not effective | Not reported |
| Leong PK, 2012 (45) | *Naja naja* | Sri Lanka | I.V. | 1.13 (0.54-2.38) μg/g | Polyvalent Snake Antivenom (Asia) | Bharat Serums & Vaccines, India | Not report | Not effective | Not effective | Not effective | Not reported |
| Leong PK, 2012 (45) | *Naja naja* | Sri Lanka | I.V. | 1.08 (0.71-1.64) μg/g | Polyvalent Snake Antivenom (Asia) | Bharat Serums & Vaccines, India | 2.5 | 200 | 0.31 | 0.19 | Not reported |
| Leong PK, 2012 (46) | *Naja sputatrix* | Not reported | I.V. | 0.90 (0.59-1.36) μg/g | Neuro-polyvalent snake antivenom | Queen Saovabha Memorial Institute, Thailand | 5 | 111.25 | 0.93 (0.61-1.41) | 0.74 | Not reported |
| Leong PK, 2012 (46) | *Naja siamensis* | Not reported | I.V. | 0.28 (0.18-0.42) μg/g | Neuro-polyvalent snake antivenom | Queen Saovabha Memorial Institute, Thailand | 5 | 22.47 | 1.43 (0.94-2.18) | 1.15 | Not reported |
| Leong PK, 2012 (46) | *Naja sumatrana* | Malaysia | I.V. | 0.50 (0.40-0.62) μg/g | Neuro-polyvalent snake antivenom | Queen Saovabha Memorial Institute, Thailand | 5 | 25 | 2.30 (1.86-2.85) | 1.84 | Not reported |
| Leong PK, 2012 (46) | *Naja kaouthia* | Thailand | I.V. | 0.23 (0.15-0.34) μg/g | Neuro-polyvalent snake antivenom | Queen Saovabha Memorial Institute, Thailand | 5 | 22.47 | 1.18 (0.78-1.79) | 0.94 | Not reported |
| Leong PK, 2012 (46) | *Naja kaouthia* | Malaysia | I.V. | 0.89 (0.59-1.35) μg/g | Neuro-polyvalent snake antivenom | Queen Saovabha Memorial Institute, Thailand | 5 | 150 | 0.68 (0.62-0.75) | 0.55 | Not reported |
| Leong PK, 2012 (46) | *Naja philippinensis* | Not reported | I.V. | 0.18 (0.12-0.27) μg/g | Neuro-polyvalent snake antivenom | Queen Saovabha Memorial Institute, Thailand | 5 | 156.57 | 0.13 (0.11-0.16) | 0.10 | Not reported |
| Leong PK, 2012 (46) | *Naja atra* | Not reported | I.V. | 0.56 (0.37-0.84) μg/g | Neuro-polyvalent snake antivenom | Queen Saovabha Memorial Institute, Thailand | 2.5 | 56 | 0.86 (0.79-0.94) | 0.52 | Not reported |
| Leong PK, 2012 (46) | *Naja oxiana* | Not reported | I.V. | 1.11 (0.73-1.69) μg/g | Neuro-polyvalent snake antivenom | Queen Saovabha Memorial Institute, Thailand | 2.5 | 37.5 | 1.70 (1.56-1.85) | 1.01 | Not reported |
| Leong PK, 2012 (46) | *Naja naja* | India | I.V. | 1.80 (1.18-2.73) μg/g | Neuro-polyvalent snake antivenom | Queen Saovabha Memorial Institute, Thailand | 2.5 | 200 | 0.52 | 0.31 | Not reported |
| Leong PK, 2012 (46) | *Naja naja* | India | I.V. | 1.08 (0.71-1.64) μg/g | Neuro-polyvalent snake antivenom | Queen Saovabha Memorial Institute, Thailand | 2.5 | 156.57 | 0.40 (0.32-0.49) | 0.24 | Not reported |
| Leong PK, 2012 (46) | *Naja naja* | Sri Lanka | I.V. | 1.13 (0.54-2.38) μg/g | Neuro-polyvalent snake antivenom | Queen Saovabha Memorial Institute, Thailand | 2.5 | 100 | 0.65 (0.52-0.84) | 0.39 | Not reported |
| Leong PK, 2012 (46) | *Naja naja* | Sri Lanka | I.V. | 1.08 (0.71-1.64) μg/g | Neuro-polyvalent snake antivenom | Queen Saovabha Memorial Institute, Thailand | 5 | 89.88 | 1.39 (0.91-2.08) | 1.11 | Not reported |
| Leong PK, 2012 (46) | *Naja haje* | Not reported | I.V. | 0.09 (0.05-1.40) μg/g | Neuro-polyvalent snake antivenom | Queen Saovabha Memorial Institute, Thailand | 5 | 78.29 | 0.13 (0.11-0.16) | 0.10 | Not reported |
| Leong PK, 2012 (46) | *Naja melanoleuca* | Not reported | I.V. | 0.33 (0.22-0.51) μg/g | Neuro-polyvalent snake antivenom | Queen Saovabha Memorial Institute, Thailand | 5 | 55.63 | 0.68 (0.44-1.03) | 0.54 | Not reported |
| Leong PK, 2012 (46) | *Naja nigricollis* | Not reported | I.V. | 0.75 (0.69-0.82) μg/g | Neuro-polyvalent snake antivenom | Queen Saovabha Memorial Institute, Thailand | 2.5 | 55.63 | 0.78 (0.49-1.18) | 0.47 | Not reported |
| Leong PK, 2012 (46) | *Naja nubiae* | Not reported | I.V. | 0.28 (0.22-0.37) μg/g | Neuro-polyvalent snake antivenom | Queen Saovabha Memorial Institute, Thailand | 5 | 78.29 | 0.41 (0.34-0.50) | 0.33 | Not reported |
| Leong PK, 2012 (46) | *Naja katiensis* | Not reported | I.V. | 1.20 (0.97-1.45) μg/g | Neuro-polyvalent snake antivenom | Queen Saovabha Memorial Institute, Thailand | 2.5 | Not effective | Not effective | Not effective | Not reported |
| Leong PK, 2012 (46) | *Ophiophagus hannah* | Malaysia | I.V. | 1.00 (0.81-1.24) μg/g | Neuro-polyvalent snake antivenom | Queen Saovabha Memorial Institute, Thailand | 5 | 11.24 | 10.23 (6.74-15.54) | 8.19 | Not reported |
| Leong PK, 2012 (46) | *Bungarus fasciatus* | Malaysia | I.V. | 1.67 (1.10-2.53) μg/g | Neuro-polyvalent snake antivenom | Queen Saovabha Memorial Institute, Thailand | 5 | 111.25 | 1.73 (1.14-2.62) | 1.38 | Not reported |
| Leong PK, 2012 (46) | *Bungarus candidus* | Malaysia | I.V. | 0.11 (0.07-0.17) μg/g | Neuro-polyvalent snake antivenom | Queen Saovabha Memorial Institute, Thailand | 5 | 13.91 | 0.91 (0.60-1.38) | 0.73 | Not reported |
| Leong PK, 2012 (46) | *Bungarus flaviceps* | Malaysia | I.V. | 0.18 (0.09-0.21) μg/g | Neuro-polyvalent snake antivenom | Queen Saovabha Memorial Institute, Thailand | 5 | 11.24 | 1.84 (1.21-2.80) | 1.47 | Not reported |
| Leong PK, 2012 (46) | *Bungarus multicinctus* | Not reported | I.V. | 0.11 (0.05-0.22) μg/g | Neuro-polyvalent snake antivenom | Queen Saovabha Memorial Institute, Thailand | 5 | 37.5 | 0.34 (0.31-0.37) | 0.27 | Not reported |
| Leong PK, 2012 (46) | *Bungarus caeruleus* | Not reported | I.V. | 0.17 (0.11-0.25) μg/g | Neuro-polyvalent snake antivenom | Queen Saovabha Memorial Institute, Thailand | 5 | 78.29 | 0.12 (0.10-0.15) | 0.07 | Not reported |
| Leong PK, 2012 (46) | *Naja sputatrix* | Not reported | I.V. | 0.90 (0.59-1.36) μg/g | Cobra antivenin | Queen Saovabha Memorial Institute, Thailand | 5 | 111.25 | 0.93 (0.61-1.41) | 0.74 | Not reported |
| Leong PK, 2012 (46) | *Naja siamensis* | Not reported | I.V. | 0.28 (0.18-0.42) μg/g | Cobra antivenin | Queen Saovabha Memorial Institute, Thailand | 5 | 22.47 | 1.43 (0.94-2.18) | 1.15 | Not reported |
| Leong PK, 2012 (46) | *Naja sumatrana* | Malaysia | I.V. | 0.50 (0.40-0.62) μg/g | Cobra antivenin | Queen Saovabha Memorial Institute, Thailand | 5 | 50 | 1.15 (0.93-1.43) | 0.92 | Not reported |
| Leong PK, 2012 (46) | *Naja kaouthia* | Thailand | I.V. | 0.23 (0.15-0.34) μg/g | Cobra antivenin | Queen Saovabha Memorial Institute, Thailand | 5 | 22.47 | 1.18 (0.78-1.79) | 0.94 | Not reported |
| Leong PK, 2012 (46) | *Naja kaouthia* | Malaysia | I.V. | 0.89 (0.59-1.35) μg/g | Cobra antivenin | Queen Saovabha Memorial Institute, Thailand | 5 | 150 | 0.68 (0.62-0.75) | 0.55 | Not reported |
| Leong PK, 2012 (46) | *Ophiophagus hannah* | Malaysia | I.V. | 1.00 (0.81-1.24) μg/g | Cobra antivenin | Queen Saovabha Memorial Institute, Thailand | 5 | 44.94 | 3.07 (2.80-3.35) | 2.46 | Not reported |
| Tan CH, 2011 (47) | *Calloselasma rhodostoma* | Malaysia | I.V. | 1.48 (0.78-2.06) μg/g | Malayan pit viper antivenin | Queen Saovabha Memorial Institute, Thailand | 5 | 41.53 (20.40-88.40) | Not reported | 3.23 | Not reported |
| Tan CH, 2011 (47) | *Hypnale hypnale* | Sri Lanka | I.V. | 0.90 (0.42-1.84) μg/g | Malayan pit viper antivenin | Queen Saovabha Memorial Institute, Thailand | 5 | 70.71 (33.70-148.40) | Not reported | 0.89 | Not reported |
| Tan CH, 2011 (47) | *Calloselasma rhodostoma* | Malaysia | I.V. | 1.48 (0.78-2.06) μg/g | Haemato-polyvalent snake antivenom | Queen Saovabha Memorial Institute, Thailand | 5 | 22.47 (14.80-34.10) | Not reported | 7.14 | Not reported |
| Tan CH, 2011 (47) | *Hypnale hypnale* | Sri Lanka | I.V. | 0.90 (0.42-1.84) μg/g | Haemato-polyvalent snake antivenom | Queen Saovabha Memorial Institute, Thailand | 5 | 41.53 (20.40-88.40) | Not reported | 1.52 | Not reported |
| Tan CH, 2011 (47) | *Daboia russelii* | Sri Lanka | I.V. | 0.24 (0.19-0.62) μg/g | Haemato-polyvalent snake antivenom | Queen Saovabha Memorial Institute, Thailand | 5 | 7.52 (3.53-15.30) | Not reported | 2.50 | Not reported |
| Tan CH, 2011 (47) | *Echis carinatus sochureki* | Pakistan | I.V. | 2.08 (1.02-4.42) μg/g | Haemato-polyvalent snake antivenom | Queen Saovabha Memorial Institute, Thailand | 5 | > 200 | Not reported | Not effective | Not reported |
| Chanhome L, 2002 (48) | *Trimeresurus albolabris* | Thailand | I.V. | 10 μg/mouse | Green pit viper antivenin | Queen Saovabha Memorial Institute, Thailand | 4 | 14 | Not reported | 1.40 | Not reported |
| Chanhome L, 2002 (48) | *Trimeresurus macrops* | Thailand | I.V. | 140 μg/mouse | Green pit viper antivenin | Queen Saovabha Memorial Institute, Thailand | 4 | 112 | Not reported | 2.50 | Not reported |
| Chanhome L, 2002 (48) | *Trimeresurus popeiorum* | Thailand | I.V. | 35 μg/mouse | Green pit viper antivenin | Queen Saovabha Memorial Institute, Thailand | 4 | 112 | Not reported | 0.63 | Not reported |
| Chanhome L, 2002 (48) | *Trimeresurus hageni* | Thailand | I.V. | 10 μg/mouse | Green pit viper antivenin | Queen Saovabha Memorial Institute, Thailand | 4 | 56 | Not reported | 0.36 | Not reported |
| Chanhome L, 2002 (48) | *Trimeresurus purpureomaculatus* | Thailand | I.V. | 8 μg/mouse | Green pit viper antivenin | Queen Saovabha Memorial Institute, Thailand | 4 | 56 | Not reported | 0.29 | Not reported |
| Chanhome L, 2002 (48) | *Trimeresurus kanburiensis* | Thailand | I.V. | 60 μg/mouse | Green pit viper antivenin | Queen Saovabha Memorial Institute, Thailand | 4 | 114 | Not reported | 1.06 | Not reported |
| Khow O, 2001 (49) | *Naja kaouthia* | Thailand | I.V. | 6.50 (4.70-8.90) μg/mouse | Cobra antivenin | Queen Saovabha Memorial Institute, Thailand | 4 | 29.60 (24.50-37.70) | Not reported | Not reported | Not reported |
| Khow O, 2001 (49) | *Naja kaouthia* | Thailand | I.V. | 6.50 (4.70-8.90) μg/mouse | Cobra antivenin | Queen Saovabha Memorial Institute, Thailand | 4 | 41.40 (33.60-52.50) | Not reported | Not reported | Not reported |
| Khow O, 2001 (49) | *Naja kaouthia* | Thailand | I.V. | 6.50 (4.70-8.90) μg/mouse | Cobra antivenin | Queen Saovabha Memorial Institute, Thailand | 4 | 45.90 (36.70-59.70) | Not reported | Not reported | Not reported |
| Khow O, 2001 (49) | *Naja kaouthia* | Thailand | I.V. | 6.50 (4.70-8.90) μg/mouse | Cobra antivenin | Queen Saovabha Memorial Institute, Thailand | 4 | 29.60 (24.50-37.70) | Not reported | Not reported | Not reported |
| Khow O, 2001 (49) | *Lapemis hardwickii* | Japan | I.V. | 6.50 (4.70-8.90) μg/mouse | Cobra antivenin | Queen Saovabha Memorial Institute, Thailand | 4 | 91.8 | Not reported | Not reported | Not reported |
| Khow O, 2001 (49) | *Lapemis hardwickii* | Japan | I.V. | 6.50 (4.70-8.90) μg/mouse | Cobra antivenin | Queen Saovabha Memorial Institute, Thailand | 4 | 128.50 (102.40-166.50) | Not reported | Not reported | Not reported |
| Khow O, 2001 (49) | *Lapemis hardwickii* | Japan | I.V. | 6.50 (4.70-8.90) μg/mouse | Cobra antivenin | Queen Saovabha Memorial Institute, Thailand | 4 | 118.3 (94.70-153.40) | Not reported | Not reported | Not reported |
| Khow O, 2001 (49) | *Lapemis hardwickii* | Japan | I.V. | 6.50 (4.70-8.90) μg/mouse | Cobra antivenin | Queen Saovabha Memorial Institute, Thailand | 4 | 91.80 (73.40-120.00) | Not reported | Not reported | Not reported |
| Chanhome L, 1999 (50) | *Bungarus fasciatus* | Thailand | I.V. | 61.70 (43.50-87.50) μg/mouse | Banded krait antivenin | Queen Saovabha Memorial Institute, Thailand | 4 | 432.40 (312.60-598.80) | Not reported | Not reported | Not reported |
| Chanhome L, 1999 (50) | *Bungarus candidus* | Thailand | I.V. | 3.20 (2.50-4.20) μg/mouse | Banded krait antivenin | Queen Saovabha Memorial Institute, Thailand | 4 | 319.70 (251.80-406.00) | Not reported | Not reported | Not reported |
| Chanhome L, 1999 (50) | *Bungarus flaviceps* | Thailand | I.V. | 3.40 (2.60-4.40) μg/mouse | Banded krait antivenin | Queen Saovabha Memorial Institute, Thailand | 4 | 178.80 (138.30-230.00) | Not reported | Not reported | Not reported |
| Khow O, 1997 (51) | *Naja kaouthia* | Thailand | I.V. | 6.61 (5.20-8.39) μg/mouse | Cobra antivenin | Queen Saovabha Memorial Institute, Thailand | 4 | 37.20 (29.00-47.50) | Not reported | Not reported | Not reported |
| Khow O, 1997 (51) | *Naja siamensis* | Thailand | I.V. | 21.40 (14.39-31.80) μg/mouse | Cobra antivenin | Queen Saovabha Memorial Institute, Thailand | 4 | 91.60 (66.20-126.80) | Not reported | Not reported | Not reported |
| Khow O, 1997 (51) | *Naja kaouthia* | Thailand | I.V. | 6.61 (5.20-8.39) μg/mouse | Cobra antivenin | Queen Saovabha Memorial Institute, Thailand | 4 | 28.50 (20.80-39.10) | Not reported | Not reported | Not reported |
| Khow O, 1997 (51) | *Naja siamensis* | Thailand | I.V. | 21.40 (14.39-31.80) μg/mouse | Cobra antivenin | Queen Saovabha Memorial Institute, Thailand | 4 | 91.60 (66.20-126.80) | Not reported | Not reported | Not reported |
| Khow O, 1997 (51) | *Naja kaouthia* | Thailand | I.V. | 6.61 (5.30-8.39) μg/mouse | Cobra antivenin | Queen Saovabha Memorial Institute, Thailand | 4 | 38.40 (29.80-52.50) | Not reported | Not reported | Not reported |
| Khow O, 1997 (51) | *Naja siamensis* | Thailand | I.V. | 21.40 (14.39-31.8) μg/mouse | Cobra antivenin | Queen Saovabha Memorial Institute, Thailand | 4 | 135.90 (114.00-171.00) | Not reported | Not reported | Not reported |
| Sells PG, 1994 (52) | *Naja kaouthia* | Thailand | I.V. | 17.50 μg/mouse | Cobra antivenin | Queen Saovabha Memorial Institute, Thailand | 5 | 6.80 (6.76-6.90) mg | Not reported | Not reported | Not reported |

**References**

1. Tan KY, Shamsuddin NN, Tan CH. Sharp-nosed Pit Viper (Deinagkistrodon acutus) from Taiwan and China: A comparative study on venom toxicity and neutralization by two specific antivenoms across the Strait. Acta Trop. 2022;232:106495.

2. Chanhome L, Khow O, Reamtong O, Vasaruchapong T, Laoungbua P, Tawan T, et al. Biochemical and proteomic analyses of venom from a new pit viper, Protobothrops kelomohy. Journal of Venomous Animals and Toxins Including Tropical Diseases. 2022;28:14.

3. Wong KY, Tan KY, Tan NH, Gnanathasan CA, Tan CH. Elucidating the Venom Diversity in Sri Lankan Spectacled Cobra (Naja naja) through De Novo Venom Gland Transcriptomics, Venom Proteomics and Toxicity Neutralization. Toxins. 2021;13(8):30.

4. Faisal T, Tan KY, Tan NH, Sim SM, Gnanathasan CA, Tan CH. Proteomics, toxicity and antivenom neutralization of Sri Lankan and Indian Russell's viper (Daboia russelii) venoms. Journal of Venomous Animals and Toxins Including Tropical Diseases. 2021;27:15.

5. Attarde S, Khochare S, Iyer A, Dam P, Martin G, Sunagar K. Venomics of the Enigmatic Andaman Cobra (Naja sagittifera) and the Preclinical Failure of Indian Antivenoms in Andaman and Nicobar Islands. Frontiers in Pharmacology. 2021;12:16.

6. Tan CH, Palasuberniam P, Blanco FB, Tan KY. Immunoreactivity and neutralization capacity of Philippine cobra antivenom against Naja philippinensis and Naja samarensis venoms. Transactions of the Royal Society of Tropical Medicine and Hygiene. 2021;115(1):78-84.

7. Oh AMF, Tan KY, Tan NH, Tan CH. Proteomics and neutralization of Bungarus multicinctus (Many-banded Krait) venom: Intra-specific comparisons between specimens from China and Taiwan. Comparative Biochemistry and Physiology Part - C: Toxicology and Pharmacology. 2021;247.

8. Laxme RRS, Khochare S, Attarde S, Suranse V, Iyer A, Casewell NR, et al. Biogeographic venom variation in Russell's viper (Daboia russelii) and the preclinical inefficacy of antivenom therapy in snakebite hotspots. Plos Neglected Tropical Diseases. 2021;15(3).

9. Laxme RRS, Attarde S, Khochare S, Suranse V, Martin G, Casewell NR, et al. Biogeographical venom variation in the Indian spectacled cobra (Naja naja) underscores the pressing need for pan-India efficacious snakebite therapy. Plos Neglected Tropical Diseases. 2021;15(2).

10. Yee KT, Maw LZ, Kyaw AM, Khow O, Oo AW, Oo TKK, et al. Evaluation of the cross-neutralization capacity of Thai green pit viper antivenom against venom of Myanmar green pit viper. Toxicon. 2020;177:41-5.

11. Tan KY, Ng TS, Bourges A, Ismail AK, Maharani T, Khomvilai S, et al. Geographical variations in king cobra (Ophiophagus hannah) venom from Thailand, Malaysia, Indonesia and China: On venom lethality, antivenom immunoreactivity and in vivo neutralization. Acta Tropica. 2020;203.

12. Lin B, Zhang JR, Lu HJ, Zhao L, Chen J, Zhang HF, et al. Immunoreactivity and neutralization study of chinese bungarus multicinctus antivenin and lab-prepared anti-bungarotoxin antisera towards purified bungarotoxins and snake venoms. PLoS Neglected Tropical Diseases. 2020;14(11):1-19.

13. Liew JL, Tan NH, Tan CH. Proteomics and preclinical antivenom neutralization of the mangrove pit viper (Trimeresurus purpureomaculatus, Malaysia) and white-lipped pit viper (Trimeresurus albolabris, Thailand) venoms. Acta Tropica. 2020;209.

14. Lee LP, Tan KY, Tan CH. Toxicity and cross-neutralization of snake venoms from two lesser-known arboreal pit vipers in Southeast Asia: Trimeresurus wiroti and Trimeresurus puniceus. Toxicon. 2020;185:91-6.

15. Hia YL, Tan KY, Tan CH. Comparative venom proteomics of banded krait (Bungarus fasciatus) from five geographical locales: Correlation of venom lethality, immunoreactivity and antivenom neutralization. Acta Tropica. 2020;207.

16. Choraria A, Somasundaram R, Gautam M, Ramanathan M, Paray BA, Al-Sadoon MK, et al. Experimental antivenoms from chickens and rabbits and their comparison with commercially available equine antivenom against the venoms of Daboia russelii and Echis carinatus snakes. Toxin Reviews. 2020.

17. Tan CH, Tan KY, Ng TS, Quah ESH, Ismail AK, Khomvilai S, et al. Venomics of trimeresurus (Popeia) nebularis, the cameron highlands pit viper from Malaysia: Insights into venom proteome, toxicity and neutralization of antivenom. Toxins. 2019;11(2).

18. Pla D, Sanz L, Quesada-Bernat S, Villalta M, Baal J, Chowdhury MAW, et al. Phylovenomics of Daboia russelii across the Indian subcontinent. Bioactivities and comparative in vivo neutralization and in vitro third-generation antivenomics of antivenoms against venoms from India, Bangladesh and Sri Lanka. Journal of Proteomics. 2019;207.

19. Oh AMF, Tan CH, Tan KY, Quraishi NH, Tan NH. Venom proteome of Bungarus sindanus (Sind krait) from Pakistan and in vivo cross-neutralization of toxicity using an Indian polyvalent antivenom. Journal of Proteomics. 2019;193:243-54.

20. Lingam TMC, Tan KY, Tan CH. Thai Russell's viper monospecific antivenom is immunoreactive and effective in neutralizing the venom of Daboia siamensis from Java, Indonesia. Toxicon. 2019;168:95-7.

21. Laxme RRS, Khochare S, de Souza HF, Ahuja B, Suranse V, Martin G, et al. Beyond the 'big four': Venom profiling of the medically important yet neglected Indian snakes reveals disturbing antivenom deficiencies. Plos Neglected Tropical Diseases. 2019;13(12).

22. Deka A, Abu Reza M, Hoque KMF, Deka K, Saha S, Doley R. Comparative analysis of Naja kaouthia venom from North-East India and Bangladesh and its cross reactivity with Indian polyvalent antivenoms. Toxicon. 2019;164:31-43.

23. Chaisakul J, Alsolaiss J, Charoenpitakchai M, Wiwatwarayos K, Sookprasert N, Harrison RA, et al. Evaluation of the geographical utility of Eastern Russell’s viper (Daboia siamensis) antivenom from Thailand and an assessment of its protective effects against venom-induced nephrotoxicity. PLoS Neglected Tropical Diseases. 2019;13(10).

24. Tan CH, Tan KY, Ng TS, Sim SM, Tan NH. Venom Proteome of Spine-Bellied Sea Snake (Hydrophis curtus) from Penang, Malaysia: Toxicity Correlation, Immunoprofiling and Cross-Neutralization by Sea Snake Antivenom. Toxins (Basel). 2018;11(1).

25. Tan KY, Tan NH, Tan CH. Venom proteomics and antivenom neutralization for the Chinese eastern Russell's viper, Daboia siamensis from Guangxi and Taiwan. Scientific reports. 2018;8(1):8545.

26. Sanz L, Quesada-Bernat S, Chen PY, Lee CD, Chiang JR, Calvete JJ. Translational Venomics: Third-Generation Antivenomics of Anti-Siamese Russell's Viper, Daboia siamensis, Antivenom Manufactured in Taiwan CDC's Vaccine Center. Trop Med Infect Dis. 2018;3(2).

27. Liu BS, Wu WG, Lin MH, Li CH, Jiang BR, Wu SC, et al. Identification of immunoreactive peptides of toxins to simultaneously assess the neutralization potency of antivenoms against neurotoxicity and cytotoxicity of Naja atra venom. Toxins. 2018;10(1).

28. Faisal T, Tan KY, Sim SM, Quraishi N, Tan NH, Tan CH. Proteomics, functional characterization and antivenom neutralization of the venom of Pakistani Russell's viper (Daboia russelii) from the wild. Journal of Proteomics. 2018;183:1-13.

29. Tan CH, Liew JL, Tan NH, Ismail AK, Maharani T, Khomvilai S, et al. Cross reactivity and lethality neutralization of venoms of Indonesian Trimeresurus complex species by Thai Green Pit Viper Antivenom. Toxicon. 2017;140:32-7.

30. Tan CH, Wong KY, Tan KY, Tan NH. Venom proteome of the yellow-lipped sea krait, Laticauda colubrina from Bali: Insights into subvenomic diversity, venom antigenicity and cross-neutralization by antivenom. J Proteomics. 2017;166:48-58.

31. Oh AMF, Tan CH, Ariaranee GC, Quraishi N, Tan NH. Venomics of Bungarus caeruleus (Indian krait): Comparable venom profiles, variable immunoreactivities among specimens from Sri Lanka, India and Pakistan. Journal of Proteomics. 2017;164:1-18.

32. Wong KY, Tan CH, Tan NH. Venom and purified toxins of the spectacled cobra (Naja naja) from Pakistan: Insights into toxicity and antivenom neutralization. American Journal of Tropical Medicine and Hygiene. 2016;94(6):1392-9.

33. Villalta M, Sánchez A, Herrera M, Vargas M, Segura Á, Cerdas M, et al. Development of a new polyspecific antivenom for snakebite envenoming in Sri Lanka: Analysis of its preclinical efficacy as compared to a currently available antivenom. Toxicon. 2016;122:152-9.

34. Tan KY, Tan CH, Fung SY, Tan NH. Neutralization of the principal toxins from the venoms of thai naja kaouthia and malaysian hydrophis schistosus: Insights into toxin-specific neutralization by two different antivenoms. Toxins. 2016;8(4).

35. Tan CH, Liew JL, Tan KY, Tan NH. Assessing SABU (Serum Anti Bisa Ular), the sole Indonesian antivenom: A proteomic analysis and neutralization efficacy study. Scientific reports. 2016;6:37299.

36. Tan CH, Tan KY, Tan NH. Revisiting Notechis scutatus venom: on shotgun proteomics and neutralization by the "bivalent" Sea Snake Antivenom. J Proteomics. 2016;144:33-8.

37. Maduwage K, Silva A, O'Leary MA, Hodgson WC, Isbister GK. Efficacy of Indian polyvalent snake antivenoms against Sri Lankan snake venoms: lethality studies or clinically focussed in vitro studies. Scientific reports. 2016;6:26778.

38. Yap MK, Tan NH, Sim SM, Fung SY, Tan CH. The Effect of a Polyvalent Antivenom on the Serum Venom Antigen Levels of Naja sputatrix (Javan Spitting Cobra) Venom in Experimentally Envenomed Rabbits. Basic Clin Pharmacol Toxicol. 2015;117(4):274-9.

39. Tan KY, Tan CH, Fung SY, Tan NH. Venomics, lethality and neutralization of Naja kaouthia (monocled cobra) venoms from three different geographical regions of Southeast Asia. Journal of Proteomics. 2015;120:105-25.

40. Tan CH, Tan NH, Tan KY, Kwong KO. Antivenom cross-neutralization of the venoms of Hydrophis schistosus and Hydrophis curtus, two common sea snakes in Malaysian waters. Toxins. 2015;7(2):572-81.

41. Leong PK, Fung SY, Tan CH, Sim SM, Tan NH. Immunological cross-reactivity and neutralization of the principal toxins of Naja sumatrana and related cobra venoms by a Thai polyvalent antivenom (Neuro Polyvalent Snake Antivenom). Acta Tropica. 2015;149:86-93.

42. Leong PK, Tan CH, Sim SM, Fung SY, Sumana K, Sitprija V, et al. Cross neutralization of common Southeast Asian viperid venoms by a Thai polyvalent snake antivenom (Hemato Polyvalent Snake Antivenom). Acta Tropica. 2014;132(1):7-14.

43. Danpaiboon W, Reamtong O, Sookrung N, Seesuay W, Sakolvaree Y, Thanongsaksrikul J, et al. Ophiophagus hannah venom: Proteome, components bound by Naja kaouthia antivenin and neutralization by n. kaouthia neurotoxin-specific human ScFv. Toxins. 2014;6(5):1526-58.

44. Pakmanee N, Noiphrom J, Kay A, Pornmuttakun D, Sakolparp L, Hemmala W, et al. Comparative abilities of IgG and F(ab ')(2) monovalent antivenoms to neutralize lethality, phospholipase A(2), and coagulant activities induced by Daboia siamensis venom and their anticomplementary activity. Scienceasia. 2013;39(2):160-6.

45. Leong PK, Tan NH, Fung SY, Sim SM. Cross neutralisation of Southeast Asian cobra and krait venoms by Indian polyvalent antivenoms. Transactions of the Royal Society of Tropical Medicine and Hygiene. 2012;106(12):731-7.

46. Leong PK, Sim SM, Fung SY, Sumana K, Sitprija V, Tan NH. Cross neutralization of afro-asian cobra and asian krait venoms by a thai polyvalent snake antivenom (neuro polyvalent snake antivenom). PLoS Neglected Tropical Diseases. 2012;6(6).

47. Tan CH, Leong PK, Fung SY, Sim SM, Ponnudurai G, Ariaratnam C, et al. Cross neutralization of Hypnale hypnale (hump-nosed pit viper) venom by polyvalent and monovalent Malayan pit viper antivenoms in vitro and in a rodent model. Acta Tropica. 2011;117(2):119-24.

48. Chanhome L, Khow O, Omori-Satoh T, Sitprija V. Capacity of Thai green pit viper antivenom to neutralize the venoms of Thai Trimeresurus snakes and comparison of biological activities of these venoms. Journal of natural toxins. 2002;11(3):251-9.

49. Khow O, Chanhome L, Omori-Satoh T, Sitprija V. Effectiveness of Thai cobra (Naja kaouthia) antivenom against sea snake (Lapemis hardwickii) venom: verification by affinity purified F(AB')2 fragments. J Nat Toxins. 2001;10(3):249-53.

50. Chanhome L, Wongtongkam N, Khow O, Pakmanee N, Omori-Satoh T, Sitprija V. Genus specific neutralization of Bungarus snake venoms by Thai Red Cross banded krait antivenom. Journal of Natural Toxins. 1999;8(1):135-40.

51. Khow O, Pakmanee N, Chanhome L, Sriprapat S, Omori-Satoh T, Sitprija V. Cross-neutralization of Thai cobra (Naja kaouthia) and spitting cobra (Naja siamensis) venoms by Thai cobra antivenom. Toxicon. 1997;35(11):1649-51.

52. Sells PG, Jones RG, Laing GD, Smith DC, Theakston RD. Experimental evaluation of ovine antisera to Thai cobra (Naja kaouthia) venom and its alpha-neurotoxin. Toxicon. 1994;32(12):1657-65.
