# Supplementary material for: Antivenom preclinical efficacy testing against Asian snakes and their availability in Asia: A systematic review
Source: PLoS One. 2023 Jul 19;18(7):e0288723. doi: 10.1371/journal.pone.0288723 (PMC10355433; doi:10.1371/journal.pone.0288723)
Supplement: S6 Table — (DOCX) [file pone.0288723.s006.docx]

# **S6 Table.** **Assessment of reporting guideline for in vivo neutralization of lethality of antivenom assessment.**

| **Author, year** | **Experiment set up** | | | | | **Animals** | | | | | **Procedure** | | | | | | | | **Reported results** | | | | |
| --- | --- | --- | --- | --- | --- | --- | --- | --- | --- | --- | --- | --- | --- | --- | --- | --- | --- | --- | --- | --- | --- | --- | --- |
|  | Antivenom batch | Total protein conc.  of AV | Geographic origin  of venoms | Ethical statement | Conflicts of interest  statement | Source of animals | Mouse strain | Weight (g) | Mouse sex | Husbandry | Control groups  stated | LD50 used for ED50 | Number per group | Total number of animals  used groups | Pre-incubation | Route | Injection Volume  (ml) | Experiment length  (hr) | Group outcome  reporting | Adverse events | Description of  statistical analysis | ED50 calculation  method | ED50 reporting |
| Tan KY, 2022 (1) | Yes | No | Yes | Yes | Yes | Yes | ICR | 20-25 | No | No | Yes | 2 | 4 | No | Yes | I.V. | No | 24 | No | No | Yes | Probit analysis | mcl |
| Chanhome O, 2022 (2) | Yes | No | Yes | Yes | Yes | Yes | ICR | 18-20 | Yes (Male) | No | Yes | 2, 2.5, 3 | 5 | No | Yes | I.V. | 0.2 | 24 | No | No | Yes | Reed and Muench | mcl |
| Wong KY, 2021 (3) | Yes | No | Yes | No | Yes | No | ICR | 20-25 | No | No | Yes | 2.5 | 4 | No | Yes | I.V. | 0.15, 0.35, 0.75, 1.00 | 24 | No | No | No | Probit analysis | mcl |
| Faisal T, 2021 (4) | Yes | Yes | Yes | Yes | Yes | Yes | ICR | 20-25 | No | No | Yes | 5 | No | No | Yes | I.V. | No | 24 | No | No | Yes | Probit analysis | mcl |
| Attarde S, 2021 (5) | Yes | Yes | Yes | Yes | Yes | No | CD1 | No | Yes (Male) | No | Yes | 3, 5 | 5 | No | Yes | I.V. | 0.2 | 24 | No | No | Yes | N/A | mcl |
| Tan CH, 2021 (6) | Yes | Yes | Yes | Yes | Yes | Yes | ICR | 20-25 | No | No | Yes | 5 | 4 | No | Yes | I.V. | No | 24 | No | No | Yes | Probit analysis | mcl |
| Oh AMF, 2021 (7) | Yes | Yes | Yes | Yes | Yes | Yes | ICR | 20-25 | No | No | Yes | 5 | 4 | No | Yes | I.V. | 0.25 | 24 | No | No | No | Probit analysis | mg/ml |
| Laxme RRS, 2021 (8) | Yes | Yes | Yes | Yes | Yes | No | CD1 | 18-22 | Yes (Male) | No | Yes | 5 | 5 | No | Yes | I.V. | No | 24 | No | No | Yes | Probit analysis | mcl, mcl/mg |
| Laxme RRS, 2021 (9) | Yes | Yes | Yes | Yes | Yes | No | CD1 | 18-22 | Yes (Male) | No | Yes | 5 | 5 | No | Yes | I.V. | No | 24 | No | No | Yes | Probit analysis | mcl |
| Yee KT, 2020 (10) | Yes | Yes | Yes | Yes | Yes | Yes | ICR | 18-20 | Yes (Male) | No | Yes | 3, 3.5 | 6 | No | Yes | I.V. | 0.2 | 24 | No | No | No | N/A | mcl |
| Tan KY, 2020 (11) | Yes | Yes | Yes | Yes | Yes | Yes | ICR | 20-30 | No | No | Yes | 2.5, 5 | 4 | No | Yes | I.V. | 0.2 | 24 | No | No | Yes | Probit analysis | mcl |
| Lin B, 2020 (12) | Yes | Yes | Yes | Yes | Yes | Yes | Kunming mice | 20 ± 2 | Yes (Male) | No | Yes | 3 | 6 | No | Yes | I.P. | 0.2 | 48 | No | No | No | Spearman-Karber method | mg/kg |
| Liew JL, 2020 (13) | Yes | Yes | Yes | Yes | Yes | Yes | ICR | 20-25 | No | No | Yes | 5 | 4 | No | Yes | I.V. | 0.2 | 48 | No | No | Yes | Probit analysis | mcl |
| Lee LP, 2020 (14) | Yes | Yes | Yes | Yes | Yes | Yes | ICR | 20-25 | No | No | Yes | 2.5 | 4 | No | Yes | I.V. | No | 24 | No | No | No | Probit analysis | mcl |
| Hia YL, 2020 (15) | Yes | Yes | Yes | Yes | Yes | Yes | ICR | 20-25 | No | No | Yes | 2.5 | 4 | No | Yes | I.V. | 0.25 | 24 | No | No | No | Probit analysis | mcl |
| Choraria A, 2020 (16) | Yes | Yes | Yes | Yes | Yes | Yes | Swiss | 18-25 | No | No | Yes | 3 | 4 | No | Yes | I.V. | 0.2 | 48 | No | No | Yes | Probit analysis | mcl/mice |
| Tan CH, 2019 (17) | Yes | Yes | Yes | Yes | Yes | No | ICR | 20-25 | No | No | Yes | 5 | 4 | No | Yes | I.V. | No | 24 | No | No | Yes | Probit analysis | mcl |
| Pla D, 2019 (18) | No | No | Yes | Yes | Yes | No | CD1 | 18-20 | No | No | Yes | 3 | 5 | No | Yes | I.V. | 0.2 | 24 | No | No | Yes | Probit analysis | mg/ml |
| Oh AMF, 2019 (19) | Yes | Yes | Yes | Yes | Yes | Yes | ICR | 20-25 | No | No | Yes | 5 | 4 | No | Yes | I.V. | 0.25 | 48 | No | No | No | Probit analysis | mcl |
| Lingam TMC, 2019 (20) | Yes | Yes | Yes | Yes | No | No | ICR | 20-25 | No | No | No | 5 | 4 | No | Yes | I.V. | 0.2 | 24 | No | No | No | Probit analysis | mcl |
| Laxme RRS, 2019 (21) | Yes | Yes | Yes | Yes | Yes | Yes | CD1 | 18-22 | Yes (Male) | No | Yes | 5 | 5 | No | Yes | I.V. | No | 24 | No | No | Yes | Probit analysis | mcl |
| Deka A, 2019 (22) | Yes | Yes | Yes | Yes | Yes | Yes | Swiss | 25 ± 4 | Yes (Male) | No | Yes | 4 | 6 | No | Yes | I.P. | 0.25 | 24 | No | No | No | Probit analysis | mg/g |
| Chaisakul J, 2019 (23) | Yes | Yes | Yes | Yes | Yes | Yes | ICR | 18-22 | Yes (Male) | No | Yes | 6 | 4-5 | No | Yes | I.V. | 0.2 | 24 | No | No | Yes | Probit analysis | mcl/mice |
| Tan CH, 2018 (24) | No | Yes | Yes | Yes | Yes | No | ICR | 20-25 | No | No | Yes | 5 | 4 | No | Yes | I.V. | No | 24 | No | No | Yes | Probit analysis | mcl |
| Tan KY, 2018 (25) | Yes | Yes | Yes | Yes | Yes | Yes | ICR | 20-30 | No | No | Yes | 5 | 4 | No | Yes | I.V. | No | 48 | No | No | No | Probit analysis | mcl |
| Sanz L, 2018 (26) | Yes | Yes | Yes | Yes | Yes | No | ICR | 11-13 | No | No | Yes | 8 | 10 | No | Yes | I.P. | 0.2 | 48 | No | No | Yes | Probit analysis | mg/g |
| Liu BS, 2018 (27) | Yes | No | Yes | Yes | Yes | Yes | ICR | 19-21 | No | No | Yes | 5 | 6 | No | Yes | I.P. | 0.2 | 48 | No | No | Yes | Spearman–Karber method | mg/g |
| Faisal T, 2018 (28) | Yes | No | Yes | Yes | Yes | Yes | ICR | 20-25 | No | No | Yes | 5 | No | No | Yes | I.V. | No | 24 | No | No | No | Probit analysis | mcl |
| Tan CH, 2017 (29) | Yes | Yes | Yes | Yes | Yes | Yes | ICR | 20-25 | No | No | Yes | 2.5, 5 | 4 | No | Yes | I.V. | 0.2 | 48 | No | No | No | Probit analysis | mcl |
| Tan CH, 2017 (30) | Yes | Yes | Yes | Yes | No | Yes | ICR | 20-25 | No | No | Yes | 5 | 4 | No | Yes | I.V. | 0.25 | 48 | No | No | Yes | Probit analysis | mcl |
| Oh AMF, 2017 (31) | Yes | No | Yes | Yes | Yes | Yes | ICR | 20-25 | No | No | Yes | 5 | 4 | No | Yes | I.V. | 0.1 | 48 | No | No | No | Probit analysis | mcl |
| Wong KY, 2016 (32) | Yes | No | Yes | Yes | Yes | Yes | ICR | 20-25 | No | No | Yes | 5 | 4 | No | Yes | I.V. | 0.2 | 24 | No | No | No | Probit analysis | mcl |
| Villalta M, 2016 (33) | Yes | Yes | Yes | Yes | Yes | No | CD1 | 18-20 | No | No | Yes | 3 | 5 | No | Yes | I.V. | 0.2 | 24 | No | No | Yes | Probit analysis | mg/ml, mg/g |
| Tan KY, 2016 (34) | Yes | Yes | Yes | Yes | Yes | Yes | ICR | 20-25 | No | No | Yes | 5 | 4 | No | Yes | I.V. | 0.2 | 24 | No | No | No | Probit analysis | mcl |
| Tan CH, 2016 (35) | Yes | Yes | Yes | Yes | Yes | Yes | ICR | 20-25 | No | No | Yes | 2.5, 5 | 4 | No | Yes | I.V. | 0.2 | 48 | No | No | No | Probit analysis | mcl |
| Tan CH, 2016 (36) | Yes | No | Yes | Yes | No | Yes | ICR | 20-25 | No | No | Yes | 5 | 4 | No | Yes | I.V. | 0.2 | 48 | No | No | No | Probit analysis | mcl |
| Maduwage K, 2016 (37) | Yes | Yes | Yes | Yes | Yes | No | ICR | 18-20 | No | No | Yes | 5 | 5 | No | Yes | I.V. | 0.25 | 48 | No | No | Yes | Probit analysis | mcg/g |
| Yap MK, 2015 (38) | Yes | No | No | Yes | Yes | Yes | ICR | 18-20 | No | No | Yes | 2.5 | 4 | No | Yes | I.V. | 0.3 | 24 | No | No | Yes | Probit analysis | mcl |
| Tan KY, 2015 (39) | Yes | No | Yes | Yes | No | Yes | ICR | 20-30 | No | No | Yes | 5 | 4 | No | Yes | I.V. | 0.25 | 48 | No | No | No | Probit analysis | mcl |
| Tan CH, 2015 (40) | Yes | No | Yes | Yes | Yes | Yes | ICR | 20-25 | No | No | Yes | 2.5, 5 | 4 | No | Yes | I.V. | 0.2 | 48 | No | No | Yes | Probit analysis | mcl |
| Leong PK, 2015 (41) | Yes | No | Yes | Yes | No | Yes | ICR | 20-25 | No | No | Yes | 5 | 4 | No | Yes | I.V. | 0.2 | 48 | No | No | Yes | Probit analysis | mcl |
| Leong PK, 2014 (42) | Yes | Yes | Both | Yes | No | Yes | ICR | 20-30 | No | No | Yes | 2.5, 5 | 4 | No | Yes | I.V. | 0.25 | 48 | No | No | Yes | Probit analysis | mcl |
| Danpaiboon W, 2014 (43) | No | No | Yes | Yes | Yes | Yes | ICR | No | Yes (Male) | No | Yes | 1.5 | 5 | No | Yes | I.P. | 0.5 | 48 | No | No | No | N/A | mcg/g |
| Pakmanee N, 2013 (44) | No | No | Yes | Yes | No | Yes | ICR | 18-20 | No | No | Yes | 2 | 5 | No | Yes | I.V. | 0.2 | 24 | No | No | Yes | Method of Theakston and Reid | mg/ml |
| Leong PK, 2012 (45) | Yes | Yes | Both | Yes | Yes | Yes | ICR | 20-25 | No | No | Yes | 2.5, 5 | No | No | Yes | I.V. | 0.25 | 48 | No | No | Yes | Probit analysis | mcl |
| Leong PK, 2012 (46) | Yes | Yes | Both | Yes | Yes | Yes | ICR | 20-25 | No | No | Yes | 2.5, 5 | 4 | No | Yes | I.V. | 0.25 | 48 | No | No | Yes | Probit analysis | mcl |
| Tan CH, 2011 (47) | Yes | No | Yes | Yes | No | Yes | ICR | 16-25 | No | No | Yes | 5 | 4 | No | Yes | I.V. | 0.3 | 48 | No | No | Yes | Probit analysis | mcl |
| Chanhome L, 2002 (48) | Yes | No | Yes | No | No | No | Swiss | 18-20 | No | No | Yes | 4 | 4 | No | Yes | I.V. | No | No | No | No | No | Method of Reed and Muench | mcl |
| Khow O, 2001 (49) | Yes | No | Yes | No | No | No | No | No | No | No | Yes | 4 | 4 | No | Yes | I.V. | 0.2 | 24 | No | No | No | Method of Reed and Muench | mcl |
| Chanhome L, 1999 (50) | Yes | No | Yes | No | No | No | Swiss | 18-20 | No | No | Yes | 4 | 4 | No | Yes | I.V. | 0.5 | No | No | No | No | Method of Reed and Muench | mcl |
| Khow O, 1997 (51) | Yes | No | Yes | No | No | No | No | 18-20 | No | No | Yes | 4 | 3 | No | Yes | I.V. | 0.2 | 24 | No | No | No | Method of Reed and Muench | mcl |
| Sells PG, 1994 (52) | No | No | Yes | No | No | No | No | 18-20 | No | No | Yes | 5 | 5 | No | Yes | I.V. | 0.2 | 24 | No | No | No | Probit analysis | mg |
| **Number of fulfilling studies** | **47** | **31** | **48** | **45** | **39** | **36** | **49** | **49** | **10** | **0** | **11** | **52** | **49** | **0** | **52** | **52** | **40** | **50** | **0** | **0** | **28** | **49** | **52** |
| **Percent-age (%)** | **90** | **60** | **98** | **87** | **75** | **69** | **94** | **94** | **19** | **0** | **21** | **100** | **94** | **0** | **100** | **100** | **77** | **96** | **0** | **0** | **54** | **94** | **100** |

**References**

1. Tan KY, Shamsuddin NN, Tan CH. Sharp-nosed Pit Viper (Deinagkistrodon acutus) from Taiwan and China: A comparative study on venom toxicity and neutralization by two specific antivenoms across the Strait. Acta Trop. 2022;232:106495.

2. Chanhome L, Khow O, Reamtong O, Vasaruchapong T, Laoungbua P, Tawan T, et al. Biochemical and proteomic analyses of venom from a new pit viper, Protobothrops kelomohy. Journal of Venomous Animals and Toxins Including Tropical Diseases. 2022;28:14.

3. Wong KY, Tan KY, Tan NH, Gnanathasan CA, Tan CH. Elucidating the Venom Diversity in Sri Lankan Spectacled Cobra (Naja naja) through De Novo Venom Gland Transcriptomics, Venom Proteomics and Toxicity Neutralization. Toxins. 2021;13(8):30.

4. Faisal T, Tan KY, Tan NH, Sim SM, Gnanathasan CA, Tan CH. Proteomics, toxicity and antivenom neutralization of Sri Lankan and Indian Russell's viper (Daboia russelii) venoms. Journal of Venomous Animals and Toxins Including Tropical Diseases. 2021;27:15.

5. Attarde S, Khochare S, Iyer A, Dam P, Martin G, Sunagar K. Venomics of the Enigmatic Andaman Cobra (Naja sagittifera) and the Preclinical Failure of Indian Antivenoms in Andaman and Nicobar Islands. Frontiers in Pharmacology. 2021;12:16.

6. Tan CH, Palasuberniam P, Blanco FB, Tan KY. Immunoreactivity and neutralization capacity of Philippine cobra antivenom against Naja philippinensis and Naja samarensis venoms. Transactions of the Royal Society of Tropical Medicine and Hygiene. 2021;115(1):78-84.

7. Oh AMF, Tan KY, Tan NH, Tan CH. Proteomics and neutralization of Bungarus multicinctus (Many-banded Krait) venom: Intra-specific comparisons between specimens from China and Taiwan. Comparative Biochemistry and Physiology Part - C: Toxicology and Pharmacology. 2021;247.

8. Laxme RRS, Khochare S, Attarde S, Suranse V, Iyer A, Casewell NR, et al. Biogeographic venom variation in Russell's viper (Daboia russelii) and the preclinical inefficacy of antivenom therapy in snakebite hotspots. Plos Neglected Tropical Diseases. 2021;15(3).

9. Laxme RRS, Attarde S, Khochare S, Suranse V, Martin G, Casewell NR, et al. Biogeographical venom variation in the Indian spectacled cobra (Naja naja) underscores the pressing need for pan-India efficacious snakebite therapy. Plos Neglected Tropical Diseases. 2021;15(2).

10. Yee KT, Maw LZ, Kyaw AM, Khow O, Oo AW, Oo TKK, et al. Evaluation of the cross-neutralization capacity of Thai green pit viper antivenom against venom of Myanmar green pit viper. Toxicon. 2020;177:41-5.

11. Tan KY, Ng TS, Bourges A, Ismail AK, Maharani T, Khomvilai S, et al. Geographical variations in king cobra (Ophiophagus hannah) venom from Thailand, Malaysia, Indonesia and China: On venom lethality, antivenom immunoreactivity and in vivo neutralization. Acta Tropica. 2020;203.

12. Lin B, Zhang JR, Lu HJ, Zhao L, Chen J, Zhang HF, et al. Immunoreactivity and neutralization study of chinese bungarus multicinctus antivenin and lab-prepared anti-bungarotoxin antisera towards purified bungarotoxins and snake venoms. PLoS Neglected Tropical Diseases. 2020;14(11):1-19.

13. Liew JL, Tan NH, Tan CH. Proteomics and preclinical antivenom neutralization of the mangrove pit viper (Trimeresurus purpureomaculatus, Malaysia) and white-lipped pit viper (Trimeresurus albolabris, Thailand) venoms. Acta Tropica. 2020;209.

14. Lee LP, Tan KY, Tan CH. Toxicity and cross-neutralization of snake venoms from two lesser-known arboreal pit vipers in Southeast Asia: Trimeresurus wiroti and Trimeresurus puniceus. Toxicon. 2020;185:91-6.

15. Hia YL, Tan KY, Tan CH. Comparative venom proteomics of banded krait (Bungarus fasciatus) from five geographical locales: Correlation of venom lethality, immunoreactivity and antivenom neutralization. Acta Tropica. 2020;207.

16. Choraria A, Somasundaram R, Gautam M, Ramanathan M, Paray BA, Al-Sadoon MK, et al. Experimental antivenoms from chickens and rabbits and their comparison with commercially available equine antivenom against the venoms of Daboia russelii and Echis carinatus snakes. Toxin Reviews. 2020.

17. Tan CH, Tan KY, Ng TS, Quah ESH, Ismail AK, Khomvilai S, et al. Venomics of trimeresurus (Popeia) nebularis, the cameron highlands pit viper from Malaysia: Insights into venom proteome, toxicity and neutralization of antivenom. Toxins. 2019;11(2).

18. Pla D, Sanz L, Quesada-Bernat S, Villalta M, Baal J, Chowdhury MAW, et al. Phylovenomics of Daboia russelii across the Indian subcontinent. Bioactivities and comparative in vivo neutralization and in vitro third-generation antivenomics of antivenoms against venoms from India, Bangladesh and Sri Lanka. Journal of Proteomics. 2019;207.

19. Oh AMF, Tan CH, Tan KY, Quraishi NH, Tan NH. Venom proteome of Bungarus sindanus (Sind krait) from Pakistan and in vivo cross-neutralization of toxicity using an Indian polyvalent antivenom. Journal of Proteomics. 2019;193:243-54.

20. Lingam TMC, Tan KY, Tan CH. Thai Russell's viper monospecific antivenom is immunoreactive and effective in neutralizing the venom of Daboia siamensis from Java, Indonesia. Toxicon. 2019;168:95-7.

21. Laxme RRS, Khochare S, de Souza HF, Ahuja B, Suranse V, Martin G, et al. Beyond the 'big four': Venom profiling of the medically important yet neglected Indian snakes reveals disturbing antivenom deficiencies. Plos Neglected Tropical Diseases. 2019;13(12).

22. Deka A, Abu Reza M, Hoque KMF, Deka K, Saha S, Doley R. Comparative analysis of Naja kaouthia venom from North-East India and Bangladesh and its cross reactivity with Indian polyvalent antivenoms. Toxicon. 2019;164:31-43.

23. Chaisakul J, Alsolaiss J, Charoenpitakchai M, Wiwatwarayos K, Sookprasert N, Harrison RA, et al. Evaluation of the geographical utility of Eastern Russell’s viper (Daboia siamensis) antivenom from Thailand and an assessment of its protective effects against venom-induced nephrotoxicity. PLoS Neglected Tropical Diseases. 2019;13(10).

24. Tan CH, Tan KY, Ng TS, Sim SM, Tan NH. Venom Proteome of Spine-Bellied Sea Snake (Hydrophis curtus) from Penang, Malaysia: Toxicity Correlation, Immunoprofiling and Cross-Neutralization by Sea Snake Antivenom. Toxins (Basel). 2018;11(1).

25. Tan KY, Tan NH, Tan CH. Venom proteomics and antivenom neutralization for the Chinese eastern Russell's viper, Daboia siamensis from Guangxi and Taiwan. Scientific reports. 2018;8(1):8545.

26. Sanz L, Quesada-Bernat S, Chen PY, Lee CD, Chiang JR, Calvete JJ. Translational Venomics: Third-Generation Antivenomics of Anti-Siamese Russell's Viper, Daboia siamensis, Antivenom Manufactured in Taiwan CDC's Vaccine Center. Trop Med Infect Dis. 2018;3(2).

27. Liu BS, Wu WG, Lin MH, Li CH, Jiang BR, Wu SC, et al. Identification of immunoreactive peptides of toxins to simultaneously assess the neutralization potency of antivenoms against neurotoxicity and cytotoxicity of Naja atra venom. Toxins. 2018;10(1).

28. Faisal T, Tan KY, Sim SM, Quraishi N, Tan NH, Tan CH. Proteomics, functional characterization and antivenom neutralization of the venom of Pakistani Russell's viper (Daboia russelii) from the wild. Journal of Proteomics. 2018;183:1-13.

29. Tan CH, Liew JL, Tan NH, Ismail AK, Maharani T, Khomvilai S, et al. Cross reactivity and lethality neutralization of venoms of Indonesian Trimeresurus complex species by Thai Green Pit Viper Antivenom. Toxicon. 2017;140:32-7.

30. Tan CH, Wong KY, Tan KY, Tan NH. Venom proteome of the yellow-lipped sea krait, Laticauda colubrina from Bali: Insights into subvenomic diversity, venom antigenicity and cross-neutralization by antivenom. J Proteomics. 2017;166:48-58.

31. Oh AMF, Tan CH, Ariaranee GC, Quraishi N, Tan NH. Venomics of Bungarus caeruleus (Indian krait): Comparable venom profiles, variable immunoreactivities among specimens from Sri Lanka, India and Pakistan. Journal of Proteomics. 2017;164:1-18.

32. Wong KY, Tan CH, Tan NH. Venom and purified toxins of the spectacled cobra (Naja naja) from Pakistan: Insights into toxicity and antivenom neutralization. American Journal of Tropical Medicine and Hygiene. 2016;94(6):1392-9.

33. Villalta M, Sánchez A, Herrera M, Vargas M, Segura Á, Cerdas M, et al. Development of a new polyspecific antivenom for snakebite envenoming in Sri Lanka: Analysis of its preclinical efficacy as compared to a currently available antivenom. Toxicon. 2016;122:152-9.

34. Tan KY, Tan CH, Fung SY, Tan NH. Neutralization of the principal toxins from the venoms of thai naja kaouthia and malaysian hydrophis schistosus: Insights into toxin-specific neutralization by two different antivenoms. Toxins. 2016;8(4).

35. Tan CH, Liew JL, Tan KY, Tan NH. Assessing SABU (Serum Anti Bisa Ular), the sole Indonesian antivenom: A proteomic analysis and neutralization efficacy study. Scientific reports. 2016;6:37299.

36. Tan CH, Tan KY, Tan NH. Revisiting Notechis scutatus venom: on shotgun proteomics and neutralization by the "bivalent" Sea Snake Antivenom. J Proteomics. 2016;144:33-8.

37. Maduwage K, Silva A, O'Leary MA, Hodgson WC, Isbister GK. Efficacy of Indian polyvalent snake antivenoms against Sri Lankan snake venoms: lethality studies or clinically focussed in vitro studies. Scientific reports. 2016;6:26778.

38. Yap MK, Tan NH, Sim SM, Fung SY, Tan CH. The Effect of a Polyvalent Antivenom on the Serum Venom Antigen Levels of Naja sputatrix (Javan Spitting Cobra) Venom in Experimentally Envenomed Rabbits. Basic Clin Pharmacol Toxicol. 2015;117(4):274-9.

39. Tan KY, Tan CH, Fung SY, Tan NH. Venomics, lethality and neutralization of Naja kaouthia (monocled cobra) venoms from three different geographical regions of Southeast Asia. Journal of Proteomics. 2015;120:105-25.

40. Tan CH, Tan NH, Tan KY, Kwong KO. Antivenom cross-neutralization of the venoms of Hydrophis schistosus and Hydrophis curtus, two common sea snakes in Malaysian waters. Toxins. 2015;7(2):572-81.

41. Leong PK, Fung SY, Tan CH, Sim SM, Tan NH. Immunological cross-reactivity and neutralization of the principal toxins of Naja sumatrana and related cobra venoms by a Thai polyvalent antivenom (Neuro Polyvalent Snake Antivenom). Acta Tropica. 2015;149:86-93.

42. Leong PK, Tan CH, Sim SM, Fung SY, Sumana K, Sitprija V, et al. Cross neutralization of common Southeast Asian viperid venoms by a Thai polyvalent snake antivenom (Hemato Polyvalent Snake Antivenom). Acta Tropica. 2014;132(1):7-14.

43. Danpaiboon W, Reamtong O, Sookrung N, Seesuay W, Sakolvaree Y, Thanongsaksrikul J, et al. Ophiophagus hannah venom: Proteome, components bound by Naja kaouthia antivenin and neutralization by n. kaouthia neurotoxin-specific human ScFv. Toxins. 2014;6(5):1526-58.

44. Pakmanee N, Noiphrom J, Kay A, Pornmuttakun D, Sakolparp L, Hemmala W, et al. Comparative abilities of IgG and F(ab ')(2) monovalent antivenoms to neutralize lethality, phospholipase A(2), and coagulant activities induced by Daboia siamensis venom and their anticomplementary activity. Scienceasia. 2013;39(2):160-6.

45. Leong PK, Tan NH, Fung SY, Sim SM. Cross neutralisation of Southeast Asian cobra and krait venoms by Indian polyvalent antivenoms. Transactions of the Royal Society of Tropical Medicine and Hygiene. 2012;106(12):731-7.

46. Leong PK, Sim SM, Fung SY, Sumana K, Sitprija V, Tan NH. Cross neutralization of afro-asian cobra and asian krait venoms by a thai polyvalent snake antivenom (neuro polyvalent snake antivenom). PLoS Neglected Tropical Diseases. 2012;6(6).

47. Tan CH, Leong PK, Fung SY, Sim SM, Ponnudurai G, Ariaratnam C, et al. Cross neutralization of Hypnale hypnale (hump-nosed pit viper) venom by polyvalent and monovalent Malayan pit viper antivenoms in vitro and in a rodent model. Acta Tropica. 2011;117(2):119-24.

48. Chanhome L, Khow O, Omori-Satoh T, Sitprija V. Capacity of Thai green pit viper antivenom to neutralize the venoms of Thai Trimeresurus snakes and comparison of biological activities of these venoms. Journal of natural toxins. 2002;11(3):251-9.

49. Khow O, Chanhome L, Omori-Satoh T, Sitprija V. Effectiveness of Thai cobra (Naja kaouthia) antivenom against sea snake (Lapemis hardwickii) venom: verification by affinity purified F(AB')2 fragments. J Nat Toxins. 2001;10(3):249-53.

50. Chanhome L, Wongtongkam N, Khow O, Pakmanee N, Omori-Satoh T, Sitprija V. Genus specific neutralization of Bungarus snake venoms by Thai Red Cross banded krait antivenom. Journal of Natural Toxins. 1999;8(1):135-40.

51. Khow O, Pakmanee N, Chanhome L, Sriprapat S, Omori-Satoh T, Sitprija V. Cross-neutralization of Thai cobra (Naja kaouthia) and spitting cobra (Naja siamensis) venoms by Thai cobra antivenom. Toxicon. 1997;35(11):1649-51.

52. Sells PG, Jones RG, Laing GD, Smith DC, Theakston RD. Experimental evaluation of ovine antisera to Thai cobra (Naja kaouthia) venom and its alpha-neurotoxin. Toxicon. 1994;32(12):1657-65.
