# Supplementary material for: Antivenom preclinical efficacy testing against Asian snakes and their availability in Asia: A systematic review
Source: PLoS One. 2023 Jul 19;18(7):e0288723. doi: 10.1371/journal.pone.0288723 (PMC10355433; doi:10.1371/journal.pone.0288723)
Supplement: S7 Table — (DOCX) [file pone.0288723.s007.docx]

# **S7 Table. Excluded studies with reasons from the search in this review.**

| **Reasons for exclusion** | **References** |
| --- | --- |
| Not a study of *in vivo* neutralization of lethality of snake venom (n = 177) | 1. Thakur S, Malhotra A, Giri S, Lalremsanga HT, Bharti OK, Santra V, et al. Venom of several Indian green pit vipers: Comparison of biochemical activities and cross-reactivity with antivenoms. Toxicon. 2022;210:66-77. 2. Thakshila P, Hodgson WC, Isbister GK, Silva A. In Vitro Neutralization of the Myotoxicity of Australian Mulga Snake (Pseudechis australis) and Sri Lankan Russell's Viper (Daboia russelii) Venoms by Australian and Indian Polyvalent Antivenoms. Toxins (Basel). 2022;14(5). 3. Laustsen AH, Gless BH, Jenkins TP, Meyhoff-Madsen M, Bjärtun J, Munk AS, et al. In Vivo Neutralization of Myotoxin II, a Phospholipase A2Homologue from Bothrops asper Venom, Using Peptides Discovered via Phage Display Technology. ACS Omega. 2022. 4. Huynh TM, Hodgson WC, Isbister GK, Silva A. The Effect of Australian and Asian Commercial Antivenoms in Reversing the Post-Synaptic Neurotoxicity of O. hannah, N. naja and N. kaouthia Venoms In Vitro. Toxins. 2022;14(4):10. 5. Chong HP, Tan KY, Liu BS, Sung WC, Tan CH. Cytotoxicity of Venoms and Cytotoxins from Asiatic Cobras (Naja kaouthia, Naja sumatrana, Naja atra) and Neutralization by Antivenoms from Thailand, Vietnam, and Taiwan. Toxins (Basel). 2022;14(5). 6. Yong MY, Tan KY, Tan CH. Potential para-specific and geographical utility of Thai Green Pit Viper (Trimeresurus albolabris) Monovalent Antivenom: Neutralization of procoagulant and hemorrhagic activities of diverse Trimeresurus pit viper venoms. Toxicon. 2021;203:85-92. 7. Tan CH, Palasuberniam P, Tan KY. Snake Venom Proteomics, Immunoreactivity and Toxicity Neutralization Studies for the Asiatic Mountain Pit Vipers, Ovophis convictus, Ovophis tonkinensis, and Hime Habu, Ovophis okinavensis. Toxins. 2021;13(8):18. 8. Rodrigues CFB, Zdenek CN, Bourke LA, Seneci L, Chowdhury A, Freitas-de-Sousa LA, et al. Clinical implications of ontogenetic differences in the coagulotoxic activity of Bothrops jararacussu venoms. Toxicol Lett. 2021;348:59-72. 9. Muniz EG, Noronha MDDN, Saraiva MDGG, Monteiro WM, Oliveira SS. Neutralization of hemostatic disorders induced by Lachesis muta venom using Brazilian antivenoms. Toxicon. 2021;191:44-7. 10. Lingam TMC, Tan KY, Tan CH. Capillary leak syndrome induced by the venoms of Russell's Vipers (Daboia russelii and Daboia siamensis) from eight locales and neutralization of the differential toxicity by three snake antivenoms. Comp Biochem Physiol C-Toxicol Pharmacol. 2021;250:6. 11. Ho CH, Chiang LC, Mao YC, Lan KC, Tsai SH, Shih YJ, et al. Analysis of the Necrosis-Inducing Components of the Venom of Naja atra and Assessment of the Neutralization Ability of Freeze-Dried Antivenom. Toxins (Basel). 2021;13(9). 12. Chowdhury A, Zdenek CN, Lewin MR, Carter R, Jagar T, Ostanek E, et al. Venom-Induced Blood Disturbances by Palearctic Viperid Snakes, and Their Relative Neutralization by Antivenoms and Enzyme-Inhibitors. Front Immunol. 2021;12:14. 13. Madhushani U, Isbister GK, Tasoulis T, Hodgson WC, Silva A. In-Vitro Neutralization of the Neurotoxicity of Coastal Taipan Venom by Australian Polyvalent Antivenom: The Window of Opportunity. Toxins (Basel). 2020;12(11). 14. Szteiter SS, Diego IN, Ortegon J, Salinas EM, Cirilo A, Reyes A, et al. Examination of the Efficacy and Cross-Reactivity of a Novel Polyclonal Antibody Targeting the Disintegrin Domain in SVMPs to Neutralize Snake Venom. Toxins. 2021;13(4). 15. Muniz EG, Noronha M, Saraiva M, Monteiro WM, Oliveira SS. Neutralization of hemostatic disorders induced by Lachesis muta venom using Brazilian antivenoms. Toxicon. 2021;191:44-7. 16. Liang Q, Huynh TM, Ng YZ, Isbister GK, Hodgson WC. In Vitro Neurotoxicity of Chinese Krait (Bungarus multicinctus) Venom and Neutralization by Antivenoms. Toxins. 2021;13(1). 17. Dashevsky D, Benard-Valle M, Neri-Castro E, Youngman NJ, Zdenek CN, Alagon A, et al. Anticoagulant Micrurus venoms: Targets and neutralization. Toxicology Letters. 2021;337:91-7. 18. Chowdhury A, Zdenek CN, Dobson JS, Bourke LA, Soria R, Fry BG. Clinical implications of differential procoagulant toxicity of the palearctic viperid genus Macrovipera, and the relative neutralization efficacy of antivenoms and enzyme inhibitors. Toxicology Letters. 2021;340:77-88. 19. Bourke LA, Zdenek CN, Neri-Castro E, Bénard-Valle M, Alagón A, Gutiérrez JM, et al. Pan-American Lancehead Pit-Vipers: Coagulotoxic Venom Effects and Antivenom Neutralisation of Bothrops asper and B. atrox Geographical Variants. Toxins. 2021;13(2). 20. Zdenek CN, Youngman NJ, Hay C, Dobson J, Dunstan N, Allen L, et al. Anticoagulant activity of black snake (Elapidae: Pseudechis) venoms: Mechanisms, potency, and antivenom efficacy. Toxicology Letters. 2020;330:176-84. 21. Xie CF, Slagboom J, Albulescu LO, Bruyneel B, Still KBM, Vonk FJ, et al. Antivenom Neutralization of Coagulopathic Snake Venom Toxins Assessed by Bioactivity Profiling Using Nanofractionation Analytics. Toxins. 2020;12(1). 22. Souza JB, Cardoso R, Almeida-Souza HO, Carvalho CP, Correia LIV, Faria PCB, et al. Generation and In-planta expression of a recombinant single chain antibody with broad neutralization activity on Bothrops pauloensis snake venom. International Journal of Biological Macromolecules. 2020;149:1241-51. 23. Sousa LF, Bernardoni JL, Zdenek CN, Dobson J, Coimbra F, Gillett A, et al. Differential coagulotoxicity of metalloprotease isoforms from Bothrops neuwiedi snake venom and consequent variations in antivenom efficacy. Toxicology Letters. 2020;333:211-21. 24. Patra A, Mukherjee AK. Proteomic Analysis of Sri Lanka Echis carinatus Venom: Immunological Cross-Reactivity and Enzyme Neutralization Potency of Indian Polyantivenom. Journal of Proteome Research. 2020;19(8):3022-32. 25. Madhushani U, Isbister GK, Tasoulis T, Hodgson WC, Silva A. In-Vitro Neutralization of the Neurotoxicity of Coastal Taipan Venom by Australian Polyvalent Antivenom: The Window of Opportunity. Toxins. 2020;12(11). 26. Lingam TMC, Tan KY, Tan CH. Proteomics and antivenom immunoprofiling of Russell's viper (Daboia siamensis) venoms from Thailand and Indonesia. Journal of Venomous Animals and Toxins Including Tropical Diseases. 2020;26. 27. Liang Q, Huynh TM, Konstantakopoulos N, Isbister GK, Hodgson WC. An Examination of the Neutralization of In Vitro Toxicity of Chinese Cobra (Naja atra) Venom by Different Antivenoms. Biomedicines. 2020;8(10). 28. Hashmi SU, Alvi A, Munir I, Perveen M, Fazal A, Jackson TNW, et al. Functional venomics of the Big-4 snakes of Pakistan. Toxicon. 2020;179:60-71. 29. García B, Neri E, Bénard M, Zamundio F, Ocampo L, Morales E, et al. Characterization and neutralization of local damage caused by the complete venom of Atropoides nummifer and its main myotoxin in a murine model. Toxicon. 2020;182:S6-S7. 30. Chanda A, Mukherjee AK. Quantitative proteomics to reveal the composition of Southern India spectacled cobra (Naja naja) venom and its immunological cross-reactivity towards commercial antivenom. International Journal of Biological Macromolecules. 2020;160:224-32. 31. Chaisakul J, Rusmili MRA, Alsolaiss J, Albulescu LO, Harrison RA, Othman I, et al. In Vitro Immunological Cross-Reactivity of Thai Polyvalent and Monovalent Antivenoms with Asian Viper Venoms. Toxins. 2020;12(12). 32. Campos LB, Pucca MB, Silva LC, Pessenda G, Filardi BA, Cerni FA, et al. Identification of cross-reactive human single-chain variable fragments against phospholipases A2 from Lachesis muta and Bothrops spp venoms. Toxicon. 2020;184:116-21. 33. Bourke LA, Youngman NJ, Zdenek CN, op den Brouw B, Violette A, Fourmy R, et al. Trimeresurus albolabris snakebite treatment implications arising from ontogenetic venom comparisons of anticoagulant function, and antivenom efficacy. Toxicology Letters. 2020;327:2-8. 34. Zdenek CN, op den Brouw B, Dashevsky D, Gloria A, Youngman NJ, Watson E, et al. Clinical implications of convergent procoagulant toxicity and differential antivenom efficacy in Australian elapid snake venoms. Toxicology Letters. 2019;316:171-82. 35. Zdenek CN, Hay C, Arbuckle K, Jackson TNW, Bos MHA, op den Brouw B, et al. Coagulotoxic effects by brown snake (Pseudonaja) and taipan (Oxyuranus) venoms, and the efficacy of a new antivenom. Toxicology in Vitro. 2019;58:97-109. 36. Youngman NJ, Zdenek CN, Dobson JS, Bittenbinder MA, Gillett A, Hamilton B, et al. Mud in the blood: Novel potent anticoagulant coagulotoxicity in the venoms of the Australian elapid snake genus Denisonia (mud adders) and relative antivenom efficacy. Toxicology Letters. 2019;302:1-6. 37. Youngman NJ, Debono J, Dobson JS, Zdenek CN, Harris RJ, Op den Brouw B, et al. Venomous Landmines: Clinical Implications of Extreme Coagulotoxic Diversification and Differential Neutralization by Antivenom of Venoms within the Viperid Snake Genus Bitis. Toxins (Basel). 2019;11(7). 38. Tang ELH, Tan NH, Fung SY, Tan CH. Comparative proteomes, immunoreactivities and neutralization of procoagulant activities of Calloselasma rhodostoma (Malayan pit viper) venoms from four regions in Southeast Asia. Toxicon. 2019;169:91-102. 39. Kuniyoshi AK, Kodama RT, Cajado-Carvalho D, Iwai LK, Kitano E, da Silva CCF, et al. Experimental antivenom against serine proteases from the Bothrops jararaca venom obtained in mice, and its comparison with the antibothropic serum from the Butantan Institute. Toxicon. 2019;169:59-67. 40. Gomes M, Alvarez MA, Quellis LR, Becher ML, Castro JMA, Gameiro J, et al. Expression of an scFv antibody fragment in Nicotiana benthamiana and in vitro assessment of its neutralizing potential against the snake venom metalloproteinase BaP1 from Bothrops asper. Toxicon. 2019;160:38-46. 41. Fry B. Clinical implications of antivenom failures to neutralise coagulotoxicity of African snake venoms. Research and Practice in Thrombosis and Haemostasis. 2019;3:64-5. 42. Floriano RS, Schezaro-Ramos R, Silva NJ, Bucaretchi F, Rowan EG, Hyslop S. Neurotoxicity of Micrurus lemniscatus lemniscatus (South American coralsnake) venom in vertebrate neuromuscular preparations in vitro and neutralization by antivenom. Archives of Toxicology. 2019;93(7):2065-86. 43. Debono J, Bos MHA, Frank N, Fry B. Clinical implications of differential antivenom efficacy in neutralising coagulotoxicity produced by venoms from species within the arboreal viperid snake genus Trimeresurus. Toxicology Letters. 2019;316:35-48. 44. Zanetti G, Duregotti E, Locatelli CA, Giampreti A, Lonati D, Rossetto O, et al. Variability in venom composition of European viper subspecies limits the cross-effectiveness of antivenoms. Sci Rep. 2018;8(1):9818. 45. Patra A, Kalita B, Mukherjee AK. Assessment of quality, safety, and pre-clinical toxicity of an equine polyvalent anti-snake venom (Pan Africa): Determination of immunological cross-reactivity of antivenom against venom samples of Elapidae and Viperidae snakes of Africa. Toxicon. 2018;153:120-7. 46. Oulion B, Dobson JS, Zdenek CN, Arbuckle K, Lister C, Coimbra FCP, et al. Factor X activating Atractaspis snake venoms and the relative coagulotoxicity neutralising efficacy of African antivenoms. Toxicology Letters. 2018;288:119-28. 47. Luiz MB, Pereira SS, Prado NDR, Goncalves NR, Kayano AM, Moreira-Dill LS, et al. Camelid Single-Domain Antibodies (VHHs) against Crotoxin: A Basis for Developing Modular Building Blocks for the Enhancement of Treatment or Diagnosis of Crotalic Envenoming. Toxins. 2018;10(4). 48. Laustsen AH, Karatt-Vellatt A, Masters EW, Arias AS, Pus U, Knudsen C, et al. In vivo neutralization of dendrotoxin-mediated neurotoxicity of black mamba venom by oligoclonal human IgG antibodies. Nature Communications. 2018;9(1). 49. Khanongnoi J, Phanthong S, Reamtong O, Tungtronchitr A, Chaicumpa W, Sookrung N. Human monoclonal scfvs that neutralize fribrinogenolytic activity of kaouthiagin, a zinc-metalloproteinase in cobra (Naja kaouthia) venom. Toxins. 2018;10(12). 50. Kalita B, Singh S, Patra A, Mukherjee AK. Quantitative proteomic analysis and antivenom study revealing that neurotoxic phospholipase A2 enzymes, the major toxin class of Russell's viper venom from southern India, shows the least immuno-recognition and neutralization by commercial polyvalent antivenom. International Journal of Biological Macromolecules. 2018;118:375-85. 51. Chanda A, Patra A, Kalita B, Mukherjee AK. Proteomics analysis to compare the venom composition between Naja naja and Naja kaouthia from the same geographical location of eastern India: Correlation with pathophysiology of envenomation and immunological cross-reactivity towards commercial polyantivenom. Expert Review of Proteomics. 2018;15(11):949-61. 52. Ainsworth S, Slagboom J, Alomran N, Pla D, Alhamdi Y, King SI, et al. The paraspecific neutralisation of snake venom induced coagulopathy by antivenoms. Commun Biol. 2018;1:34. 53. Yang DC, Dobson J, Cochran C, Dashevsky D, Arbuckle K, Benard M, et al. The Bold and the Beautiful: a Neurotoxicity Comparison of New World Coral Snakes in the Micruroides and Micrurus Genera and Relative Neutralization by Antivenom. Neurotoxicity Research. 2017;32(3):487-95. 54. Rogalski A, Soerensen C, op den Brouw B, Lister C, Dashevsky D, Arbuckle K, et al. Differential procoagulant effects of saw-scaled viper (Serpentes: Viperidae: Echis) snake venoms on human plasma and the narrow taxonomic ranges of antivenom efficacies. Toxicology Letters. 2017;280:159-70. 55. Melo LL, Mendes MM, Alves LM, Isabel TF, Vieira S, Gimenes SNC, et al. Cross-reactivity and inhibition myotoxic effects induced by Bothrops snake venoms using specific polyclonal anti-BnSP7 antibodies. Biologicals. 2017;50:109-16. 56. Lister C, Arbuckle K, Jackson TNW, Debono J, Zdenek CN, Dashevsky D, et al. Catch a tiger snake by its tail: Differential toxicity, co-factor dependence and antivenom efficacy in a procoagulant clade of Australian venomous snakes. Comparative Biochemistry and Physiology Part - C: Toxicology and Pharmacology. 2017;202:39-54. 57. Lam SK, Yip SF, Crow P, Fung HT, Cheng JM, Tan KS, et al. Comparison of green pit viper and Agkistrodon halys antivenom in inhibition of coagulopathy due to Trimeresurus albolabris venom: an in-vitro study using human plasma. Hong Kong Med J. 2017;23(1):13-8. 58. Kuniyoshi AK, Kodama RT, Moraes LHF, Duzzi B, Iwai LK, Lima IF, et al. In vitro cleavage of bioactive peptides by peptidases from Bothrops jararaca venom and its neutralization by bothropic antivenom produced by Butantan Institute: Major contribution of serine peptidases. Toxicon. 2017;137:114-9. 59. Kalita B, Patra A, Mukherjee AK. Unraveling the Proteome Composition and Immuno-profiling of Western India Russell's Viper Venom for In-Depth Understanding of Its Pharmacological Properties, Clinical Manifestations, and Effective Antivenom Treatment. Journal of Proteome Research. 2017;16(2):583-98. 60. Félix-Silva J, Gomes JA, Xavier-Santos JB, Passos JG, Silva-Junior AA, Tambourgi DV, et al. Inhibition of local effects induced by Bothrops erythromelas snake venom: Assessment of the effectiveness of Brazilian polyvalent bothropic antivenom and aqueous leaf extract of Jatropha gossypiifolia. Toxicon. 2017;125:74-83. 61. Collaço RC, Randazzo-Moura P, Tamascia ML, da Silva IR, Rocha T, Cogo JC, et al. Bothrops fonsecai snake venom activities and cross-reactivity with commercial bothropic venom. Comp Biochem Physiol C Toxicol Pharmacol. 2017;191:86-100. 62. Barreto G, de Oliveira SS, dos Anjos IV, Chalkidis HD, Mourao RHV, Moura-da-Silva AM, et al. Experimental Bothrops atrox envenomation: Efficacy of antivenom therapy and the combination of Bothrops antivenom with dexamethasone. Plos Neglected Tropical Diseases. 2017;11(3). 63. Tan KY, Tan CH, Sim SM, Fung SY, Tan NH. Geographical venom variations of the Southeast Asian monocled cobra (Naja kaouthia): venom-induced neuromuscular depression and antivenom neutralization. Comp Biochem Physiol C Toxicol Pharmacol. 2016;185-186:77-86. 64. Sintiprungrat K, Watcharatanyatip K, Senevirathne W, Chaisuriya P, Chokchaichamnankit D, Srisomsap C, et al. A comparative study of venomics of Naja naja from India and Sri Lanka, clinical manifestations and antivenomics of an Indian polyspecific antivenom. Journal of Proteomics. 2016;132:131-43. 65. Silva A, Hodgson WC, Isbister GK. Cross-neutralisation of in vitro neurotoxicity of asian and Australian snake neurotoxins and venoms by different antivenoms. Toxins. 2016;8(10). 66. Mukherjee AK, Kalita B, Mackessy SP. A proteomic analysis of Pakistan Daboia russelii russelii venom and assessment of potency of Indian polyvalent and monovalent antivenom. Journal of Proteomics. 2016;144:73-86. 67. Maduwage KP, Scorgie FE, Lincz LF, O'Leary MA, Isbister GK. Procoagulant snake venoms have differential effects in animal plasmas: Implications for antivenom testing in animal models. Thrombosis Research. 2016;137:174-7. 68. Boumaiza S, Oussedik-Oumehdi H, Laraba-Djebari F. Pathophysiological effects of Cerastes cerastes and Vipera lebetina venoms: Immunoneutralization using anti-native and anti-60Co irradiated venoms. Biologicals. 2016;44(1):1-11. 69. Paixão-Cavalcante D, Kuniyoshi AK, Portaro FC, da Silva WD, Tambourgi DV. African adders: partial characterization of snake venoms from three Bitis species of medical importance and their neutralization by experimental equine antivenoms. PLoS Negl Trop Dis. 2015;9(2):e0003419. 70. Sharma M, Gogoi N, Dhananjaya BL, Menon JC, Doley R. Geographical variation of Indian Russell's viper venom and neutralization of its coagulopathy by polyvalent antivenom. Toxin Reviews. 2014;33(1-2):7-15. 71. Lanari LC, Alagón A, Costa de Oliveira V, Laskowicz RD, Boyer L, Lago NR, et al. Intraspecific differences in the immunochemical reactivity and neutralization of venom from Argentinean Bothrops (Rhinocerophis) alternatus by specific experimental antivenoms. Toxicon. 2014;85:31-45. 72. Fernandez S, Hodgson W, Chaisakul J, Kornhauser R, Konstantakopoulos N, Smith AI, et al. In vitro toxic effects of puff adder (Bitis arietans) venom, and their neutralization by antivenom. Toxins. 2014;6(5):1586-97. 73. Castro JM, Oliveira TS, Silveira CR, Caporrino MC, Rodriguez D, Moura-da-Silva AM, et al. A neutralizing recombinant single chain antibody, scFv, against BaP1, A P-I hemorrhagic metalloproteinase from Bothrops asper snake venom. Toxicon. 2014;87:81-91. 74. Cajado Carvalho D, Kuniyoshi AK, Kodama RT, Oliveira AK, Serrano SM, Tambourgi DV, et al. Neuropeptide Y family-degrading metallopeptidases in the Tityus serrulatus venom partially blocked by commercial antivenoms. Toxicol Sci. 2014;142(2):418-26. 75. Ahmad Rusmili MR, Yee TT, Mustafa MR, Othman I, Hodgson WC. In-vitro neurotoxicity of two Malaysian krait species (Bungarus candidus and Bungarus fasciatus) venoms: Neutralization by monovalent and polyvalent antivenoms from Thailand. Toxins. 2014;6(3):1036-48. 76. Richard G, Meyers AJ, McLean MD, Arbabi-Ghahroudi M, MacKenzie R, Hall JC. In Vivo Neutralization of α-Cobratoxin with High-Affinity Llama Single-Domain Antibodies (VHHs) and a VHH-Fc Antibody. PLoS ONE. 2013;8(7). 77. Kornhauser R, Isbister GK, O'Leary MA, Mirtschin P, Dunstan N, Hodgson WC. Cross-Neutralisation of the Neurotoxic Effects of Egyptian Cobra Venom with Commercial Tiger Snake Antivenom. Basic and Clinical Pharmacology and Toxicology. 2013;112(2):138-43. 78. Gay C, Maruñak S, Teibler P, Leiva L, Acosta O. Effect of monospecific antibodies against baltergin in myotoxicity induced by Bothrops alternatus venom from northeast of Argentina. Role of metalloproteinases in muscle damage. Toxicon. 2013;63(1):104-11. 79. De-Simone SG, Napoleão-Pego P, Teixeira-Pinto LA, Santos JD, De-Simone TS, Melgarejo AR, et al. Linear B-cell epitopes in BthTX-1, BthTX-II and BthA-1, phospholipase A₂'s from Bothrops jararacussu snake venom, recognized by therapeutically neutralizing commercial horse antivenom. Toxicon. 2013;72:90-101. 80. Ali SA, Yang DC, Jackson TN, Undheim EA, Koludarov I, Wood K, et al. Venom proteomic characterization and relative antivenom neutralization of two medically important Pakistani elapid snakes (Bungarus sindanus and Naja naja). J Proteomics. 2013;89:15-23. 81. Zayerzadeh E, Koohi MK, Mirakabadi AZ, Fardipoor A, Kassaian SE, Rabbani S, et al. Amelioration of cardio-respiratory perturbations following Mesobuthus eupeus envenomation in anesthetized rabbits with commercial polyvalent F(ab')2 antivenom. Toxicon. 2012;59(2):249-56. 82. Yano MY, Matsubara MH, Sano-Martins IS. Evaluation of the efficacy of treatment using bothropic or bothropic/crotalic antivenin in bothrops jararacussu (viperidae) experimental envenomation. Toxicon. 2012;60(2):134. 83. Stoyanova V, Aleksandrov R, Lukarska M, Duhalov D, Atanasov V, Petrova S. Recognition of vipera ammodytes meridionalis neurotoxin vipoxin and its components using phage-displayed scFv and polyclonal antivenom sera. Toxicon. 2012;60(5):802-9. 84. Rucavado A, Escalante T, Shannon JD, Ayala-Castro CN, Villalta M, Gutiérrez JM, et al. Efficacy of IgG and F(ab')2 antivenoms to neutralize snake venom-induced local tissue damage as assessed by the proteomic analysis of wound exudate. J Proteome Res. 2012;11(1):292-305. 85. Rodríguez JP, Gay CC, Fusco LS, Gauna MC, Acosta OC, Leiva LC. Cross-neutralization of the coagulant activity of Crotalus durissus terrificus venom from the northeast of Argentina by bivalent bothropic antivenom. Journal of Venomous Animals and Toxins Including Tropical Diseases. 2012;18(1):116-23. 86. Kuniyoshi AK, Rocha M, Cajado Carvalho D, Juliano MA, Juliano Neto L, Tambourgi DV, et al. Angiotensin-degrading serine peptidase: a new chymotrypsin-like activity in the venom of Bothrops jararaca partially blocked by the commercial antivenom. Toxicon. 2012;59(1):124-31. 87. Senise LV, Yano MY, Santoro ML, Sano-Martins IS. Evaluation of hemostatic disturbances caused by adult and young Bothrops jararaca snake venoms and neutralization by specific antivenin. Journal of Thrombosis and Haemostasis. 2011;9:389. 88. Morokuma K, Kobori N, Fukuda T, Uchida T, Sakai A, Toriba M, et al. Experimental manufacture of equine antivenom against yamakagashi (Rhabdophis tigrinus). Jpn J Infect Dis. 2011;64(5):397-402. 89. Méndez I, Gutiérrez JM, Angulo Y, Calvete JJ, Lomonte B. Comparative study of the cytolytic activity of snake venoms from African spitting cobras (Naja spp., Elapidae) and its neutralization by a polyspecific antivenom. Toxicon. 2011;58(6-7):558-64. 90. Marcon F, Nicholson GM. Identification of presynaptic neurotoxin complexes in the venoms of three Australian copperheads (Austrelaps spp.) and the efficacy of tiger snake antivenom to prevent or reverse neurotoxicity. Toxicon. 2011;58(5):439-52. 91. Lane J, O'Leary MA, Isbister GK. Coagulant effects of black snake (Pseudechis spp.) venoms and in vitro efficacy of commercial antivenom. Toxicon. 2011;58(3):239-46. 92. Camargo TM, de Roodt AR, da Cruz-Höfling MA, Rodrigues-Simioni L. The neuromuscular activity of Micrurus pyrrhocryptus venom and its neutralization by commercial and specific coral snake antivenoms. J Venom Res. 2011;2:24-31. 93. Rostelato-Ferreira S, Rodrigues-Simioni L, Oshima-Franco Y. Heparin and commercial bothropic antivenom against the paralyzing effect of Bothrops jararacussu snake venom. Journal of Venomous Animals and Toxins Including Tropical Diseases. 2010;16(1):34-45. 94. Ponte CG, Nóbrega EL, Fernandes VC, da Silva WD, Suarez-Kurtz G. Inhibition of the myotoxic activities of three African Bitis venoms (B. rhinoceros, B. arietans and B. nasicornis) by a polyvalent antivenom. Toxicon. 2010;55(2-3):536-40. 95. Isbister GK, O'Leary MA, Hagan J, Nichols K, Jacoby T, Davern K, et al. Cross-neutralisation of Australian brown snake, taipan and death adder venoms by monovalent antibodies. Vaccine. 2010;28(3):798-802. 96. Cook DAN, Owen T, Wagstaff SC, Kinne J, Wernery U, Harrison RA. Analysis of camelid IgG for antivenom development: Serological responses of venom-immunised camels to prepare either monospecific or polyspecific antivenoms for West Africa. Toxicon. 2010;56(3):363-72. 97. Cook DA, Samarasekara CL, Wagstaff SC, Kinne J, Wernery U, Harrison RA. Analysis of camelid IgG for antivenom development: Immunoreactivity and preclinical neutralisation of venom-induced pathology by IgG subclasses, and the effect of heat treatment. Toxicon. 2010;56(4):596-603. 98. Buschek S, Ignjatovic V, Summerhayes R, Lowe R. The effect of different snake venoms and anti-venoms on thrombin clotting time in human plasma. Thrombosis Research. 2010;125(4):e149-e52. 99. Rodriguez JP, De Marzi MC, Marunak S, Teibler P, Acosta O, Malchiodi EL, et al. IgG ANTIBODIES AGAINST PHOSPHOLIPASE A(2) FROM Crotalus durissus terrificus: CROSS-REACTION WITH VENOMS FROM Bothrops SPECIES FROM ARGENTINA. Journal of Venomous Animals and Toxins Including Tropical Diseases. 2009;15(3):460-78. 100. O'Leary MA, Isbister GK. Commercial monovalent antivenoms in Australia are polyvalent. Toxicon. 2009;54(2):192-5. 101. Sangiorgio F, Sakate M, Nogueira RMB, Araujo JP, Chavez-Olortegui C. Kinetics of venom and antivenom serum levels, clinical evaluation and therapeutic effectiveness in dogs inoculated with Crotalus durissus terrificus venom. Journal of Venomous Animals and Toxins Including Tropical Diseases. 2008;14(1):100-12. 102. de Abreu VA, Leite GB, Oliveira CB, Hyslop S, Furtado Mde F, Simioni LR. Neurotoxicity of Micrurus altirostris (Uruguayan coral snake) venom and its neutralization by commercial coral snake antivenom and specific antiserum raised in rabbits. Clin Toxicol (Phila). 2008;46(6):519-27. 103. Beghini DG, Damico DC, da Cruz-Höfling MA, Rodrigues-Simioni L, Delatorre MC, Hyslop S, et al. Ability of rabbit antiserum against crotapotin to neutralize the neurotoxic, myotoxic and phospholipase A2 activities of crotoxin from Crotalus durissus cascavella snake venom. Toxicol In Vitro. 2008;22(1):240-8. 104. Price Iii JA, Sanny CG. CroFab™ total anti-venom activity measured by SE-HPLC, and anti-PLA2 activity assayed in vitro at physiological pH. Toxicon. 2007;49(6):848-54. 105. O'Leary MA, Schneider JJ, Krishnan BP, Lavis C, McKendry A, Ong LK, et al. Cross-neutralisation of Australian brown and tiger snake venoms with commercial antivenoms: Cross-reactivity or antivenom mixtures? Toxicon. 2007;50(2):206-13. 106. Isbister GK, O'Leary MA, Schneider JJ, Brown SG, Currie BJ. Efficacy of antivenom against the procoagulant effect of Australian brown snake (Pseudonaja sp.) venom: in vivo and in vitro studies. Toxicon. 2007;49(1):57-67. 107. da Silva NMV, Arruda EZ, Murakami YLB, Moraes RAM, El-Kik CZ, Tomaz MA, et al. Evaluation of three Brazilian antivenom ability to antagonize myonecrosis and hemorrhage induced by Bothrops snarce venoms in a mouse model. Toxicon. 2007;50(2):196-205. 108. Judge RK, Henry PJ, Mirtschin P, Jelinek G, Wilce JA. Toxins not neutralized by brown snake antivenom. Toxicology and Applied Pharmacology. 2006;213(2):117-25. 109. Harrison RA, Hasson SS, Harmsen M, Laing GD, Conrath K, Theakston RD. Neutralisation of venom-induced haemorrhage by IgG from camels and llamas immunised with viper venom and also by endogenous, non-IgG components in camelid sera. Toxicon. 2006;47(3):364-8. 110. Beghini DG, Da Cruz-Höfling MA, Randazzo-Moura P, Rodrigues-Simioni L, Novello JC, Hyslop S, et al. Cross-neutralization of the neurotoxicity of Crotalus durissus terrificus and Bothrops jararacussu venoms by antisera against crotoxin and phospholipase A2 from Crotalus durissus cascavella venom. Toxicon. 2005;46(6):604-11. 111. Zamuner SR, da Cruz-Hofling MA, Corrado AP, Hyslop S, Rodrigues-Simioni U. Comparison of the neurotoxic and myotoxic effects of Brazilian Bothrops venoms and their neutralization by commercial antivenom. Toxicon. 2004;44(3):259-71. 112. Ramasamy S, Isbister GK, Hodgson WC. The efficacy of two antivenoms against the in vitro myotoxic effects of black snake (Pseudechis) venoms in the chick biventer cervicis nerve-muscle preparation. Toxicon. 2004;44(8):837-45. 113. Chetty N, Du A, Hodgson WC, Winkel K, Fry BG. The in vitro neuromuscular activity of Indo-Pacific sea-snake venoms: Efficacy of two commercially available antivenoms. Toxicon. 2004;44(2):193-200. 114. Beghini DG, Hernandez-Oliveira S, Rodrigues-Simioni L, Novello JC, Hyslop S, Marangoni S. Anti-sera raised in rabbits against crotoxin and phospholipase A 2 from Crotalus durissus cascavella venom neutralize the neurotoxicity of the venom and crotoxin. Toxicon. 2004;44(2):141-8. 115. Tibballs J, Kuruppu S, Hodgson WC, Carroll T, Hawdon G, Sourial M, et al. Cardiovascular, haematological and neurological effects of the venom of the Papua New Guinean small-eyed snake (Micropechis ikaheka) and their neutralisation with CSL polyvalent and black snake antivenoms. Toxicon. 2003;42(6):647-55. 116. Tanjoni I, Butera D, Spencer PJ, Takehara HA, Fernandes I, Moura-da-Silva AM. Phylogenetic conservation of a snake venom metalloproteinase epitope recognized by a monoclonal antibody that neutralizes hemorrhagic activity. Toxicon. 2003;42(7):809-16. 117. Sánchez EE, Ramírez MS, Galán JA, López G, Rodríguez-Acosta A, Pérez JC. Cross reactivity of three antivenoms against North American snake venoms. Toxicon. 2003;41(3):315-20. 118. Harrison RA, Wüster W, Theakston RD. The conserved structure of snake venom toxins confers extensive immunological cross-reactivity to toxin-specific antibody. Toxicon. 2003;41(4):441-9. 119. Battellino C, Piazza R, da Silva AM, Cury Y, Farsky SH. Assessment of efficacy of bothropic antivenom therapy on microcirculatory effects induced by Bothrops jararaca snake venom. Toxicon. 2003;41(5):583-93. 120. Al-Abdulla I, Garnvwa JM, Rawat S, Smith DS, Landon J, Nasidi A. Formulation of a liquid ovine Fab-based antivenom for the treatment of envenomation by the Nigerian carpet viper (Echis ocellatus). Toxicon. 2003;42(4):399-404. 121. Abdel Latif SS, Wanas S, Malak GA, Helmy MH. Efficacy of IgG, Fab, and F(ab')2 fragments of horse antivenom in the treatment of local symptoms after Cerastes cerastes (Egyptian snake) bite. African Journal of Biotechnology. 2003;2(7):197-205. 122. Qian MD, Villeval JL, Xiong X, Jandrot-Perrus M, Nagashima K, Tonra J, et al. Anti GPVI human antibodies neutralizing collagen-induced platelet aggregation isolated from a combinatorial phage display library. Hum Antibodies. 2002;11(3):97-105. 123. Picolo G, Chacur M, Gutiérrez JM, Teixeira CF, Cury Y. Evaluation of antivenoms in the neutralization of hyperalgesia and edema induced by Bothrops jararaca and Bothrops asper snake venoms. Braz J Med Biol Res. 2002;35(10):1221-8. 124. Anai K, Sugiki M, Yoshida E, Maruyama M. Neutralization of a snake venom hemorrhagic metalloproteinase prevents coagulopathy after subcutaneous injection of Bothrops jararaca venom in rats. Toxicon. 2002;40(1):63-8. 125. Oshima-Franco Y, Leite GB, Silva GH, Cardoso DF, Hyslop S, Giglio JR, et al. Neutralization of the pharmacological effects of bothropstoxin-I from Bothrops jararacussu (jararacuçu) venom by crotoxin antiserum and heparin. Toxicon. 2001;39(10):1477-85. 126. Colombini M, Fernandes I, Cardoso DF, Moura-da-Silva AM. Lachesis muta muta venom: Immunological differences compared with Bothrops atrox venom and importance of specific antivenom therapy. Toxicon. 2001;39(5):711-9. 127. Oshima-Franco Y, Hyslop S, Cintra ACO, Giglio JR, Da Cruz-Höfling MA, Rodrigues-Simioni L. Neutralizing capacity of commercial bothropic antivenom against Bothrops jararacussu venom and bothropstoxin-I. Muscle and Nerve. 2000;23(12):1832-9. 128. León G, Valverde JM, Rojas G, Lomonte B, Gutiérrez JM. Comparative study on the ability of IgG and Fab sheep antivenoms to neutralize local hemorrhage, edema and myonecrosis induced by Bothrops asper (terciopelo) snake venom. Toxicon. 2000;38(2):233-44. 129. Harrison RA, Moura-Da-Silva AM, Laing GD, Wu Y, Richards A, Broadhead A, et al. Antibody from mice immunized with DNA encoding the carboxyl-disintegrin and cysteine-rich domain (JD9) of the haemorrhagic metalloprotease, Jararhagin, inhibits the main lethal component of viper venom. Clin Exp Immunol. 2000;121(2):358-63. 130. Fernandes I, Tavares FL, Sano-Martins IS, Takehara HA. Efficacy of bothropic antivenom and its IgG(T) fraction in restoring fibrinogen levels of Bothrops jararaca envenomed mice. Toxicon. 2000;38(7):995-8. 131. Estêvão-Costa MI, Martins MS, Sánchez EF, Diniz CR, Chávez-Olórtegui C. Neutralization of the hemorrhagic activity of Bothrops and Lachesis snake venoms by a monoclonal antibody against mutalysin-II. Toxicon. 2000;38(1):139-44. 132. Crachi MT, Hammer LW, Hodgson WC. The effects of antivenom on the in vitro neurotoxicity of venoms from the taipans Oxyuranus scutellatus, Oxyuranus microlepidotus and Oxyuranus scutellatus canni. Toxicon. 1999;37(12):1771-8. 133. Tokeshi Y, Nakamura M, Kinjoh K, Sunagawa M, Hanashiro K, Kosugi T. Inhibition of habutobin activities by habu antivenom. Toxicon. 1998;36(1):53-62. 134. Pepin-Covatta S, Lutsch C, Lang J, Scherrmann JM. Preclinical assessment of immunoreactivity of a new purified equine F(ab′)2 against European viper venom. Journal of Pharmaceutical Sciences. 1998;87(2):221-5. 135. Masci PP, Mirtschin PJ, Nias TN, Turnbull RK, Kuchel TR, Whitaker AN. Brown snakes (Pseudonaja genus): Venom yields, prothrombin activator neutralization and implications affecting antivenom usage. Anaesthesia and Intensive Care. 1998;26(3):276-81. 136. Gutiérrez JM, León G, Rojas G, Lomonte B, Rucavado A, Chaves F. Neutralization of local tissue damage induced by Bothrops asper (terciopelo) snake venom. Toxicon. 1998;36(11):1529-38. 137. León G, Rojas G, Lomonte B, Gutiérrez JM. Immunoglobulin G and F(ab')2 polyvalent antivenoms do not differ in their ability to neutralize hemorrhage, edema and myonecrosis induced by bothrops asper (terciopelo) snake venom. Toxicon. 1997;35(11):1627-37. 138. Borkow G, Gutiérrez JM, Ovadia M. Inhibition of the hemorrhagic activity of Bothrops asper venom by a novel neutralizing mixture. Toxicon. 1997;35(6):865-77. 139. Borkow G, Gutierrez JM, Ovadia M. Inhibition of toxic activities of Bothrops asper venom and other crotalid snake venoms by a novel neutralizing mixture. Toxicology and Applied Pharmacology. 1997;147(2):442-7. 140. Sprivulis P, Jelinek GA, Marshall L. Efficacy and potency of antivenoms in neutralizing the procoagulant effects of Australian snake venoms in dog and human plasma. Anaesthesia and Intensive Care. 1996;24(3):379-81. 141. Rucavado A, Lomonte B. Neutralization of myonecrosis, hemorrhage, and edema induced by Bothrops asper snake venom by homologous and heterologous pre-existing antibodies in mice. Toxicon. 1996;34(5):567-77. 142. Lomonte B, León G, Hanson LA. Similar effectiveness of Fab and F(ab')2 antivenoms in the neutralization of hemorrhagic activity of vipera Berus snake venom in mice. Toxicon. 1996;34(10):1197-202. 143. Li Q, Ownby CL. Immunological studies of rabbit antibodies against hemorrhagic fractions of Crotalus viridis viridis venom: Role of crossreacting antibodies in neutralization. Comparative Biochemistry and Physiology - A Physiology. 1996;114(2):167-73. 144. Li QZ, Ownby CL. CROSS-REACTIVITIES OF MONOCLONAL-ANTIBODIES AGAINST HEMORRHAGIC TOXINS OF PRAIRIE RATTLESNAKE (CROTALUS-VIRIDIS VIRIDIS) VENOM. Comparative Biochemistry and Physiology B-Biochemistry & Molecular Biology. 1994;107(1):51-9. 145. Domingos MO, Takehara HA, Laing G, Snowden KF, Sells PG, Mota I, et al. DETECTION AND NEUTRALIZATION OF B-JARARACA VENOM IN MICE. Brazilian Journal of Medical and Biological Research. 1994;27(11):2613-22. 146. Alape-Girón A, Gustafsson B, Lomonte B, Thelestam M, Gutiérrez JM. Immunochemical characterization of Micrurus nigrocinctus nigrocinctus venom with monoclonal and polyclonal antibodies. Toxicon. 1994;32(6):695-712. 147. Ferreira ML, Moura-Da-Silva AM, Mota I. Neutralization of different activities of venoms from nine species of Bothrops snakes by Bothrops jararaca antivenom. Toxicon. 1992;30(12):1591-602. 148. Choumet V, Faure G, Robbe-Vincent A, Saliou B, Mazié JC, Bon C. Immunochemical analysis of a snake venom phospholipase A2 neurotoxin, crotoxin, with monoclonal antibodies. Mol Immunol. 1992;29(7-8):871-82. 149. Tibballs J, Sutherland S. The efficacy of antivenom in prevension of cardiovascular depression and coagulopathy induced by Brown snake (Pseudonaja) species venom. Anaesthesia and Intensive Care. 1991;19(4):530-4. 150. Moura-da-Silva AM, Cardoso DF, Tanizaki MM, Mota I. Neutralization of myotoxic activity of Bothrops venoms by antisera to purified myotoxins and to crude venoms. Toxicon. 1991;29(12):1471-80. 151. Bailey GS, Al-Joufi A, Rawat S, Smith DC. Neutralization of kinin-releasing enzymes of crotalid venoms by monospecific and polyspecific antivenoms. Toxicon. 1991;29(6):777-81. 152. Al-Joufi A, Bailey GS, Reddi K, Smith DC. Neutralization of kinin-releasing enzymes from viperid venoms by antivenom IgG fragments. Toxicon. 1991;29(12):1509-11. 153. Tan NH, Saifuddin MN, Jaafar MI. Preparation of antibodies to king cobra (Ophiophagus hannah) venom hemorrhagin and investigation of their cross-reactivity. Toxicon. 1990;28(11):1355-9. 154. Lomonte B, Gutierrez JM, Carmona E, Rovira ME. Equine antibodies to Bothrops asper myotoxin II: Isolation from polyvalent antivenom and neutralizing ability. Toxicon. 1990;28(4):379-84. 155. da Silva AM, Lima MR, Nishikawa AK, Brodskyn CI, dos Santos MC, Furtado MF, et al. Antigenic cross-reactivity of venoms obtained from snakes of genus Bothrops. Toxicon. 1990;28(2):181-8. 156. Gené JA, Roy A, Rojas G, Gutiérrez JM, Cerdas L. Comparative study on coagulant, defibrinating, fibrinolytic and fibrinogenolytic activities of Costa Rican crotaline snake venoms and their neutralization by a polyvalent antivenom. Toxicon. 1989;27(8):841-8. 157. Claus I, Mebs D. Cross-neutralization of thrombin-like enzymes in snake venoms by polyvalent antivenoms. Toxicon. 1989;27(12):1397-9. 158. Mebs D, Pohlmann S, Von Tenspolde W. Snake venom hemorrhagins: Neutralization by commercial antivenoms. Toxicon. 1988;26(5):453-8. 159. Lomonte B, Gutiérrez J, Moreno E, Cerdas L. Antibody neutralization of a myotoxin from the venom of Bothrops asper (terciopelo). Toxicon. 1987;25(4):443-9. 160. Ownby CL, Colberg TR. Ability of polyvalent (Crotalidae) antivenom to neutralize local myonecrosis induced by Crotalus atrox venom. Toxicon. 1986;24(2):201-3. 161. Mebs D. Myotoxic activity of phospholipases A2 isolated from cobra venoms: Neutralization by polyvalent antivenoms. Toxicon. 1986;24(10):1001-8. 162. Gutiérrez JM, Rojas G, Lomonte B, Gené JA, Cerdas L. Comparative study of the edema-forming activity of Costa Rican snake venoms and its neutralization by a polyvalent antivenom. Comp Biochem Physiol C Comp Pharmacol Toxicol. 1986;85(1):171-5. 163. Lomonte B. Edema-forming activity of bushmaster (Lachesis muta stenophrys) and Central American Rattlesnake (Crotalus durissus durissus) venoms and neutralization by a polyvalent antivenom. Toxicon. 1985;23(1):173-6. 164. Gutierrez JM, Gene JA, Rojas G, Cerdas L. Neutralization of proteolytic and hemorrhagic activities of Costa Rican snake venoms by a polyvalent antivenom. Toxicon. 1985;23(6):887-93. 165. Gene JA, Gomez M, Gutierrez JM, Cerdas L. Neutralization of hyaluronidase and indirect hemolytic activities of Costa Rican snake venoms by a polyvalent antivenom. Toxicon. 1985;23(6):1015-8. 166. Garfin SR, Castilonia RR, Mubarak SJ, Hargens AR, Akeson WH, Russell FE. The effect of antivenin on intramuscular pressure elevations induced by rattlesnake venom. Toxicon. 1985;23(4):677-80. 167. Ownby CL, Colberg TR, Claypool PL, Odell GV. In vivo test of the ability of antiserum to myotoxin a from prairie rattlesnake (Crotalus viridis viridis) venom to neutralize local myonecrosis induced by myotoxin a and homologous crude venom. Toxicon. 1984;22(1):99-105. 168. Bar-Or D, Sullivan JB, Jr., Black E, Russell FE. Neutralization of croatalidae venom induced platelet aggregation by affinity chromatography isolated IgG to Crotalus viridis helleri venom. J Toxicol Clin Toxicol. 1984;22(1):1-9. 169. Ownby CL, Odell GV, Woods WM, Colberg TR. Ability of antiserum to myotoxin alpha from prairie rattlesnake (Crotalus viridis viridis) venom to neutralize local myotoxicity and lethal effects of myotoxin alpha and homologous crude venom. Toxicon. 1983;21(1):35-45. 170. Russell FE, Ruzić N, Gonzalez H. Effectiveness of antivenin (Crotalidae) polyvalent following injection of Crotalus venom. Toxicon. 1973;11(6):461-4. 171. Homma M, Tu AT. Antivenin for the treatment of local tissue damage due to envenomation by Southeast Asian snakes. Ineffectiveness in the prevention of local tissue damage in mice after envenomation. Am J Trop Med Hyg. 1970;19(5):880-4. 172. Rosenfeld G, Kelen EM. Cross neutralization of the coagulant activity of some snake venoms by antivenins. Toxicon. 1966;4(1):7-15. 173. Luzzio AJ, Trevino GS, De Venuto F. Precipitin and Neutralizing Antibody Response Elicited by Crotalus atrox Venom-Antivenom Precipitate. Proceedings of the Society for Experimental Biology and Medicine. 1966;122(1):295-9. 174. Chain E. NEUTRALISATION OF THE ANTIFERMENTING PRINCIPLE IN SNAKE VENOMS BY ANTIVENINS. Quarterly Journal of Experimental Physiology. 1937;27(1):49-54. 175. Semple D, Lamb G. The Neutralising Power of Calmette's Antivenomous Serum: Its Value in the Treatment of Snakebite. Br Med J. 1899;1(1996):781-4. 176. Antunes E, Rodrigues-Simioni L, Prado-Franceschi J. Cross-neutralization on the histamine-releasing activity of snake venoms. Acta Physiol Pharmacol Latinoam. 1989;39(4):431-8. 177. Gutierrez JM, Chaves F, Bolanos R. Neutralization of local effects of Bothrops asper venom by a polyvalent antivenom. Toxicon. 1981;19(4):493-500. |
| Non-Asian snakes (n = 105) | 1. Alfaro-Chinchilla A, Segura A, Gomez A, Diaz C, Corrales G, Chacon D, et al. Expanding the neutralization scope of the Central American antivenom (PoliVal-ICP) to include the venom of Crotalus durissus pifanorum. J Proteomics. 2021;246:12. 2. Alomran N, Alsolaiss J, Albulescu LO, Crittenden E, Harrison RA, Ainsworth S, et al. Pathology-specific experimental antivenoms for haemotoxic snakebite: The impact of immunogen diversity on the in vitro cross-reactivity and in vivo neutralisation of geographically diverse snake venoms. Plos Neglect Trop Dis. 2021;15(8):26. 3. Franco-Servin C, Neri-Castro E, Benard-Valle M, Alagon A, Rosales-Garcia RA, Guerrero-Alba R, et al. Biological and Biochemical Characterization of Coronado Island Rattlesnake (Crotalus helleri caliginis) Venom and Antivenom Neutralization. Toxins. 2021;13(8):21. 4. Ruiz-Campos M, Sanz L, Bonilla F, Sasa M, Lomonte B, Zaruma-Torres F, et al. Venomics of the poorly studied hognosed pitvipers Porthidium arcosae and Porthidium volcanicum. J Proteomics. 2021;249:14. 5. Acosta-Pena A, Nunez V, Pereanez JA, Rey-Suarez P. Immunorecognition and Neutralization of Crotalus durissus cumanensis Venom by a Commercial Antivenom Produced in Colombia. Toxins. 2022;14(4):14. 6. Manson EZ, Kyama MC, Gikunju JK, Kimani J, Kimotho JH. Evaluation of lethality and cytotoxic effects induced by Naja ashei (large brown spitting cobra) venom and the envenomation-neutralizing efficacy of selected commercial antivenoms in Kenya. Toxicon: X. 2022;14. 7. Okumu MO, Mbaria JM, Gikunju JK, Mbuthia PG, Madadi VO, Ochola FO, et al. Preclinical efficacy testing of three antivenoms against Naja ashei venom-induced lethality. Toxicon: X. 2022;14. 8. Mena G, Chaves-Araya S, Chacón J, Török E, Török F, Bonilla F, et al. Proteomic and toxicological analysis of the venom of Micrurus yatesi and its neutralization by an antivenom. Toxicon: X. 2022;13. 9. Ponce-López R, Neri-Castro E, Olvera-Rodríguez F, Sánchez EE, Alagón A, Olvera-Rodríguez A. Neutralization of crotamine by polyclonal antibodies generated against two whole rattlesnake venoms and a novel recombinant fusion protein. Toxicon. 2021;197:70-8. 10. de Roodt AR, Lanari LC, Ramírez JE, Gómez C, Barragán J, Litwin S, et al. Cross-reactivity of some Micrurus venoms against experimental and therapeutic anti-Micrurus antivenoms. Toxicon. 2021;200:153-64. 11. Sánchez A, Segura Á, Pla D, Munuera J, Villalta M, Quesada-Bernat S, et al. Comparative venomics and preclinical efficacy evaluation of a monospecific Hemachatus antivenom towards sub-Saharan Africa cobra venoms. J Proteomics. 2021;240. 12. Wong KY, Tan KY, Tan NH, Tan CH. A Neurotoxic Snake Venom without Phospholipase A2: Proteomics and Cross-Neutralization of the Venom from Senegalese Cobra, Naja senegalensis (Subgenus: Uraeus). Toxins. 2021;13(1). 13. Tan KY, Wong KY, Tan NH, Tan CH. Quantitative proteomics of Naja annulifera (sub-Saharan snouted cobra) venom and neutralization activities of two antivenoms in Africa. International Journal of Biological Macromolecules. 2020;158:605-16. 14. Martínez M, Almaguer J, Saldivar A, Soria R, Mathe H. Preclinical evaluation of the polyspecific antivenom Inoserp™ PAN-AFRICA against the venoms of elapids and viperids of Sub-Saharan Africa region: Neutralization of toxic activities. Toxicon. 2020;177:S52-S3. 15. Harrison RA, Oluoch GO, Ainsworth S, Alsolaiss J, Bolton F, Arias AS, et al. Preclinical antivenom-efficacy testing reveals potentially disturbing deficiencies of snakebite treatment capability in East Africa. PLoS Neglected Tropical Diseases. 2017;11(10). 16. Sánchez A, Segura Á, Vargas M, Herrera M, Villalta M, Estrada R, et al. Expanding the neutralization scope of the EchiTAb-plus-ICP antivenom to include venoms of elapids from Southern Africa. Toxicon. 2017;125:59-64. 17. Calvete JJ, Arias AS, Rodríguez Y, Quesada-Bernat S, Sánchez LV, Chippaux JP, et al. Preclinical evaluation of three polyspecific antivenoms against the venom of Echis ocellatus: Neutralization of toxic activities and antivenomics. Toxicon. 2016;119:280-8. 18. Lauridsen LP, Laustsen AH, Lomonte B, Gutiérrez JM. Toxicovenomics and antivenom profiling of the Eastern green mamba snake (Dendroaspis angusticeps). Journal of Proteomics. 2016;136:248-61. 19. Sánchez A, Coto J, Segura Á, Vargas M, Solano G, Herrera M, et al. Effect of geographical variation of Echis ocellatus, Naja nigricollis and Bitis arietans venoms on their neutralization by homologous and heterologous antivenoms. Toxicon. 2015;108:80-3. 20. Sánchez LV, Pla D, Herrera M, Chippaux JP, Calvete JJ, Gutiérrez JM. Evaluation of the preclinical efficacy of four antivenoms, distributed in sub-Saharan Africa, to neutralize the venom of the carpet viper, Echis ocellatus, from Mali, Cameroon, and Nigeria. Toxicon. 2015;106:97-107. 21. Laustsen AH, Lomonte B, Lohse B, Fernández J, Gutiérrez JM. Unveiling the nature of black mamba (Dendroaspis polylepis) venom through venomics and antivenom immunoprofiling: Identification of key toxin targets for antivenom development. Journal of Proteomics. 2015;119:126-42. 22. Sánchez EE, Hotle D, Rodríguez-Acosta A. Neutralization of Bitis parviocula (Ethiopian mountain adder) venom by the South African Institute of Medical Research (SAIMR) antivenom. Revista do Instituto de Medicina Tropical de Sao Paulo. 2011;53(4):213-7. 23. Weinstein SA, Schmidt JJ, Smith LA. Lethal toxins and cross-neutralization of venoms from the African water cobras, Boulengerina annulata annulata and Boulengerina christyi. Toxicon. 1991;29(11):1315-27. 24. Cook DA, Owen T, Wagstaff SC, Kinne J, Wernery U, Harrison RA. Analysis of camelid antibodies for antivenom development: Neutralisation of venom-induced pathology. Toxicon. 2010;56(3):373-80. 25. Petras D, Sanz L, Segura A, Herrera M, Villalta M, Solano D, et al. Snake venomics of African spitting cobras: toxin composition and assessment of congeneric cross-reactivity of the pan-African EchiTAb-Plus-ICP antivenom by antivenomics and neutralization approaches. J Proteome Res. 2011;10(3):1266-80. 26. Casewell NR, Cook DA, Wagstaff SC, Nasidi A, Durfa N, Wüster W, et al. Pre-clinical assays predict pan-African Echis viper efficacy for a species-specific antivenom. PLoS Negl Trop Dis. 2010;4(10):e851. 27. Ramos-Cerrillo B, de Roodt AR, Chippaux JP, Olguín L, Casasola A, Guzmán G, et al. Characterization of a new polyvalent antivenom (Antivipmyn Africa) against African vipers and elapids. Toxicon. 2008;52(8):881-8. 28. Abubakar SB, Abubakar IS, Habib AG, Nasidi A, Durfa N, Yusuf PO, et al. Pre-clinical and preliminary dose-finding and safety studies to identify candidate antivenoms for treatment of envenoming by saw-scaled or carpet vipers (Echis ocellatus) in northern Nigeria. Toxicon. 2010;55(4):719-23. 29. Segura A, Villalta M, Herrera M, León G, Harrison R, Durfa N, et al. Preclinical assessment of the efficacy of a new antivenom (EchiTAb-Plus-ICP) for the treatment of viper envenoming in sub-Saharan Africa. Toxicon. 2010;55(2-3):369-74. 30. Sánchez A, Segura Á, Pla D, Munuera J, Villalta M, Quesada-Bernat S, et al. Comparative venomics and preclinical efficacy evaluation of a monospecific Hemachatus antivenom towards sub-Saharan Africa cobra venoms. J Proteomics. 2021;240:104196. 31. Ochola FO, Okumu MO, Gikunju JK, Mbaria JM, Muchemi GM, Nderitu JG. Neutralization of the lethality of the venom of Dendroaspis polylepis (black mamba) in mice by two polyvalent antivenoms used in Kenyan hospitals: Results of a 2009–2011 study. Scientific African. 2019;5. 32. Whiteley G, Casewell NR, Pla D, Quesada-Bernat S, Logan RAE, Bolton FMS, et al. Defining the pathogenic threat of envenoming by South African shield-nosed and coral snakes (genus Aspidelaps), and revealing the likely efficacy of available antivenom. Journal of Proteomics. 2019;198:186-98. 33. Rey-Suárez P, Lomonte B. Immunological cross-recognition and neutralization studies of Micrurus mipartitus and Micrurus dumerilii venoms by two therapeutic equine antivenoms. Biologicals. 2020;68:40-5. 34. Sánchez M, Solano G, Vargas M, Reta-Mares F, Neri-Castro É, Alagón A, et al. Toxicological profile of medically relevant Crotalus species from Mexico and their neutralization by a Crotalus basiliscus/Bothrops asper antivenom. Toxicon. 2020;179:92-100. 35. Resiere D, Arias AS, Villalta M, Rucavado A, Brouste Y, Cabié A, et al. Preclinical evaluation of the neutralizing ability of a monospecific antivenom for the treatment of envenomings by Bothrops lanceolatus in Martinique. Toxicon. 2018;148:50-5. 36. Solano G, Gómez A, Corrales G, Chacón D, Estrada R, León G. Contributions of the snake venoms of Bothrops asper, Crotalus simus and Lachesis stenophrys to the paraspecificity of the Central American polyspecific antivenom (PoliVal-ICP). Toxicon. 2018;144:1-6. 37. Baudou FG, Litwin S, Lanari LC, Laskowicz RD, Damin CF, Chippaux JP, et al. Antivenom against Crotalus durissus terrificus venom: Immunochemical reactivity and experimental neutralizing capacity. Toxicon. 2017;140:11-7. 38. Madrigal M, Pla D, Sanz L, Barboza E, Arroyo-Portilla C, Corrêa-Netto C, et al. Cross-reactivity, antivenomics, and neutralization of toxic activities of Lachesis venoms by polyspecific and monospecific antivenoms. PLoS Neglected Tropical Diseases. 2017;11(8). 39. Estevao-Costa MI, Gontijo SS, Correia BL, Yarleque A, Vivas-Ruiz D, Rodrigues E, et al. Neutralization of toxicological activities of medically-relevant Bothrops snake venoms and relevant toxins by two polyvalent bothropic antivenoms produced in Peru and Brazil. Toxicon. 2016;122:67-77. 40. Lomonte B, Sasa M, Rey-Suárez P, Bryan W, Gutiérrez JM. Venom of the coral snake Micrurus clarki: Proteomic profile, toxicity, immunological cross-neutralization, and characterization of a three-finger Toxin. Toxins. 2016;8(5). 41. Rey-Suárez P, Núñez V, Fernández J, Lomonte B. Integrative characterization of the venom of the coral snake Micrurus dumerilii (Elapidae) from Colombia: Proteome, toxicity, and cross-neutralization by antivenom. Journal of Proteomics. 2016;136:262-73. 42. Chacón F, Oviedo A, Escalante T, Solano G, Rucavado A, Gutiérrez JM. The lethality test used for estimating the potency of antivenoms against Bothrops asper snake venom: Pathophysiological mechanisms, prophylactic analgesia, and a surrogate in vitro assay. Toxicon. 2015;93:41-50. 43. Laines J, Segura Á, Villalta M, Herrera M, Vargas M, Alvarez G, et al. Toxicity of Bothrops sp snake venoms from Ecuador and preclinical assessment of the neutralizing efficacy of a polyspecific antivenom from Costa Rica. Toxicon. 2014;88:34-7. 44. Lomonte B, Pla D, Sasa M, Tsai WC, Solórzano A, Ureña-Díaz JM, et al. Two color morphs of the pelagic yellow-bellied sea snake, Pelamis platura, from different locations of Costa Rica: Snake venomics, toxicity, and neutralization by antivenom. Journal of Proteomics. 2014;103:137-52. 45. De Roodt AR, Clement H, Dolab JA, Litwin S, Hajos SE, Boyer L, et al. Protein content of antivenoms and relationship with their immunochemical reactivity and neutralization assays. Clinical Toxicology. 2014;52(6):594-603. 46. Gutiérrez JM, Tsai WC, Pla D, Solano G, Lomonte B, Sanz L, et al. Preclinical assessment of a polyspecific antivenom against the venoms of Cerrophidion sasai, Porthidium nasutum and Porthidium ophryomegas: Insights from combined antivenomics and neutralization assays. Toxicon. 2013;64:60-9. 47. Segura Á, Herrera M, Villalta M, Vargas M, Uscanga-Reynell A, de León-Rosales SP, et al. Venom of Bothrops asper from Mexico and Costa Rica: Intraspecific variation and cross-neutralization by antivenoms. Toxicon. 2012;59(1):158-62. 48. Gutiérrez JM, Sanz L, Escolano J, Fernández J, Lomonte B, Angulo Y, et al. Snake venomics of the lesser antillean pit vipers bothrops caribbaeus and Bothrops lanceolatus: Correlation with toxicological activities and immunoreactivity of a heterologous antivenom. Journal of Proteome Research. 2008;7(10):4396-408. 49. Lira MS, Furtado MF, Martins LMP, Lopes-Ferreira M, Santoro ML, Barbaro KC. Enzymatic and immunochemical characterization of Bothrops insularis venom and its neutralization by polyspecific Bothrops antivenom. Toxicon. 2007;49(7):982-94. 50. Rojas E, Quesada L, Arce V, Lomonte B, Rojas G, Gutiérrez JM. Neutralization of four Peruvian Bothrops sp. snake venoms by polyvalent antivenoms produced in Perú and Costa Rica: Preclinical assessment. Acta Tropica. 2005;93(1):85-95. 51. De Roodt AR, Paniagua-Solis JF, Dolab JA, Estévez-Ramiréz J, Ramos-Cerrillo B, Litwin S, et al. Effectiveness of two common antivenoms for North, Central, and South American Micrurus envenomations. Journal of Toxicology - Clinical Toxicology. 2004;42(2):171-8. 52. Galán JA, Sánchez EE, Rodríguez-Acosta A, Pérez JC. Neutralization of venoms from two Southern Pacific Rattlesnakes (Crotalus helleri) with commercial antivenoms and endothermic animal sera. Toxicon. 2004;43(7):791-9. 53. Arce V, Rojas E, Ownby CL, Rojas G, Gutiérrez JM. Preclinical assessment of the ability of polyvalent (Crotalinae) and anticoral (Elapidae) antivenoms produced in Costa Rica to neutralize the venoms of North American snakes. Toxicon. 2003;41(7):851-60. 54. Sánchez EE, Galán JA, Perez JC, Rodríguez-Acosta A, Chase PB, Pérez JC. The efficacy of two antivenoms against the venom of North American snakes. Toxicon. 2003;41(3):357-65. 55. Moraes FV, Sousa-e-Silva MCC, Barbaro KC, Leitão MA, Furtado MFD. Biological and immunochemical characterization of Micrurus altirostris venom and serum neutralization of its toxic activities. Toxicon. 2003;41(1):71-9. 56. Saravia P, Rojas E, Escalante T, Arce V, Chaves E, Velásquez R, et al. The venom of Bothrops asper from Guatemala: Toxic activities and neutralization by antivenoms. Toxicon. 2001;39(2-3):401-5. 57. Rojas E, Saravia P, Angulo Y, Arce V, Lomonte B, Chávez JJ, et al. Venom of the crotaline snake Atropoides nummifer (jumping viper) from Guatemala and Honduras: Comparative toxicological characterization, isolation of a myotoxic phospholipase A2 homologue and neutralization by two antivenoms. Comparative Biochemistry and Physiology - C Toxicology and Pharmacology. 2001;129(2):151-62. 58. Muniz EG, Maria WS, Estevão-Costa MI, Buhrnheim P, Chávez-Olórtegui C. Neutralizing potency of horse antibothropic Brazilian antivenom against Bothrops snake venoms from the Amazonian rain forest. Toxicon. 2000;38(12):1859-63. 59. Fernandes I, Lima EX, Takehara HA, Moura-Da-Silva AM, Tanjoni I, Gutiérrez JM. Horse IgG isotypes and cross-neutralization of two snake antivenoms produced in Brazil and Costa Rica. Toxicon. 2000;38(5):633-44. 60. Bogarín G, Romero M, Rojas G, Lutsch C, Casadamont M, Lang J, et al. Neutralization, by a monospecific Bothrops lanceolatus antivenom, of toxic activities induced by homologous and heterologous Bothirops snake venoms. Toxicon. 1999;37(3):551-7. 61. Otero R, Núñez V, Gutiérrez JM, Robles A, Estrada R, Osorio RG, et al. Neutralizing capacity of a new monovalent anti-Bothrops atrox antivenom: Comparison with two commercial antivenoms. Brazilian Journal of Medical and Biological Research. 1997;30(3):375-9. 62. Otero R, Nunez V, Osorio RG, Gutierrez JM, Giraldo CA, Posada LE. Ability of six Latin American antivenoms to neutralize the venom of mapana equis (Bothrops atrox) from Antioquia and Choco (Colombia) Choco. Toxicon. 1995;33(6):809-15. 63. Gutierrez JM, Rojas G, Perez A, Arguello I, Lomonte B. Neutralization of coral snake Micrurus nigrocinctus venom by a monovalent antivenom. Brazilian Journal of Medical and Biological Research. 1991;24(7):701-10. 64. Ramos HR, Vassão RC, de Roodt AR, Santos ESEC, Mirtschin P, Ho PL, et al. Cross neutralization of coral snake venoms by commercial Australian snake antivenoms. Clin Toxicol (Phila). 2017;55(1):33-9. 65. Sousa LF, Nicolau CA, Peixoto PS, Bernardoni JL, Oliveira SS, Portes-Junior JA, et al. Comparison of phylogeny, venom composition and neutralization by antivenom in diverse species of bothrops complex. PLoS Negl Trop Dis. 2013;7(9):e2442. 66. Tanaka GD, Furtado Mde F, Portaro FC, Sant'Anna OA, Tambourgi DV. Diversity of Micrurus snake species related to their venom toxic effects and the prospective of antivenom neutralization. PLoS Negl Trop Dis. 2010;4(3):e622. 67. de Roodt AR, Lanari LC, de Oliveira VC, Laskowicz RD, Stock RP. Neutralization of Bothrops alternatus regional venom pools and individual venoms by antivenom: a systematic comparison. Toxicon. 2011;57(7-8):1073-80. 68. Segura Á, Herrera M, Vargas M, Villalta M, Uscanga-Reynell A, León G, et al. Preclinical efficacy against toxic activities of medically relevant Bothrops sp. (Serpentes: Viperidae) snake venoms by a polyspecific antivenom produced in Mexico. Rev Biol Trop. 2017;65(1):345-50. 69. Theakston RD, Laing GD, Fielding CM, Lascano AF, Touzet JM, Vallejo F, et al. Treatment of snake bites by Bothrops species and Lachesis muta in Ecuador: laboratory screening of candidate antivenoms. Trans R Soc Trop Med Hyg. 1995;89(5):550-4. 70. de Roodt AR, Dolab JA, Fernández T, Segre L, Hajos SE. Cross-reactivity and heterologous neutralization of crotaline antivenoms used in Argentina. Toxicon. 1998;36(7):1025-38. 71. Carroll SB, Thalley BS, Theakston RD, Laing G. Comparison of the purity and efficacy of affinity purified avian antivenoms with commercial equine crotalid antivenoms. Toxicon. 1992;30(9):1017-25. 72. Bogarín G, Morais JF, Yamaguchi IK, Stephano MA, Marcelino JR, Nishikawa AK, et al. Neutralization of crotaline snake venoms from Central and South America by antivenoms produced in Brazil and Costa Rica. Toxicon. 2000;38(10):1429-41. 73. Laing GD, Yarleque A, Marcelo A, Rodriguez E, Warrell DA, Theakston RD. Preclinical testing of three South American antivenoms against the venoms of five medically-important Peruvian snake venoms. Toxicon. 2004;44(1):103-6. 74. Baxter EH, Gallichio HA. Protection against sea snake envenomation: comparative potency of four antivenenes. Toxicon. 1976;14(5):347-55. 75. Segura A, Castillo MC, Núñez V, Yarlequé A, Gonçalves LR, Villalta M, et al. Preclinical assessment of the neutralizing capacity of antivenoms produced in six Latin American countries against medically-relevant Bothrops snake venoms. Toxicon. 2010;56(6):980-9. 76. Mora-Obando D, Pla D, Lomonte B, Guerrero-Vargas JA, Ayerbe S, Calvete JJ. Antivenomics and in vivo preclinical efficacy of six Latin American antivenoms towards south-western Colombian Bothrops asper lineage venoms. PLoS Negl Trop Dis. 2021;15(2):e0009073. 77. Furtado Mde F, Cardoso ST, Soares OE, Pereira AP, Fernandes DS, Tambourgi DV, et al. Antigenic cross-reactivity and immunogenicity of Bothrops venoms from snakes of the Amazon region. Toxicon. 2010;55(4):881-7. 78. Laing GD, Theakston RDG, Leite RP, Dias da Silva WD, Warrell DA, Biasg. Comparison of the potency of three Brazilian Bothrops antivenoms using in vivo rodent and in vitro assays. Toxicon. 1992;30(10):1219-25. 79. Gutiérrez J, Rojas G, Cerdas L. Ability of a polyvalent antivenom to neutralize the venom of Lachesis Muta Melanocephala, a new Costa Rican subspecies of the bushmaster. Toxicon. 1987;25(7):713-20. 80. Velez SM, Salazar M, de Patino HA, Gomez L, Rodriguez A, Correa D, et al. Geographical variability of the venoms of four populations of Bothrops asper from Panama: Toxicological analysis and neutralization by a polyvalent antivenom. Toxicon. 2017;132:55-61. 81. Tanaka GD, Sant'Anna OA, Marcelino JR, da Luz ACL, da Rocha MMT, Tambourgi DV. Micrurus snake species: Venom immunogenicity, antiserum cross-reactivity and neutralization potential. Toxicon. 2016;117:59-68. 82. Schneider FS, Starling MC, Duarte CG, de Avila RM, Kalapothakis E, Suarez WS, et al. Preclinical testing of Peruvian anti-bothropic anti-venom against Bothrops andianus snake venom. Toxicon. 2012;60(6):1018-21. 83. Sanchez EE, Lopez-Johnston JC, Rodriguez-Acosta A, Pereza JC. Neutralization of two North American coral snake venoms with United States and Mexican antivenoms. Toxicon. 2008;51(2):297-303. 84. Rocha MMT, Paixao-Cavalcante D, Tambourgi DV, Furtado MDD. Duvernoy's gland secretion of Philodryas olfersii and Philodryas patagoniensis (Colubridae): Neutralization of local and systemic effects by commercial bothropic antivenom (Bothrops genus). Toxicon. 2006;47(1):95-103. 85. Pla D, Bande BW, Welton RE, Paiva OK, Sanz L, Segura Á, et al. Proteomics and antivenomics of Papuan black snake (Pseudechis papuanus) venom with analysis of its toxicological profile and the preclinical efficacy of Australian antivenoms. Journal of Proteomics. 2017;150:201-15. 86. Laustsen AH, Gutiérrez JM, Rasmussen AR, Engmark M, Gravlund P, Sanders KL, et al. Danger in the reef: Proteome, toxicity, and neutralization of the venom of the olive sea snake, Aipysurus laevis. Toxicon. 2015;107:187-96. 87. Pla D, Paiva OK, Sanz L, Beutler M, Wright CE, Calvete JJ, et al. Preclinical efficacy of Australian antivenoms against the venom of the small-eyed snake, Micropechis ikaheka, from Papua New Guinea: An antivenomics and neutralization study. Journal of Proteomics. 2014;110:198-208. 88. Vargas M, Segura A, Herrera M, Villalta M, Estrada R, Cerdas M, et al. Preclinical evaluation of caprylic acid-fractionated igg antivenom for the treatment of taipan (Oxyuranus scutellatus) envenoming in Papua New Guinea. PLoS Neglected Tropical Diseases. 2011;5(5). 89. Pla D, Quesada-Bernat S, Rodríguez Y, Sánchez A, Vargas M, Villalta M, et al. Dagestan blunt-nosed viper, Macrovipera lebetina obtusa (Dwigubsky, 1832), venom. Venomics, antivenomics, and neutralization assays of the lethal and toxic venom activities by anti-Macrovipera lebetina turanica and anti-Vipera berus berus antivenoms. Toxicon: X. 2020;6. 90. Resiere D, Villalta M, Arias AS, Kallel H, Nèviére R, Vidal N, et al. Snakebite envenoming in French Guiana: Assessment of the preclinical efficacy against the venom of Bothrops atrox of two polyspecific antivenoms. Toxicon. 2020;173:1-4. 91. García-Arredondo A, Martínez M, Calderón A, Saldívar A, Soria R. Preclinical assessment of a new polyvalent antivenom (Inoserp europe) against several species of the subfamily viperinae. Toxins. 2019;11(3). 92. Al-Shekhadat RI, Lopushanskaya KS, Segura Á, Gutiérrez JM, Calvete JJ, Pla D. Vipera berus berus venom from Russia: Venomics, bioactivities and preclinical assessment of microgen antivenom. Toxins. 2019;11(2). 93. Casewell NR, Al-Abdulla I, Smith D, Coxon R, Landon J. Immunological cross-reactivity and neutralisation of European viper venoms with the monospecific Vipera berus antivenom ViperaTAb. Toxins (Basel). 2014;6(8):2471-82. 94. Kurtovic T, Balija ML, Ayvazyan N, Halassy B. Paraspecificity of Vipera a. ammodytes-specific antivenom towards Montivipera raddei and Macrovipera lebetina obtusa venoms. Toxicon. 2014;78:103-12. 95. Kurtovic T, Balija ML, Brvar M, Borak MD, Lukacevic SM, Halassy B. Comparison of Preclinical Properties of Several Available Antivenoms in the Search for Effective Treatment of Vipera ammodytes and Vipera berus Envenoming. Toxins. 2021;13(3). 96. Bogarin G, Segura E, Duran G, Lomonte B, Roja G, Gutierrez JM. Evaluation of the neutralizing ability of four commercially available antivenoms against the venom of Bothrops asper from Costa Rica. Toxicon. 1995;33(9):1242-7. 97. Bolaños R, Muñoz G, Cerdas L. Toxicidad, neutralizacion e inmunoelectroforesis de los venenos de Lachesis muta de Costa Rica y Colombia. Toxicon. 1978;16(3):295-300. 98. De Roodt AR, Dolab JA, Hajos SE, Fernandez T, Segre L. Neutralizing ability of antiophidic sera against the venom of Bothrops moojeni (lance-headed viper) venom. Medicina. 1997;57(6):667-76. 99. De Roodt AR, Vidal JC, Litwin S, Dokmetjian JC, Dolab JA, Hajos SE, et al. Cross neutralization of Bothrops jararacussu venom by heterologous antivenoms. Medicina. 1999;59(3):238-42. 100. Dos Santos MC, Ferreira LC, Da Silva WD, Furtado MF. [Characterization of the biological activities of the 'yellow' and 'white' venoms from Crotalus durissus ruruima compared with the Crotalus durissus terrificus venom. Neutralizing activity of Crotalus durissus ruruima antivenins]. Toxicon. 1993;31(11):1459-69. 101. dos-Santos MC, Gonçalves LR, Fortes-Dias CL, Cury Y, Gutiérrez JM, Furtado Mde F. [The efficacy of the bothropic-crotalic antivenom in the neutralization of the main Bothrops jararacussu venom effects]. Rev Inst Med Trop Sao Paulo. 1992;34(2):77-83. 102. Duque-Zerpa CT, Fernandez I, Vargas A, Lopez-Johnsthon JC, Scannone-Tempone H. Toxinological Characterization of the Bothrops atrox Venom from Puerto Ayacucho, Amazonas State (Venezuela) and its Neutralization Capability by Venezuelan Antivenom. Revista Cientifica-Facultad De Ciencias Veterinarias. 2014;24(4):355-62. 103. Estévez J, Magaña P, Chippaux JP, Vidal N, Mancilla R, Paniagua JF, et al. Study on the venoms of the principal venomous snakes from French Guiana and the neutralization. Bulletin de la Société de pathologie exotique (1990). 2008;101(4):353-9. 104. Rojas G, Gutiérrez JM, Gené JA, Gómez M, Cerdas L. Neutralization of toxic and enzyme activities of 4 venoms from snakes of Guatemala and Honduras by the polyvalent antivenin produced in Costa Rica. Revista de biología tropical. 1987;35(1):59-67. 105. Ruiz RI, Ruiz LI, Martinezvargas AZ, Arruz MS, Gutierrez JM. TOXICITY AND NEUTRALIZATION OF VENOMS FROM PERUVIAN SNAKES OF THE GENERA BOTHROPS AND LACHESIS (SERPENTES VIPERIDAE). Revista De Biologia Tropical. 1993;41(3A):351-7. |
| Antivenoms not available in the market (n = 36) | 1. Wang B, Liu G, Luo M, Zhang X, Wang Q, Zou S, et al. Preparation and Evaluation of a Horse Antiserum against the Venom of Sea Snake Hydrophis curtus from Hainan, China. Toxins (Basel). 2022;14(4). 2. Bello C, Torrico F, Jiménez JC, Cepeda MV, López MA, Rodríguez-Acosta A. A new approach of immunotherapy against Crotalus snakes envenoming: ostrich (Struthio camelus) egg yolk antibodies (IgY-technology). Investigacion Clinica (Venezuela). 2022;63(1):57-69. 3. Attarde S, Iyer A, Khochare S, Shaligram U, Vikharankar M, Sunagar K. The Preclinical Evaluation of a Second-Generation Antivenom for Treating Snake Envenoming in India. Toxins. 2022;14(3):17. 4. Patra A, Kalita B, Khadilkar MV, Salvi NC, Shelke PV, Mukherjee AK. Assessment of quality and pre-clinical efficacy of a newly developed polyvalent antivenom against the medically important snakes of Sri Lanka. Sci Rep. 2021;11(1):17. 5. Archundia IG, de la Rosa G, Olvera F, Calderón A, Benard-Valle M, Alagón A, et al. Assessment of neutralization of Micrurus venoms with a blend of anti-Micrurus tener and anti-ScNtx antibodies. Vaccine. 2021;39(6):1000-6. 6. Ratanabanangkoon K, Tan KY, Pruksaphon K, Klinpayom C, Gutiérrez JM, Quraishi NH, et al. A pan-specific antiserum produced by a novel immunization strategy shows a high spectrum of neutralization against neurotoxic snake venoms. Scientific reports. 2020;10(1):11261. 7. Rasoulinasab F, Rasoulinasab M, Shahbazzadeh D, Asadi A, Kaboli M. Comparison of venom from wild and long-term captive Gloydius caucasicus and the neutralization capacity of antivenom produced in rabbits immunized with captive venom. Heliyon. 2020;6(12):e05717. 8. Pruksaphon K, Tan KY, Tan CH, Simsiriwong P, Gutiérrez JM, Ratanabanangkoon K. An in vitro α-neurotoxin-nAChR binding assay correlates with lethality and in vivo neutralization of a large number of elapid neurotoxic snake venoms from four continents. PLoS Negl Trop Dis. 2020;14(8):e0008581. 9. Nguyen VT, Vu TTH, Hoang VT, Nguyen TV, Lam TD, Nguyen KC, et al., editors. Immunological properties of the chicken egg yolk (IgY) antibodies against Vietnamese cobra Naja Naja venom. IOP Conference Series: Materials Science and Engineering; 2020. 10. Nazari A, Samianifard M, Rabie H, Mirakabadi AZ. Recombinant antibodies against Iranian cobra venom as a new emerging therapy by phage display technology. Journal of Venomous Animals and Toxins Including Tropical Diseases. 2020;26. 11. Makran B, Fahmi L, Boussada L, Oukkache N, Chgoury F, Benomar H, et al. Comparative toxicological characterization of venoms of Cerastes cerastes and Macrovipera mauritanica from Morocco and neutralization by monospecific antivenoms. Toxin Reviews. 2020;39(4):382-96. 12. Ponce-López R, Neri-Castro E, Borja M, Strickland JL, Alagón A. Neutralizing potency and immunochemical evaluation of an anti-Crotalus mictlantecuhtli experimental serum. Toxicon. 2020;187:171-80. 13. Leiva CL, Cangelosi A, Mariconda V, Farace M, Geoghegan P, Brero L, et al. IgY-based antivenom against Bothrops alternatus: Production and neutralization efficacy. Toxicon. 2019;163:84-92. 14. Castillo-Beltrán MC, Hurtado-Gómez JP, Corredor-Espinel V, Ruiz-Gómez FJ. A polyvalent coral snake antivenom with broad neutralization capacity. PLoS Negl Trop Dis. 2019;13(3):e0007250. 15. Liu JH, He QY, Wang WW, Zhou B, Li B, Zhang YF, et al. Preparation and neutralization efficacy of IgY antibodies raised against Deinagkistrodon acutus venom. Journal of Venomous Animals and Toxins Including Tropical Diseases. 2017;23(1). 16. Kadali R, Kadiyala G, Gurunathan J. Pre-clinical assessment of the effectiveness of modified polyvalent antivenom in the neutralization of Naja naja venom toxicity. Biotechnology and Applied Biochemistry. 2016;63(6):827-33. 17. Duan HL, He QY, Zhou B, Wang WW, Li B, Zhang YZ, et al. Anti-Trimeresurus albolabris venom IgY antibodies: Preparation, purification and neutralization efficacy. Journal of Venomous Animals and Toxins Including Tropical Diseases. 2016;22(1). 18. Laustsen AH, Gutiérrez JM, Lohse B, Rasmussen AR, Fernández J, Milbo C, et al. Snake venomics of monocled cobra (Naja kaouthia) and investigation of human IgG response against venom toxins. Toxicon. 2015;99:23-35. 19. Venkatesan C, Sarathi M, Balasubramanaiyan G, Vimal S, Madan N, Sundar Raj N, et al. Neutralization of cobra venom by cocktail antiserum against venom proteins of cobra (Naja naja naja). Biologicals. 2014;42(1):8-21. 20. Bhattacharya S, Chakraborty M, Mukhopadhyay P, Kundu PP, Mishra R. Viper and Cobra Venom Neutralization by Alginate Coated Multicomponent Polyvalent Antivenom Administered by the Oral Route. PLoS Neglected Tropical Diseases. 2014;8(8). 21. Archundia IG, de Roodt AR, Ramos-Cerrillo B, Chippaux JP, Olguín-Pérez L, Alagón A, et al. Neutralization of Vipera and Macrovipera venoms by two experimental polyvalent antisera: A study of paraspecificity. Toxicon. 2011;57(7-8):1049-56. 22. Gutiérrez JM, Sanz L, Flores-Díaz M, Figueroa L, Madrigal M, Herrera M, et al. Impact of regional variation in Bothrops asper snake venom on the design of antivenoms: Integrating antivenomics and neutralization approaches. Journal of Proteome Research. 2010;9(1):564-77. 23. Fernández GP, Segura A, Herrera M, Velasco W, Solano G, Gutiérrez JM, et al. Neutralization of Bothrops mattogrossensis snake venom from Bolivia: Experimental evaluation of llama and donkey antivenoms produced by caprylic acid precipitation. Toxicon. 2010;55(2-3):642-5. 24. Arce-Estrada V, Azofeifa-Cordero G, Estrada R, Alape-Girón A, Flores-Díaz M. Neutralization of venom-induced hemorrhage by equine antibodies raised by immunization with a plasmid encoding a novel P-II metalloproteinase from the lancehead pitviper Bothrops asper. Vaccine. 2009;27(3):460-6. 25. Casasola A, Ramos-Cerrillo B, de Roodt AR, Carbajal Saucedo A, Chippaux JP, Alagón A, et al. Paraspecific neutralization of the venom of African species of cobra by an equine antiserum against Naja melanoleuca: a comparative study. Toxicon. 2009;53(6):602-8. 26. Meenatchisundaram S, Parameswari G, Michael A, Ramalingam S. Neutralization of the pharmacological effects of Cobra and Krait venoms by chicken egg yolk antibodies. Toxicon. 2008;52(2):221-7. 27. Meenatchisundaram S, Parameswari G, Michael A, Ramalingam S. Studies on pharmacological effects of Russell's viper and Saw-scaled viper venom and its neutralization by chicken egg yolk antibodies. International Immunopharmacology. 2008;8(8):1067-73. 28. Rodriguez JP, De Marzi M, Maruñak S, Malchiodi EL, Leiva LC, Acosta O. Rabbit IgG antibodies against phospholipase A2 from Crotalus durissus terrificus neutralize the lethal activity of the venom. Medicina. 2006;66(6):512-6. 29. Laing GD, Renjifo JM, Ruiz F, Harrison RA, Nasidi A, Gutierrez JM, et al. A new Pan African polyspecific antivenom developed in response to the antivenom crisis in Africa. Toxicon. 2003;42(1):35-41. 30. Oshima-Franco Y, Hyslop S, Prado-Franceschi J, Cruz-Höfling MA, Rodrigues-Simioni L. Neutralizing capacity of antisera raised in horses and rabbits against Crotalus durissus terrificus (South American rattlesnake) venom and its main toxin, crotoxin. Toxicon. 1999;37(10):1341-57. 31. León G, Stiles B, Alape A, Rojas G, Gutiérrez JM. Comparative study on the ability of IgG and F(ab')2 antivenoms to neutralize lethal and myotoxic effects induced by Micrurus nigrocinctus (coral snake) venom. American Journal of Tropical Medicine and Hygiene. 1999;61(2):266-71. 32. Fernandes I, Takehara HA, Santos ACR, Cormont F, Latinne D, Bazin H, et al. Neutralization of bothropic and crotalic venom toxic activities by IgG(T) and IgGa subclasses isolated from immune horse serum. Toxicon. 1997;35(6):931-6. 33. Bolanos R, Cerdas L, Abalos JW. Venoms of coral snakes (Micrurus Spp.): Report on a multivalent antivenin for the Americas. Bulletin of the Pan American Health Organization. 1978;12(1):23-7. 34. Cohen P, Dawson JH, Seligmann EB, Jr. Cross-neutralization of Micrurus fulvius fulvius (coral snake) venom by anti-Micrurus carinicauda dumerilii serum. Am J Trop Med Hyg. 1968;17(2):308-10. 35. Hsu JC, Chuang LY, Chang CC. [Preparation and characterization of antibodies against Bungarus multicinctus venom by liposomes]. Gaoxiong Yi Xue Ke Xue Za Zhi. 1992;8(6):320-6. 36. Mendoza JC, Vivas D, Rodríguez E, Inga R, Sandoval G, Lazo F, et al. Experimental efficacy of igy antibodies produced in eggs against the venom of the peruvian snake: Bothrops atrox. Revista Peruana de Medicina Experimental y Salud Publica. 2012;29(1):69-75. |
| Not report ED50 (n = 34) | 1. Rashmi U, Khochare S, Attarde S, Laxme RRS, Suranse V, Martin G, et al. Remarkable intrapopulation venom variability in the monocellate cobra (Naja kaouthia) unveils neglected aspects of India's snakebite problem. J Proteomics. 2021:104256. 2. Kadkhodazadeh M, Rajabibazl M, Motedayen M, Shahidi S, Veisi Malekshahi Z, Rahimpour A, et al. Isolation of Polyclonal Single-Chain Fragment Variable (scFv) Antibodies Against Venomous Snakes of Iran and Evaluation of Their Capability in Neutralizing the Venom. Iran J Pharm Res. 2020;19(3):288-96. 3. Méndez R, Bonilla F, Sasa M, Dwyer Q, Fernández J, Lomonte B. Proteomic profiling, functional characterization, and immunoneutralization of the venom of Porthidium porrasi, a pitviper endemic to Costa Rica. Acta Tropica. 2019;193:113-23. 4. Lippa E, Török F, Gómez A, Corrales G, Chacón D, Sasa M, et al. First look into the venom of Roatan Island's critically endangered coral snake Micrurus ruatanus: Proteomic characterization, toxicity, immunorecognition and neutralization by an antivenom. Journal of Proteomics. 2019;198:177-85. 5. Ainsworth S, Petras D, Engmark M, Süssmuth RD, Whiteley G, Albulescu LO, et al. The medical threat of mamba envenoming in sub-Saharan Africa revealed by genus-wide analysis of venom composition, toxicity and antivenomics profiling of available antivenoms. Journal of Proteomics. 2018;172:173-89. 6. Lauridsen LP, Laustsen AH, Lomonte B, Gutiérrez JM. Exploring the venom of the forest cobra snake: Toxicovenomics and antivenom profiling of Naja melanoleuca. Journal of Proteomics. 2017;150:98-108. 7. Abdou F, Denshary EE, Shaaban E, Mohamed M. Assessment of the neutralizing potency of antisera raised against native and -irradiated Naja nigricollis (black-necked spitting cobra) venom in rabbits, concerning its cardiotoxic effect. Human & Experimental Toxicology. 2017;36(12):1335-44. 8. Tan CH, Liew JL, Tan KY, Tan NH. Genus Calliophis of Asiatic coral snakes: A deficiency of venom cross-reactivity and neutralization against seven regional elapid antivenoms. Toxicon. 2016;121:130-3. 9. Lee CH, Lee YC, Leu SJ, Lin LT, Chiang JR, Hsu WJ, et al. Production and Characterization of Neutralizing Antibodies against Bungarus multicinctus Snake Venom. Appl Environ Microbiol. 2016;82(23):6973-82. 10. Venkatesan C, Sarathi M, Balasubramanian G, Saravanan A, Vimal S, Madan N, et al. Detection and neutralization of cobra venom using rabbit antiserum in experimental envenomated mice. Hum Exp Toxicol. 2014;33(7):772-82. 11. Gowtham YJ, Mahadeswaraswamy YH, Girish KS, K K. Cross-reactivity and neutralization of Indian King cobra (Ophiophagus hannah) venom by polyvalent and monovalent antivenoms. Int Immunopharmacol. 2014;21(1):148-55. 12. Barry JD, Hanson CE, McCall S, Merril J. Evaluation of crotalidae polyvalent fab antivenom for the treatment of Middle Eastern and South Asian Viperidae envenomation in a murine model (Mus musculus). Clinical Toxicology. 2012;50(7):692. 13. Shashidharamurthy R, Kemparaju K. Region-specific neutralization of Indian cobra (Naja naja) venom by polyclonal antibody raised against the eastern regional venom: A comparative study of the venoms from three different geographical distributions. International Immunopharmacology. 2007;7(1):61-9. 14. Ismail M, Al-Ahaidib MS, Abdoon N, Abd-Elsalam MA. Preparation of a novel antivenom against Atractaspis and Walterinnesia venoms. Toxicon. 2007;49(1):8-18. 15. Richardson WH, 3rd, Tanen DA, Tong TC, Betten DP, Carstairs SD, Williams SR, et al. North American coral snake antivenin for the neutralization of non-native elapid venoms in a murine model. Acad Emerg Med. 2006;13(2):121-6. 16. Kumar AV, Gowda TV. Novel non-enzymatic toxic peptide of Daboia russelii (Eastern region) venom renders commercial polyvalent antivenom ineffective. Toxicon. 2006;47(4):398-408. 17. Richardson WH, 3rd, Tanen DA, Tong TC, Betten DP, Carstairs SD, Williams SR, et al. Crotalidae polyvalent immune Fab (ovine) antivenom is effective in the neutralization of South American viperidae venoms in a murine model. Ann Emerg Med. 2005;45(6):595-602. 18. Wisniewski MS, Hill RE, Havey JM, Bogdan GM, Dart RC. Australian tiger snake (Notechis scutatus) and Mexican coral snake (Micruris species) antivenoms prevent death from United States coral snake (Micrurus fulvius fulvius) venom in a mouse model. Journal of Toxicology - Clinical Toxicology. 2003;41(1):7-10. 19. Pakmanee N, Khow O, Wongtongkam N, Tamotsu OS, Sitprija V. Efficacy and cross reactivity of Thai green pit viper antivenom among venoms of Trimeresurus species in Thailand and Japan. Journal of Natural Toxins. 1998;7(2):173-83. 20. Al-Asmari AK, Al-Abdulla IH, Crouch RG, Smith DC, Sjostrom L. Assessment of an ovine antivenom raised against venom from the desert black cobra (Walterinnesia aegyptia). Toxicon. 1997;35(1):141-5. 21. Tan NH, Choy SK, Chin KM, Ponnudurai G. Cross-reactivity of monovalent and polyvalent Trimeresurus antivenoms with venoms from various species of Trimeresurus (lance-headed pit viper) snake. Toxicon. 1994;32(7):849-53. 22. Mandal M, Hati RN, Hati AK. Neutralization of pathophysiological manifestations of Russell's viper envenoming by antivenom raised against gamma-irradiated toxoid. Toxicon. 1993;31(2):213-6. 23. Dart RC, O’Brien PC, Garcia RA, Jarchow JC, McNally J. Neutralization of Micrurus distans distans venom by antivenin (Micrurus fulvius). Journal of Wilderness Medicine. 1992;3(4):377-81. 24. Middlebrook JL. Cross-neutralizations of phospholipase A2 neurotoxins from snake venoms. Toxicon. 1991;29(12):1481-7. 25. Kornalik F, Taborska E. Cross reactivity of mono- and polyvalent antivenoms with Viperidae and Crotalidae snake venoms. Toxicon. 1989;27(10):1135-42. 26. Lomonte B, Gutierrez JM, Mata E. Isolation from a polyvalent antivenom of antibodies to a myotoxin in Bothrops asper snake venom. Toxicon. 1985;23(5):807-13. 27. Minton SA. Neutralization of old world viper venoms by American pit viper antivenin. Toxicon. 1976;14(2):146-8. 28. Baxter EH, Gallichio HA. Cross-neutralization by tiger snake (Notechis scutatus) antivenene and sea snake (Enhydrina schistosa) antivenene against several sea snake venoms. Toxicon. 1974;12(3):273-8. 29. Tu AT, Ganthavorn S. Immunological properties and neutralization of sea-snake venoms from Southeast Asia. Am J Trop Med Hyg. 1969;18(1):151-4. 30. Ganthavorn S. Toxicities of Thailand snake venoms and neutralization capacity of antivenin. Toxicon : official journal of the International Society on Toxinology. 1969;7(3):239-41. 31. Minton Jr SA. Paraspecific protection by elapid and sea snake antivenins. Toxicon. 1967;5(1):47-55. 32. Moroz C, de Vries A, Goldblum N. Preparation of an antivenin against Vipera palestinae venom with high antineurotoxic potency. Toxicon. 1966;4(3):205-8. 33. Flowers HH. A comparison of the neutralization ability of a heterologous vs. homologous coral snake (Micrurus fulvius) venom. Am J Trop Med Hyg. 1966;15(6):1003-6. 34. Keegan HL, Whittemore FW, Jr., Flanigan JF. Heterologous antivenin in neutralization of North American coral snake venom. Public Health Rep. 1961;76(6):540-2. |
| Method does not comply with WHO guideline (n = 14) | 1. Sandesha VD, Darshan B, Tejas C, Girish KS, Kempaiah K. A comparative cross-reactivity and paraspecific neutralization study on Hypnale hypnale, Echis carinatus, and Daboia russelii monovalent and therapeutic polyvalent anti-venoms. Plos Neglect Trop Dis. 2022;16(3):31. 2. Palasuberniam P, Chan YW, Tan KY, Tan CH. Snake Venom Proteomics of Samar Cobra (Naja samarensis) from the Southern Philippines: Short Alpha-Neurotoxins as the Dominant Lethal Component Weakly Cross-Neutralized by the Philippine Cobra Antivenom. Front Pharmacol. 2021;12:15. 3. Okumu MO, Mbaria JM, Gikunju JK, Mbuthia PG, Madadi VO, Ochola FO. Enzymatic activity and brine shrimp lethality of venom from the large brown spitting cobra (Naja ashei) and its neutralization by antivenom. BMC research notes. 2020;13(1):325. 4. Tan NH, Wong KY, Tan CH. Venomics of Naja sputatrix, the Javan spitting cobra: A short neurotoxin-driven venom needing improved antivenom neutralization. Journal of Proteomics. 2017;157:18-32. 5. Liu CC, You CH, Wang PJ, Yu JS, Huang GJ, Liu CH, et al. Analysis of the efficacy of Taiwanese freeze-dried neurotoxic antivenom against Naja kaouthia, Naja siamensis and Ophiophagus hannah through proteomics and animal model approaches. PLoS Neglected Tropical Diseases. 2017;11(12). 6. Tan CH, Tan KY, Lim SE, Tan NH. Venomics of the beaked sea snake, Hydrophis schistosus: A minimalist toxin arsenal and its cross-neutralization by heterologous antivenoms. Journal of Proteomics. 2015;126:121-30. 7. Fung HT, Yung WH, Crow P, Lam KK, Ho KKW, Tan KS, et al. Green pit viper antivenom from Thailand and Agkistrodon halys antivenom from China compared in treating Cryptelytrops albolabris envenomation of mice. Hong Kong Medical Journal. 2012;18(1):40-5. 8. Smith MS, Ownby CL. Ability of polyvalent (Crotalidae) antivenin to neutralize myonecrosis, hemorrhage and lethality induced by timber rattlesnake (Crotalus horridus horridus) venom. Toxicon. 1985;23(3):409-24. 9. Liu CC, Chou YS, Chen CY, Liu KL, Huang GJ, Yu JS, et al. Pathogenesis of local necrosis induced by Naja atra venom: Assessment of the neutralization ability of Taiwanese freeze-dried neurotoxic antivenom in animal models. PLoS Negl Trop Dis. 2020;14(2):e0008054. 10. Frauches TS, Petretski JH, Arnholdt AC, Lasunskaia EB, de Carvalho EC, Kipnis TL, et al. Bothropic antivenom based on monoclonal antibodies, is it possible? Toxicon. 2013;71:49-56. 11. Lee CH, Liu CI, Leu SJ, Lee YC, Chiang JR, Chiang LC, et al. Chicken antibodies against venom proteins of Trimeresurus stejnegeri in Taiwan. J Venom Anim Toxins Incl Trop Dis. 2020;26:e20200056. 12. Iddon D, Hommel M, Theakston RDG. Characterisation of a monoclonal antibody capable of neutralising the haemorrhagic activity of West African Echis carinatus (carpet viper) venom. Toxicon. 1988;26(2):167-79. 13. Herrera M, Collaco RDD, Villalta M, Segura A, Vargas M, Wright CE, et al. Neutralization of the neuromuscular inhibition of venom and taipoxin from the taipan (Oxyuranus scutellatus) by F(ab ') 2 and whole IgG antivenoms. Toxicology Letters. 2016;241:175-83. 14. Paul K, Manjula J, Deepa EP, Selvanayagam ZE, Ganesh KA, Rao PVS. Anti-Echis carinatus venom antibodies from chicken egg yolk: Isolation, purification and neutralization efficacy. Toxicon. 2007;50(7):893-900. |
| Review article (n = 9) | 1. Choraria A, Somasundaram R, Janani S, Rajendran S, Oukkache N, Michael A. Chicken egg yolk antibodies (IgY)-based antivenom for neutralization of snake venoms: a review. Toxin Rev. 2021. 2. Gutiérrez JM, Vargas M, Segura Á, Herrera M, Villalta M, Solano G, et al. In Vitro Tests for Assessing the Neutralizing Ability of Snake Antivenoms: Toward the 3Rs Principles. Frontiers in Immunology. 2020;11. 3. Ainsworth S, Menzies SK, Casewell NR, Harrison RA. An analysis of preclinical efficacy testing of antivenoms for sub-Saharan Africa: Inadequate independent scrutiny and poor-quality reporting are barriers to improving snakebite treatment and management. PLoS Neglected Tropical Diseases. 2020;14(8):1-25. 4. Neri-Castro E, Bénard-Valle M, Archundia I, Calvete JJ, Alagón A. Implications of snake venom variation on antivenom neutralization: The case of North American vipers. Toxicon. 2020;177:S25. 5. Kalita B, Mackessy SP, Mukherjee AK. Proteomic analysis reveals geographic variation in venom composition of Russell’s Viper in the Indian subcontinent: implications for clinical manifestations post-envenomation and antivenom treatment. Expert Review of Proteomics. 2018;15(10):837-49. 6. Gutiérrez JM. Preclinical assessment of the neutralizing efficacy of snake antivenoms in Latin America and the Caribbean: A review. Toxicon. 2018;146:138-50. 7. Padula AM, Winkel KD. Successful use of camelid (alpaca) antivenom to treat a potentially lethal tiger snake (Notechis scutatus) envenomation in a dog. Toxicon. 2016;114:59-64. 8. Gutiérrez JM, Lomonte B, Sanz L, Calvete JJ, Pla D. Immunological profile of antivenoms: Preclinical analysis of the efficacy of a polyspecific antivenom through antivenomics and neutralization assays. Journal of Proteomics. 2014;105:340-50. 9. Lomonte B, León G, Angulo Y, Rucavado A, Núñez V. Neutralization of Bothrops asper venom by antibodies, natural products and synthetic drugs: Contributions to understanding snakebite envenomings and their treatment. Toxicon. 2009;54(7):1012-28. |
